# A genome‑wide approach to the systematic and comprehensive analysis of LIM gene family in sorghum (*Sorghum bicolor* L.)

**DOI:** 10.5808/gi.23007

**Published:** 2023-09-27

**Authors:** Md. Abdur Rauf Sarkar, Salim Sarkar, Md Shohel Ul Islam, Fatema Tuz Zohra, Shaikh Mizanur Rahman

**Affiliations:** 1 Department of Genetic Engineering and Biotechnology, Faculty of Biological Science and Technology, Jashore University of Science and Technology, Jashore 7408, Bangladesh; 2 Department of Genetic Engineering and Biotechnology, Faculty of Biological Sciences, University of Rajshahi, Rajshahi 6205, Bangladesh

**Keywords:** characterization, genome-wide, identification, *in silico*, LIM gene, sorghum

## Abstract

The LIM domain-containing proteins are dominantly found in plants and play a significant role in various biological processes such as gene transcription as well as actin cytoskeletal organization. Nevertheless, genome-wide identification as well as functional analysis of the LIM gene family have not yet been reported in the economically important plant sorghum (*Sorghum bicolor* L.). Therefore, we conducted an *in silico* identification and characterization of LIM genes in *S. bicolor* genome using integrated bioinformatics approaches. Based on phylogenetic tree analysis and conserved domain, we identified five LIM genes in *S. bicolor* (SbLIM) genome corresponding to Arabidopsis LIM (AtLIM) genes. The conserved domain, motif as well as gene structure analyses of the SbLIM gene family showed the similarity within the SbLIM and AtLIM members. The gene ontology (GO) enrichment study revealed that the candidate LIM genes are directly involved in cytoskeletal organization and various other important biological as well as molecular pathways. Some important families of regulating transcription factors such as ERF, MYB, WRKY, NAC, bZIP, C2H2, Dof, and G2-like were detected by analyzing their interaction network with identified SbLIM genes. The cis-acting regulatory elements related to predicted SbLIM genes were identified as responsive to light, hormones, stress, and other functions. The present study will provide valuable useful information about LIM genes in sorghum which would pave the way for the future study of functional pathways of candidate SbLIM genes as well as their regulatory factors in wet-lab experiments.

## Introduction

LIM proteins belong to the family of cysteine-rich proteins (CRPS), which are found in almost all eukaryotic organisms [1]. The name LIM denotes the acronyms of three LIM domain-containing proteins which are LIN11, ISL1, as well as MEC3. ISL1 was first identified in rats, while LIN11 and MEC3 were observed in *Caenorhabditis elegans* [2]. During transcription, LIM domains are actively associated with the process of DNA binding. A minimum of one LIM domain, which is responsible for mediating protein interactions, is present in LIM proteins found in eukaryotic organisms. There have been observations that LIM proteins sometimes include either homologous domains or protein kinase domains. LIM proteins serve two important functions: (1) as transcription factors (TFs), LIM proteins in plants are responsible for the regulation of the gene expression associated with a pathway of phenylpropanoid biosynthesis [3]; and (2) actin-binding proteins, regulating the structure of the actin cytoskeleton. Both of these functions are important for plant survival [4]. The LIM is a zinc coordination domain that contains over 50 amino acids and is abundant in cysteine and histidine [5]. The sequence of the conserved LIM domain is [C-X_2_-C-X_17_-H-X_2_-C]-X_2_-[C-X_2_-C-X_17_-CX_2_-H] which is shared by many species [6].

It has also been discovered that the LIM protein family has a function in cell migration in animals, which suggests that it may have a role in both the development of tumors and metastasis [7]. Among plants, LIM proteins were detected for the first time in sunflowers, where it was shown as exclusively produced in the plant's pollen [8]. Some varieties of plants have been discovered to have LIM proteins. Some of these plants include Arabidopsis [9], tobacco [10], tomato [11], cotton [12], etc. CRP and DAR are two separate subfamilies of LIM proteins. Similar to the CRP (cysteine-rich protein) family in animals, the CRP family has two conserved LIM domains [13]. The C-terminus of LIM proteins that are members of the CRP subfamily is short, and there is a total absence of glycine-rich region after the LIM domain. Based on the patterns of their expression, CRP proteins in plants are classified into two categories: WLIMs (WLIM1 as well as WLIM2), which are found in a wide range of plant tissues; PLIMs (PLIM1 as well as PLIM2) are expressed significantly in pollen tubes [13]. The plant CRP proteins are divided into four subclasses: αLIM1, βLIM1, γLIM2, as well as δLIM2 based on the similarities in their amino acid sequences. The αLIM1 represents PLIM1, WLIM1, as well as FLIM. While γLIM2 and δLIM2 include WLIM2 as well as PLIM2 respectively. On the other hand, βLIM1 is a recently found LIM protein that contributes to the formation of a novel cluster in the phylogenetic tree whose precise function is not yet understood [13]. The LIM subfamily DAR/DA1 proteins are distinguished from one another by their presence of a conserved single LIM domain, single or multiple ubiquitin interaction motif domains, as well as conserved C-terminus. Based upon similarities in their amino acid sequences, DAR/DA1 may be categorized into two classes: class I as well as class II. Only terrestrial plants contain DAR/DA1 proteins [14].

There was an observation of interaction between LIM proteins and actin proteins, and also the participation of LIM in the pathway of phenylpropane secondary metabolism, playing an important function in regulating the development of plant cells as well as stress tolerance [15]. For example, the tobacco (*Nicotiana tabacum*) NtLIM1 protein initiates a crucial gene PAL (phenylalanine ammonia-lyase) related to the metabolism of lignin after binding with the PAL box in the promoter [16]. Besides, not only GhWLIM1a overexpression of upland cotton enhanced the cinnamyl coenzyme A reductase (CCR), 4-coumarate: coenzyme A ligase as well as cinnamyl alcohol dehydrogenase, but also altered the fibroblasts secondary cell walls constitution [17]. Furthermore, overexpression of *PtaGLIMa* may alter poplar xylem development as well as lignin contents [18]. LIM domain proteins are localized in the nucleus, cytoplasm, and cytoskeletal which indicates their involvement in communication between nucleus, and cytoplasm [19]. LIM protein’s nuclear localization is critical for regulating tissue-specific genes as well as differentiation in the cell. For example, in tobacco, LIM1 proteins localized in the nucleus initiate gene expression of glucuronidase reporters [20]. In addition, six LIM domain-containing proteins are found in the actin cytoskeleton in *Arabidopsis thaliana* as well as may bind to actin protein which is controlled by Ca^2+^ binding activity and pH [21]. Recent research has revealed that expression in the LIM gene changes by responding to biotic as well as abiotic stresses like salt, drought, as well as various hormones [14]. Studies have found that in plants, the cytoskeleton performs a very important function in various stress responses [22-24]. The cytoskeleton is associated with various cell life activities, as well as cytoskeleton elements recombination can be a mechanism of abiotic stress tolerance. LIM genes have been found to carry out a valuable function in responding to resisting abiotic stress in plants [14]. As a result, the family of the LIM gene is a very important and interesting topic of research in plant science [25,26].

Sorghum (*Sorghum bicolor* L.) is a C4 type most common perennial cereal crop belonging to the Gramineae family grown all over the world [27]. It is extensively used in the food, feed, brewing, and biofuel industry [28] due to its abundance in vitamins, proteins, polyphenols, and resistant starch [29,30]. It occupied the fifth position all over the world regarding both area cultivated and production among grain crops [31]. Sorghum has evolved into many different varieties as a result of long-term environmental adaptation, but still, there is a big impact of some severe abiotic and biotic stresses on the plant's growth as well as development. For instance, *S. bicolor* plants exhibit decreased weight of single-grain and floret fertility in high temperatures, which lowers yield [32,33]; low temperatures weaken this crop's potential for growth, and frost generally causes significant damage to plants [34]. Although sorghum has a strong root system which helps it endure drought to some extent [35,36], long-term severe drought impacts negatively growth as well as yield [36]. Pests, illnesses, weeds as well as other biotic factors all significantly reduce yields during the production of sorghum [35]. Reduction in sorghum production due to these abiotic, biotic factors makes it necessary to develop a sorghum variety resistant to these factors. As LIM genes are found to be associated with various stress responses in plants [14], it will have significant research and economic value to identify and characterize LIM genes in sorghum.

Whole genome sequencing of *S. bicolor* in 2009 allowed us to subsequently investigate, clone, and validate the LIM genes involved in stress resistance [37]. The genome of *S. bicolor* is 750 Mb long, with approximately 30,000 genes, which is about 75% higher compared to rice [38]. Previous studies identified six LIM proteins in Arabidopsis [13], 10 in foxtail millet [39], six in rice [13], 22 of brassica [40], 14 in pear [41] as well as 12 in poplar [42]. It has been found that a family of LIM proteins may carry out a significant role in various important biological functions in plants, however, still, there are no genome-wide studies on LIM genes in sorghum to date. As a result, we identified and *in silico* characterized the LIM gene family in sorghum genome. The goal of our present study was to accumulate valuable information to further study the plants LIM genes as well as a solid ground for future research of LIM genes in sorghum.

## Methods

### Genome-wide identification of LIM genes in sorghum

The whole genome and protein sequences of *S. bicolor* were retrieved from a database called Phytozome v13.0 (http://www.phytozome.net) (Supplementary Figs. 1–8). For retrieving the all-LIM genes in *S. bicolor*, all the published sequences as well as annotation information of the LIM gene family of Arabidopsis were obtained from the TAIR database (http://www.arabidopsis.org/). The domains of the LIM protein family were collected from the Pfam database (http://pfam.janelia.org/) using Hidden Markov Model and subsequently, the typical LIM domains were utilized to find out LIM genes in sorghum using a program named BlastP. At last, the probable candidate LIM gene sequence of *S. bicolor* was obtained through the databases Pfam (http://pfam.xfam.org/family), NCBI-CDD (https://www.ncbi.nlm.nih.gov/cdd/) as well as SMART (http://smart.embl-heidelberg.de/) for prediction of conserved domains of the protein and utilized for subsequent analysis.

The gene length, open reading frame, primary transcript, and chromosomal location of the predicted genes were obtained from the genome database of *S. bicolor* stored in the Phytozome database. In addition, the basic physiochemical properties of LIM proteins of sorghum such as the number of amino acids, molecular weight, values of isoelectric points (pI) as well as grand average hydrophilicity (GRAVY) values of identified LIM proteins were deeply studied by an online tool ExPASy (https://web.expasy.org/protparam/).

### Phylogenetic analysis and multiple sequence alignment

A phylogenetic tree was built using candidate LIM protein sequences of sorghum and previously identified LIM protein sequences of 10 different plant species, including Arabidopsis (*Arabidopsis thaliana* L.), tobacco (*Nicotiana tabacum* L.), medicago (*Medicago truncatula* L.), soybean (*Glycine max* L.), tomato (*Solanum lycopersicum* L.), quinoa (*Chenopodium quinoa* L.), maize (*Zea mays* L.), rice (*Oryza sativa* L.), and wheat (*Triticum aestivum* L.). The MEGA 11.0 software was used to import all the LIM protein sequences, and the Clustal W method with default parameters as well as bootstrap value 1,000 was utilized to perform multiple sequence alignment (MSA). A phylogenetic tree was built based on the Neighbor-joining method [43] using the Clustal Omega approach (https://www.ebi.ac.uk/Tools/msa/clustalo/) [44]. Additionally, thousands of bootstrap replicates were used to find out the evolutionary relationships, and evolutionary distances were predicted by using the Equal Input method [45]. The MSA was performed against sequences of LIM proteins of sorghum as well as Arabidopsis using Clustal X (version 2.1) [46], Clustal W method [47], and MEGA 11.0 software [48].

### Analysis of conserved functional domains and motifs

The conserved functional domains of predicted LIM genes in sorghum and previously identified LIM genes of Arabidopsis were investigated using the online databases Pfam, NCBI-CDD, and SMART. Additionally, conserved structural motifs in proteins of sorghum and Arabidopsis were predicted using an online bioinformatics approach called Multiple Expectation Maximization for Motif Elicitation (MEME-Suite v5.3.3) [49]. This MEME analysis was performed by utilizing some specific parameters such as (1) maximum motif number of 20 as well as (2) optimum motif length of ≥6 and ≤50.

### Analysis of gene structure and chromosomal localization

The structure (exon-intron configuration) of LIM genes in sorghum and Arabidopsis was studied by using the online tool Gene Structure Display Server (GSDS2.0: https://gsds.cbi.pku.edu.cn) [50]. Besides, chromosomal localizations of the candidate LIM genes of sorghum were predicted using an online bioinformatics tool called MapGene2Chromosome V2 (http://mg2c.iask.in/mg2c_v2.0/) [51].

### Subcellular localization and gene ontology analysis

The subcellular localizations of the candidate LIM proteins were predicted into different cell organelles by the online tool plant subcellular localization integrative predictor (PSI) (http://bis.zju.edu.cn/psi/) [52]. Gene ontology (GO) enrichment analysis was performed for predicting the functions of identified LIM genes in sorghum utilizing the online tool Plant Transcription Factor Database (PlantTFDB, http://planttfdb.cbi.pku.edu.cn//) [53].

### Analysis of regulatory network between TFs and LIM genes in *S. bicolor*

The online tool PlantTFDB 4.0 (http://planttfdb.cbi.pku.edu.cn//) [53] was used to predict various important TFs related to the candidate LIM genes. In addition, the interaction network between the predicted LIM genes and TFs was built and displayed by another online tool called Cytoscape 3.9.1 [54].

### Analysis of cis-acting regulatory elements of promoter

The cis-acting regulatory elements (CAREs) related to different factors responsive were predicted by analyzing 2.0 kb upstream sequences from the start codon of the candidate LIM genes using a web-based prediction tool called Signal Scan search program of PlantCARE database (http://bioinformatics.psb.ugent.be/webtools/plantcare/html/) [55].

## Results and Discussion

### Identification as well as physiochemical properties of LIM genes in sorghum

Plants encountered a variety of biotic, abiotic stresses at the time of growth and development. At these adverse conditions, various TFs carry out vital roles in functioning. Several biotic, abiotic stresses trigger different TFs of LIM genes. In this study, five LIM genes were detected in sorghum by analyzing the whole genome sequence of sorghum compared with LIM genes of Arabidopsis (Table 1). We named the genes SbLIM1-SbLIM5 based on the position of each candidate LIM gene in the sorghum chromosome. The length of SbLIM genes varies from 1,488 bp to 8,120 bp. The range of coding sequence length of SbLIMs is 585 bp to 4,428 bp. LIM proteins amino acids number ranges from 195 to 1,476 and their molecular weights vary between 21.615 kDa and 158.98 kDa (Table 1). Theoretical pI (isoelectric point) values of SbLIM proteins vary from 4.63 to 8.98 and the GRAVY was found below 0, which suggests that all SbLIM proteins are hydrophilic. Previous studies identified six LIM genes in both Arabidopsis and rice [13], 12 in poplar [42], 10 in foxtail millet [39], 14 in pear [41] as well as 22 in brassica [40] which suggests that LIM gene’s numbers vary among different plant species.

### Phylogenetic tree analysis

**Fig. 1.**
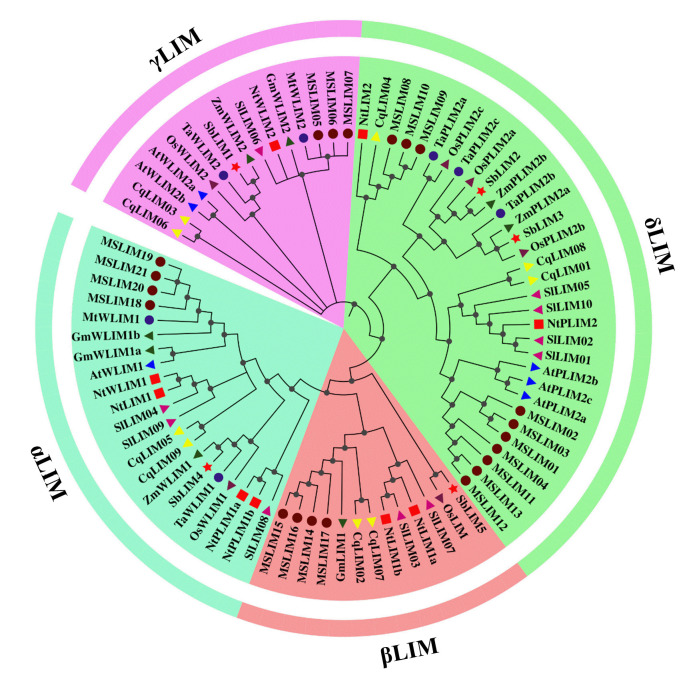
Phylogenetic tree displaying the evolutionary relationships of sorghum LIM proteins to that of Arabidopsis, wheat, rice, tobacco, corn, tomato, soybean, Medicago, quinoa, and alfalfa. The name of each LIM protein starts with a species acronym: At, *Arabidopsis thaliana*; MS, *Medicago sativa* L; Mt, *Medicago truncatula*; Nt, *Nicotiana tabacum*; Sl, *Solanum lycopersicum*; Gm, *Glycine max*; Cq, *Chenopodium quinoa*; Os, *Oryza sativa*; Zm, *Zea mays*; Ta, *Triticum aestivum*.

A complete understanding of a species history of evolution is the primary step for understanding the gene’s evolutionary relationships, which may help study their functions as well as evolution. In a phylogenetic tree, the closer the genes in a cluster are, the more alike the function of genes [56]. To find out the relationships of sorghum’s LIM genes with other plant species, we built a phylogenetic tree by using LIM proteins of sorghum, Arabidopsis, rice, wheat, corn, tobacco, tomato, Medicago, alfalfa, soybean, and quinoa. According to this phylogenetic tree sorghum LIM genes were classified into four individual subfamilies, named αLIM, βLIM, γLIM, and δLIM (Fig. 1). Among these families, αLIM includes one (SbLIM4) protein, while βLIM, δLIM, and γLIM include one (SbLIM5), two (SbLIM2 and SbLIM3), and one (SbLIM1) protein, respectively. The phylogenetic tree revealed that sorghum’s SbLIM1, SbLIM2, and SbLIM4 genes are closely related to *Zea mays* ZmWLIM2, ZmPLIM2b, and ZmWLIM1 genes respectively, while SbLIM3 and SbLIM5 showed a close relationship with rice OsPLIM2b & ZmPLIM2a and OsLIM respectively. These outcomes suggest that sorghum, corn, and rice LIM genes may remain vastly conserved during evolution, which can be passed through the studied species and they may provide similar types of functions.

### MSA analysis

**Fig. 2.**
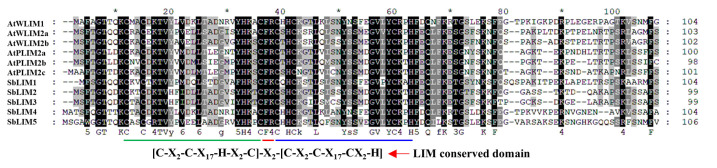
Multiple sequence alignment analysis of LIM gene’s amino acid sequences of sorghum and Arabidopsis by using Clustal X (version 2.1) and Clustal W program in MEGA 11.0.

MSA of predicted sorghum LIM proteins with previously identified Arabidopsis LIM proteins showed that they both contain two LIM domains (LIM1 and LIM2) which are separated by an inter LIM region (Fig. 2). Individual amino acids including most conserved cysteine-histidine residues, were found conserved in LIM domain as well as other regions of the sequence. Immediate flanking regions of LIM1, as well as LIM2 domains, also exhibited sequence similarity. For example, the N-terminus and around 14 amino acid residues of the C-terminal of LIM2 are found well conserved. In addition, Inter LIM region exhibits a similarity of around 15 residues of the C-terminal of LIM1 and 12 residues within the N-terminal of LIM2. Therefore, this study suggests that LIM proteins of sorghum may have similar kinds of functionality to LIM proteins of Arabidopsis.

### Analysis of conserved domain of SbLIM proteins

**Fig. 3.**
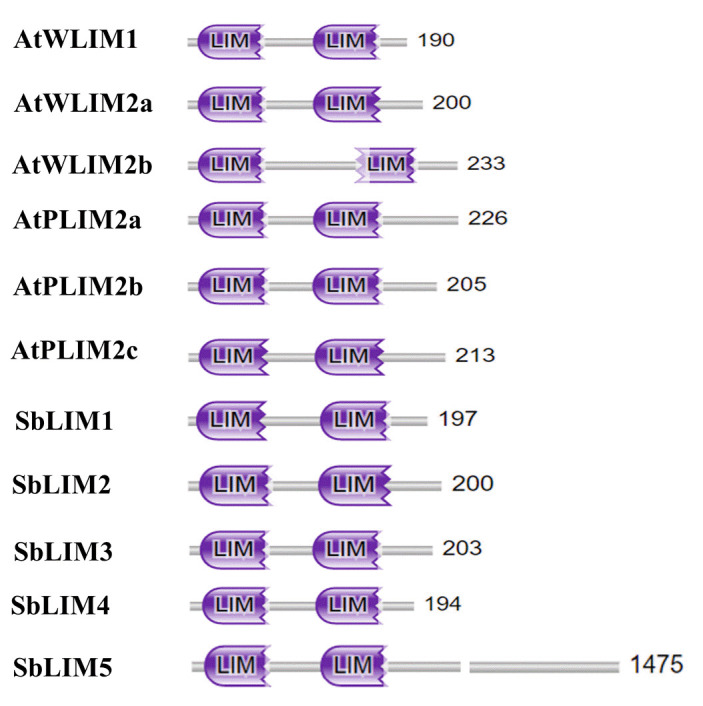
The conserved domains of the predicted SbLIM proteins were drawn by Pfam, SMART, and NCBI-CDD database information.

To deeply analyze the functional domain of five SbLIM proteins, we utilized the protein family database (Pfam, https://pfam.xfam.org/) to get conserved domains of LIM proteins in sorghum. We also analyzed the conserved domain of six LIM proteins of Arabidopsis to compare with the SbLIMs domain. The analysis revealed that all identified SbLIM proteins possess two LIM domains spaced by a small amino acid sequence called inter LIM region which was almost similar to all AtLIM proteins (Fig. 3). Therefore, this investigation suggests that LIM proteins of sorghum will give functionality similar to those of LIM proteins of Arabidopsis.

### Motif analysis of SbLIM genes

**Fig. 4.**
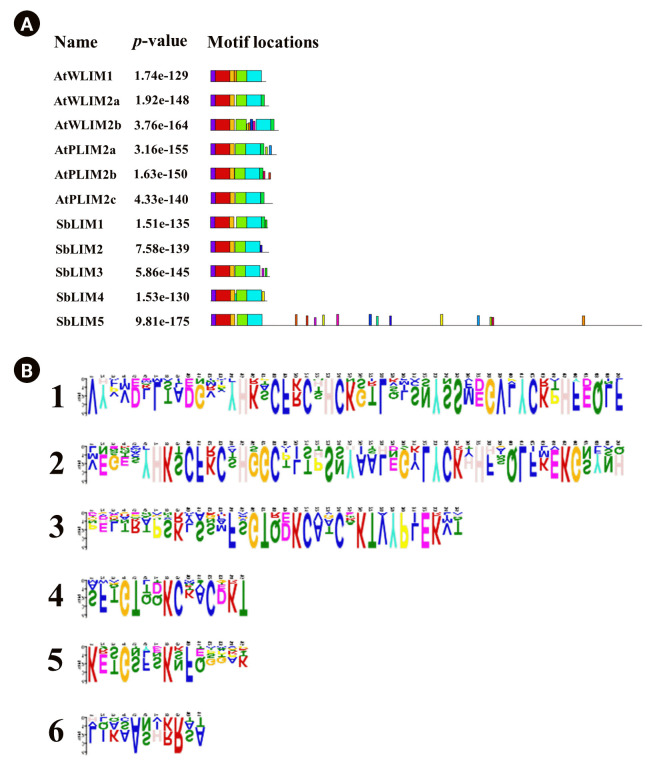
The conserved motifs of the predicted SbLIM protein families are drawn using MEME-suite (a maximum of 20 motifs are analyzed). (A) Different colors indicated individual motifs identified in each SbLIM protein domain. (B) The sequence motifs logos are present in *Sorghum bicolor* SbLIM proteins.

The Multiple Expectation Maximization for Motif Elicitation (MEME) was used to predict motifs for each member of the SbLIM protein families against 20 motifs of Arabidopsis. The number of motifs in five SbLIM proteins (SbLIM1-SbLIM5) is 7, 6, 7, 7, and 18 respectively. On the other hand, motifs number in AtLIM proteins varies from 6 to 10. SbLIM5 contains the highest number of motifs. SbLIM2 was found identical to Arabidopsis AtWLIM1, AtWLIM2a, and AtPLIM2c respectively in terms of motifs and motifs number (6 motifs) which suggests that they will give similar types of functions (Fig. 4). In addition, SbLIM1, SbLIM3, and SbLIM4 may have similar functionality like Arabidopsis AtPLIM2a and AtPLIM2b as they are very close in terms of motif number and configuration. Among 20 motifs, 5 motifs were found highly conserved as they were observed in all LIM proteins of Arabidopsis and sorghum. Collectively, motif analysis suggests that SbLIM proteins may have similar functions to LIM proteins of Arabidopsis.

### Gene structure analysis

**Fig. 5.**
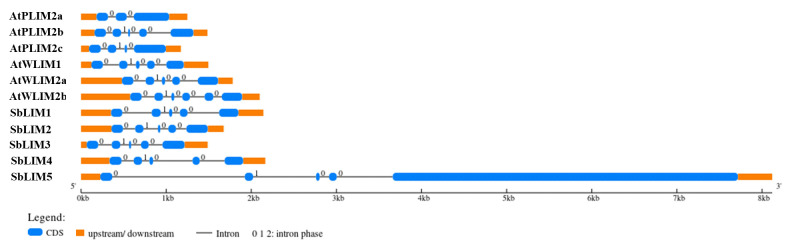
Gene structure of the predicted SbLIM genes in *Sorghum bicolor* with Arabidopsis by using Gene Structure Display Server (GSDS 2.0, http://gsds.cbi.pku.edu.cn/index.php).

The structure of a gene defines its function. For example, by changing the rate of transcription, nuclear output as well as stability of transcription, introns may promote transcription level [57]. For observing the gene structure of predicted SbLIM genes, their exon-intron structure was studied using GSDS 2.0 with the *A. thaliana* respective LIM gene family. The exon-intron structure of predicted SbLIM genes exhibited a significant amount of conservation with AtLIM genes of Arabidopsis as expected (Fig. 5). Gene structure analysis revealed that all SbLIM genes contain five exons and four introns which is similar to those of Arabidopsis AtPLIM2b, AtWLIM1, and AtWLIM2a. While, the number of introns in AtPLIM2a, AtPLIM2c, and AtWLIM2b are 2, 3, and 5, respectively. In addition, exon-intron length and their relative positions were also found quite similar in all SbLIM and AtLIM genes except for the SbLIM05 gene. It was also observed that genes within a cluster in the phylogenetic tree exhibit a considerable amount of similarity in exon-intron configuration, which supports phylogenetic tree construction and also confirmed conserved evolutionary characteristics of the LIM gene family. Most of the plants LIM genes contain 4–5 introns, as well as exon numbers 1, 2, and 4 of LIM genes remain highly conserved [13] which is consistent with the present study. In this study, the exon-intron configuration of SbLIM genes was found a significant amount of similarity to Arabidopsis AtLIM genes which suggests that our candidate SbLIM genes may give similar functions to those of Arabidopsis LIM genes.

### Analysis of chromosomal localization

**Fig. 6.**
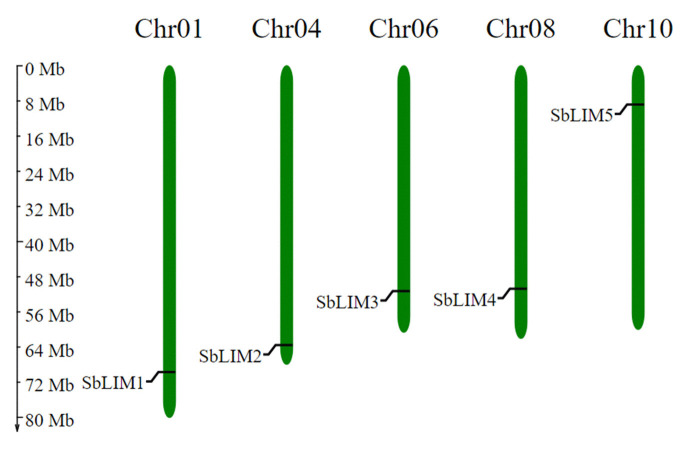
The chromosomal localization of the predicted SbLIM genes. The scale to indicate the chromosomal length is provided on the left.

To find out the chromosomal localizations of predicted five SbLIM genes a detailed chromosomal map was constructed. The chromosomal mapping revealed that SbLIM genes were unevenly distributed among five different chromosomes out of 20 chromosomes of sorghum (Fig. 6). The number of chromosomes corresponds to SbLIM1, SbLIM2, SbLIM3, SbLIM4, and SbLIM5 genes were observed are Chr01, Chr04, Chr06, Chr08, and Chr010, respectively. As they are located in six independent chromosomes, therefore there is no chance for them to be linked and will independently segregate at the time of cell division based on Mendelian genetics [58]. There is still a probability of happening recombination events among these genes. There is a sequence similarity present in different SbLIM genes indicating that the LIM gene has been recombined or duplicated at the time of the evolution of sorghum. During the evolution of plants, gene or chromosomal duplication phenomenon plays a very important role in multiplying family members [59]. All the predicted SbLIM genes were only observed in chromosome numbers Chr01, Chr04, Chr06, Chr08, and Chr10 which may suggest that generation of the sorghum LIM gene family has happened after occurring events of chromosomal duplication.

### Analysis of subcellular localization

**Fig. 7.**
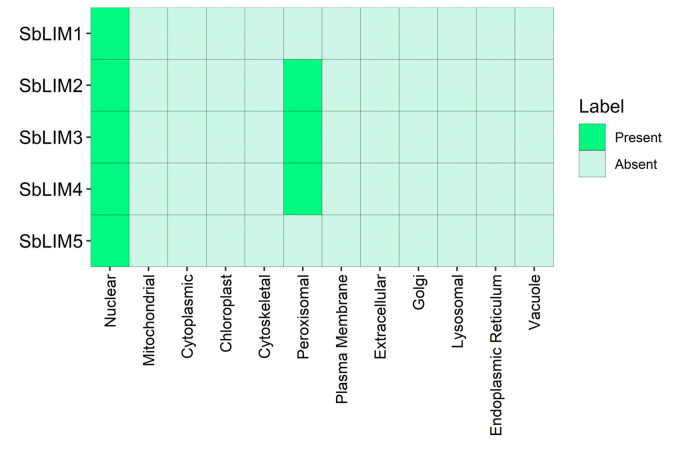
Subcellular localization analysis for the SbLIM proteins. The predicted SbLIM proteins were analyzed in nuclear, mitochondrial, cytoplasmic, chloroplast, cytoskeletal, peroxisomal, plasma membrane, extracellular, golgi, lysosomal, endoplasmic reticulum, and vacuole.

The subcellular localization study exhibited that the identified SbLIM proteins are mainly localized in two subcellular organelles: nucleus and peroxisome (Fig. 7). Notably, all SbLIM proteins were predominantly found in the nucleus. Additionally, SbLIM2, SbLIM3, and SbLIM4 proteins were also observed in the peroxisome. Biological processes and functions in plants are highly dependent on the subcellular locations of specific proteins [60,61]. LIM proteins located in the nucleus are involved in transcriptional regulation of developmental gene expression [16,17,26,62]. Therefore, it can be hypothesized that predicted SbLIM proteins can have significant roles in regulating gene expression of developmental genes too. LIM protein’s subcellular localization in different organelles suggests that they may give different functionality [1] which suggests that SbLIM2, SbLIM3, and SbLIM4 proteins may provide similar kinds of functions due to their localization in the peroxisome.

### GO analysis of SbLIM genes in sorghum

**Fig. 8.**
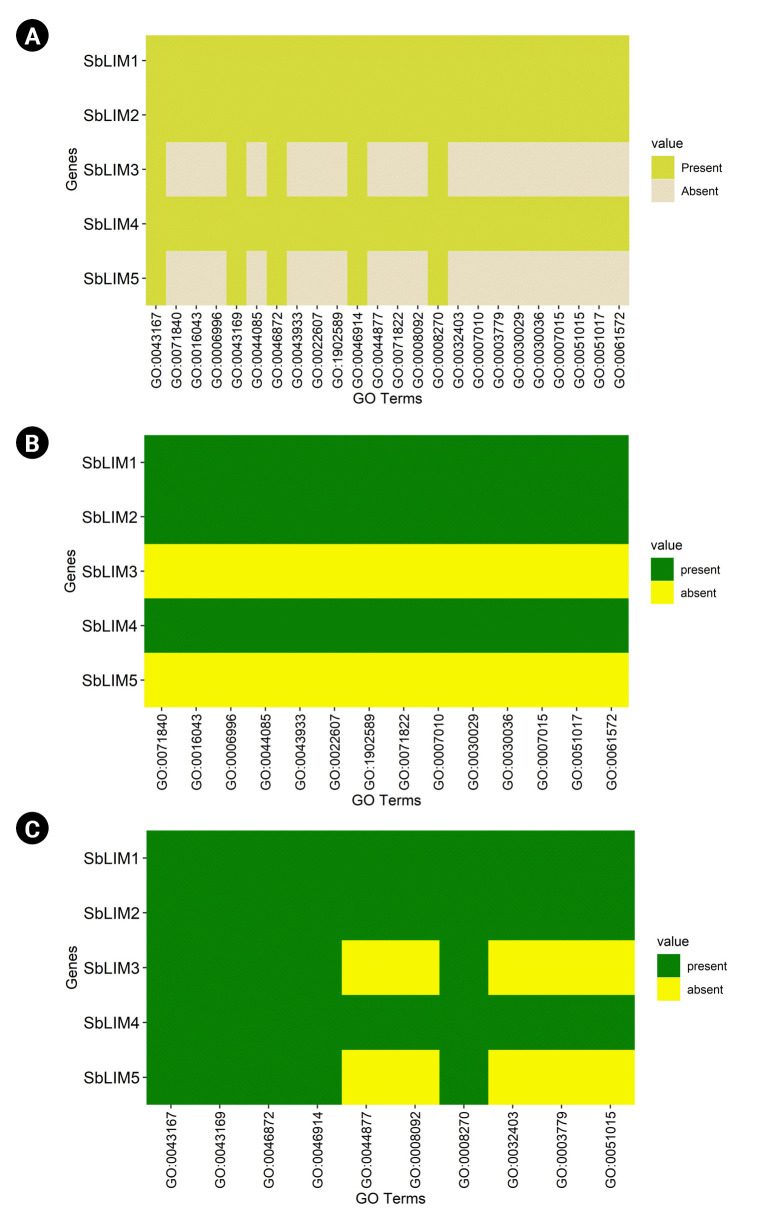
(A) The heatmap for the predicted Gene Ontology (GO) terms corresponding to the predicted SbLIM genes is represented for biological process (B), molecular functions (C), and their association to the SbLIM genes whether they are present or absent.

To predict functions of identified SbLIM genes, GO analysis was used in our study. The analysis predicted a total of 24 GO terms in all SbLIM genes (Fig. 8A, Supplementary Table 1). We sorted the GO terms into two classes based on their function. One class is called biological process (P) as well as the other one is called molecular function (F). The biological process was found more abundant between these two classes which include 14 GO terms (GO:0071840, GO:0016043, GO:0006996, GO:0044085, GO:0043933, GO:1902589, GO:0022607, GO:0071822. GO:0007010, GO:0030029, GO:0030036, GO:0007015, GO:0051017, and GO:0061572) (Fig. 8B). While the class of molecular function includes 10 GO functions (GO:0043167, GO:0044877, GO:0043169, GO:0046872, GO:0008092, GO:0032403, GO:0003779, GO:0008270, and GO:0051015) (Fig. 8C). Out of 24 GO terms, five terms (GO:0043167, GO:0043169, GO:0046872, GO:0046914, and GO:0008270) of the F category were found in all SbLIM genes. While the other 19 GO functions were observed only in three SbLIM genes (SbLIM01, SbLIM02, and SbLIM04) (Supplementary Table 1).

We also performed a p-value analysis of identified GO functions. The results revealed that different GO terms have different p-values. GO:0043167 has a maximum p-value of 0.00523, next GO:0071840 (p = 0.00329) and GO:0016043 (p = 0.00214), then GO:0006996 (p = 0.00037), GO:0043169 (p = 0.00026), etc. (Supplementary Table 1). On the other hand, the p-value of the rest of the GO terms is quite similar. Besides, analysis of p-values against F and P function categories was done individually. We observed that among the F function category, GO:0043167 has the highest p-value of 0.00523, then GO:0043169 (p = 0.00026), next GO:0046872 (p = 0.00025), etc. While among GO terms of P function categories, GO:0071840 was found with the highest p-value of 0.00329, subsequently, GO:0016043 (p = 0.00214), GO:0006996 (p = 0.00037), etc. (Supplementary Table 1). Therefore, this investigation predicts that SbLIM proteins of sorghum may have functions in organ development, cytoskeletal formation, and binding activities with various partners. Thus, after binding with partners like DNA, proteins, and ions SbLIM proteins may regulate the expression of target genes. These results showed a significant amount of consistency with earlier studies related to the LIM protein family in plants [15].

### Analysis of regulatory relationships between TFs and LIM genes in sorghum

TFs carry out a crucial role in a variety of biological functions in living systems, particularly in plants. For example, plant’s responses to various biotic, and abiotic stresses, metabolism, growth, and development as well as defense against different microbial infections [63-67]. In plants, TFs work as a key regulator a molecular switch for various functional genes which express under specific conditions like stresses, growth, as well as development. There is a variety of TFs such as ERF, MYB, Dof (DNA-binding one finger), bZIP, CBF/DREB1, NAC, HSF, AP2/EREBP, WRKY, MIKC_MADS, TGA6, and BOS1 families which present in plants as well as play important functions in response to various biotic abiotic stresses as well as developmental conditions [65,68,69].

**Fig. 9.**
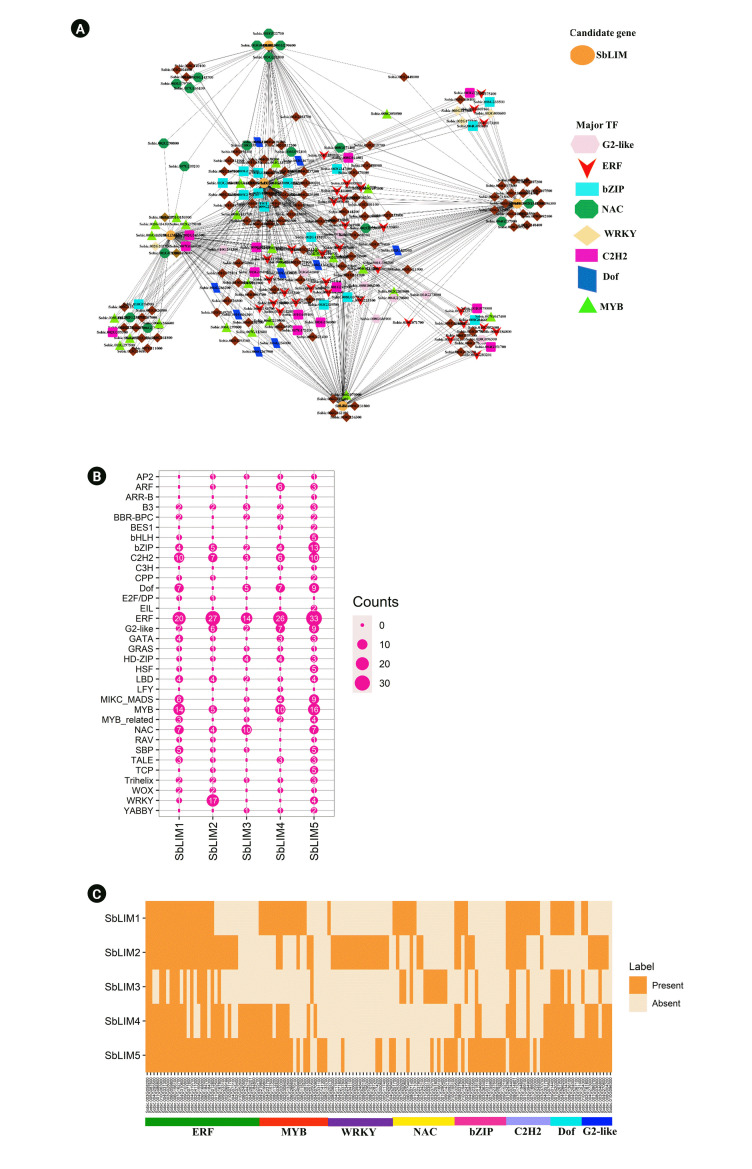
(A) The regulatory network among the transcription factors (TFs) and the predicted SbLIM genes. Each node of the network was colored differently based on SbLIM genes and TFs. SbLIM genes were represented by orange color. Different node symbols were used for different families of TFs. TFs were configured at the hub node level using black. (B) The map represents the related number of TFs with the predicted SbLIM LIM genes. (C) SbLIM gene-mediated sub-network for ERF, MYB, WRKY, NAC, bZIP, C2H2, Dof, and G2-like TFs families which is expressed as Heatmap.

In this study, out of 334 TFs, a total of 224 unique TFs were detected which regulate the identified SbLIM genes (Fig. 9A, Supplementary Table 2). These detected TFs were divided into 34 groups based on TF families. The analyzed network revealed that different families of TFs showed different structures and connected to the candidate SbLIM genes. Such as TFs of the ERF family are dominantly linked to the SbLIM05 gene. Besides, the rest of the four SbLIM genes were also found to be controlled by the ERF family; all of them were also linked to SbLIM05. Additionally, ERF, MYB, WRKY, NAC, bZIP, C2H2, Dof, and G2-like families included a total of 136 TFs which may have an important role in controlling SbLIM genes because they are the major eight families containing 33, 20, 13, 15, 9, 18, 9, and 19 TFs respectively which accounted 60.71% of total 224 identified TFs (Fig. 9B, Supplementary Table 3).

Further, studied the sub-network relationships among TFs as well as candidate SbLIM genes (Fig. 9C, Supplementary Table 4). The results showed that all major TFs families (ERF, MYB, WRKY, NAC, bZIP, C2H2, Dof, and G2-like) are connected to all SbLIM genes in sorghum except for Dof, NAC, and WRKY which are not linked to SbLIM2, SbLIM4, and SbLIM3 and SbLIM4 respectively. In addition, by performing node degree analysis we identified 14 hub TFs which were found to have interactions with all the predicted SbLIM genes. Among 14 hubs TFs, 12 belong to the ERF family, and the rest two TFs belong to the C2H2 family.

The ERF (ethylene response factor) is involved in ethylene (ET) signaling as well as the response pathway in plants which has a characteristic single AP2 domain [70]. ERF also responds to plant hormones with enhanced survival in stressful conditions. Such as some AP2/ERF families show response to plant growth regulators (PGRs) abscisic acid (ABA) as well as ET for stimulating ABA, ET dependent, or independent stress-responsive (SR) related genes [71]. A previous experimental study showed that an ERF (SlERF5/ERF5) helps to enhance adaptation to salt and drought tolerance in tomato [72]. MYB TFs were seen also in high numbers in plants. In Arabidopsis, MYB TFs comprised around 9% of total TFs [73]. This TF family is also connected to various important biological processes in plants, for example, defense as well as stress responses, circadian rhythm, cell fate as well as the identity, floral and seed development, and primary as well as secondary metabolism regulation [73,74]. The C2H2 TFs family encodes proteins that carry out various important functions in growth, development, as well as biotic stress resistance in plants [75]. There has been a considerable amount of evidence showing that the family of bZIP TFs plays key roles in various biological processes in plants, including embryogenesis, seed maturation, organ differentiation, flower as well as vascular development [76]. Growing evidence has also revealed that bZIP TFs have an important part in regulating plant’s response to biotic as well as abiotic stresses [76,77]. The Dof is a plant-specific gene family of TFs seen in green algae to higher plants which have characteristics of bifunctional binding with DNA as well as proteins to regulate the transcriptional machinery of plant cells [78,79]. Dof is also associated with gene regulation relating to seed maturation as well as germination, plant-hormone as well as light-dependent regulation, and plant’s resistance to biotic as well as abiotic stresses [78-80].

The patterns of expression of the WRKY TF family are related to the defense mechanism against necrotrophic pathogens, and biotrophic pathogens as well as also act as anti-microbial defense [81-83]. Besides, the expression of LIM gene families can be regulated by MYB, NAC, and WRKY TFs families at the time of different adverse conditions, as well as they, have a direct or indirect link to plant development, and stress response [82,84-86]. Regulation of defensive gene expression is carried out through interactive actions of Calmodulin with the various specific TFs for example MYB, NAC, and WRKY [87,88].

The G2-like (GOLDEN2-LIKE) proteins are members of the GARP (Golden2, ARR-B as well as Psr1) domain superfamily of TFs [89,90]. The G2-like TF family plays significant roles in forming and developing chloroplasts [91-95] as well as has been related to various defense mechanisms in organisms, including biotic, and abiotic stresses [96-100]. MIKC_MADS TFs family carry out a great role in the development of flowers as well as fruit [101]. The basic helix-loop-helix TF family carries out significant roles in plant’s stress tolerance, besides their great activities in reproduction, for example in the development of flower as well as fruit [102] and secondary metabolites biosynthesis, e.g., anthocyanin [103]. The analysis of the regulatory network precisely revealed that the predicted SbLIM genes and their associated TFs in *S. sorghum* will show a broad range of expression patterns which may be obtained by a thorough study of these genes in the future.

### CAREs analysis of LIM genes in sorghum

**Fig. 10.**
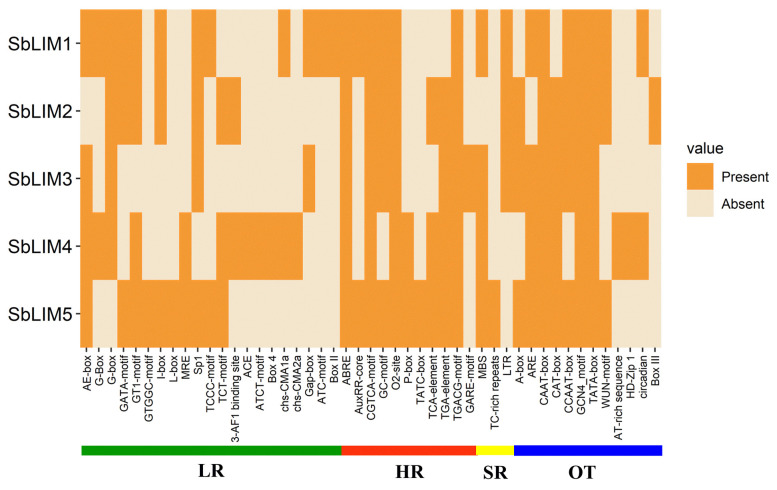
The cis-acting regulatory elements (CAREs) in the upstream promoter region of predicted SbLIM genes, respectively. The deep color represents the presence of that element with the corresponding genes. LR, light-responsive; HR, hormone-responsive; SR, stress-responsive; OT, other function.

The CAREs are composed of non-coding short DNA sequences ranging from 5 to 20 bp in length which are normally present in the promoter regions of a gene [104,105]. They contain binding sites for TFs as well as other various regulatory elements for triggering the transcription of genes [105]. In plants, CAREs provide defensive functions against various biotic, and abiotic stresses [104] and play an important role in developmental as well as physiological activities by regulating gene expression [105]. The CAREs analysis was performed to search for functional variations of motifs relating to the promoter region of the proposed LIM genes in *S. bicolor*. The database PlantCARE provided the required information about the motifs as well as their functions related to the genes. This analysis identified a total of 47 functional motifs relating to light-responsive (LR), hormone-responsive (HR), SR, and other functions (OT) (Fig. 10, Supplementary Table 5). We found that the maximum number (21 motifs) of motifs were LR, vastly located in the promoter region of all SbLIM genes. Among LR motifs AE-box, G-box, GATA-motif, GT1-motif, I-box, Sp1, and TCT-motif were common in most of the SbLIM genes in sorghum. Photosynthesis highly relies on the response of light which typically occurs in the leaves of every plant. It is also a crucial physiological factor in sorghum like other plants which is ultimately related to various aspects of increasing the quality and productivity of grain [106]. An enhanced photosynthesis rate can use solar radiation appropriately resulting in reduced flowering time as signals of flowering are generated in leaves [107]. Therefore, our identified LR-associated CAREs are supposed to have direct connections to increased photosynthesis rates in sorghum leaves.

PGRs play important regulatory roles coordinately or individually in plant growth as well as different development actions [108,109]. PGRs have a significant biological role in the germination of seeds and growth, development as well as metabolic activities in plants [104,110,111]. Eleven CAREs related to significant plants HR were recognized in this study. Among the HR motifs, abscisic acid responsiveness, CGTCA-motif (MeJA-responsiveness), GC-motif (associated in anoxic specific inducibility), O2-site (zein metabolism regulation), TCA-element (salicylic acid responsiveness), TGA-element (auxin responsiveness) and TGACG-motif (MeJA-responsiveness) were shared by most of the SbLIM genes. HR motifs prediction suggests that they have very significant biological functions in sorghum. MBS (MYB binding site) related to drought inducibility [112], TC-rich repeats associated with defense as well as stress responsiveness [113], and long terminal repeat elements involved in response to low-temperature were commonly observed as SR CAREs among all predicted SbLIM genes of *S. bicolor*.

There were also 12 other important CAREs found and designated as other functions (OT). A-box, ARE (essential for the anaerobic induction), CAAT-box (a common element in promoter as well as enhancer regions), CAT-box (involved in meristem expression), CCAAT-box (MYBHv1 binding site), GCN4_motif (associated with endosperm expression), TATA-box (core promoter element about –30 of transcription start), and WUN-motif (wound-responsive element) were considered as other CAREs shared by the maximum number of predicted SbLIM genes of sorghum which was consistent with the previous studies [40,114,115]. In addition, 35 CAREs with unknown functions were also identified in our study (Supplementary Table 5). Collectively, CAREs distributed among putative LIM gene family in sorghum will give important information about their roles in growth, development as well as defense against various biotic and abiotic stresses.

In the present study, in total five LIM genes were identified in the sorghum genome. Moreover, *in silico* characterization was performed using the integrated bioinformatics approaches. Phylogenetic tree analysis demonstrated that identified SbLIM genes were classified into four individual subfamilies, named αLIM, βLIM, δLIM, and γLIM. Configuration of the conserved domain, motifs as well as gene structures exhibited the highest similarity compared with the Arabidopsis LIM gene family. Besides, analysis of the GO of predicted SbLIM genes revealed that they are mainly involved in actin cytoskeletal organization and metal ion binding. In this study, we discovered a network of regulatory relationships between TFs as well as predicted LIM genes. Possible TFs and CAREs associated with plant growth, development, and gene regulation related to SbLIM genes were recognized. Therefore, these findings will provide a solid ground for further functional study of LIM genes in *S. bicolor* for clarifying their regulatory functions in plant growth, and development as well as biotic and abiotic stress resistance.

## Figures and Tables

**Table 1.** The physicochemical properties of *Sorghum bicolor* LIM gene family members

Sl No.	Gene	Accession No.	Chromosomal location	ORF (bp)	Gene length (bp)	Protein			
						Molecular weight (kD)	Protein length (aa)	pI	GRAVY
1	SbLIM1	Sobic.001G426200.1	Chr01:70577409..70579551	594	2,142	21.802	198	8.98	–0.554
2	SbLIM2	Sobic.004G303700.1	Chr04:64293407..64295083	603	1,676	21.615	201	8.38	–0.406
3	SbLIM3	Sobic.006G159000.1	Chr06:51718518..51720006	612	1,488	22.297	204	7.48	–0.421
4	SbLIM4	Sobic.008G110200.1	Chr08:51315200..51317365	585	2,165	21.889	195	8.63	–0.629
5	SbLIM5	Sobic.010G097600.1	Chr10:8834292..8842412	4,428	8,120	158.98	1,476	4.63	–0.976

ORF, open reading frame; GRAVY, grand average hydrophilicity.

## Supplementary Materials

Supplementary Table. 1. The details GO analysis of the identified five *Sorghum bicolor* genes was performed using the online tool Plant Transcription Factor Database (PlantTFDB, http://planttfdb.cbi.pku.edu.cn/).

Supplementary Table 2. Identified in a total of 334 transcription factors associated with the regulation of identified five SbLIM genes in *Sorghum bicolor* genome

Supplementary Table 3. Identified 34 transcriptions family and 136 associated with the regulation of identified five SbLIM genes in *Sorghum bicolor* genome

Supplementary Table 4. Identified 136 unique transcription factors (TFs) associated with the regulation of identified five SbLIM genes in *Sorghum bicolor* genome

Supplementary Table 5. The predicted cis-acting regulatory elements of the upstream promoter region (2.0 kb genomic sequences) of LIM gene families in *Sorghum bicolor*

Supplementary Fig. 1. Full-length genomic sequences of LIM gene families of *Arabidopsis thaliana* (Doc).

Supplementary Fig. 2. Full-length coding sequences of LIM gene families of *Arabidopsis thaliana* (Doc).

Supplementary Fig. 3. Full-length peptide sequences of LIM gene families of *Arabidopsis thaliana* (Doc).

Supplementary Fig. 4. Full-length genomic sequences of LIM gene families of *Arabidopsis thaliana* with 2,000 bp upstream region (Doc).

Supplementary Fig. 5. Full-length genomic sequences of LIM gene families of *Sorghum bicolor* (Doc).

Supplementary Fig. 6. Full-length coding sequences of LIM gene families of *Sorghum bicolor* (Doc).

Supplementary Fig. 7. Full-length peptide sequences of LIM gene families of *Sorghum bicolor* (Doc).

Supplementary Fig. 8. Full-length genomic sequences of LIM gene families of *Sorghum bicolor* with 2,000 bp upstream region (Doc).
