# Supplementary material for: A genome‑wide approach to the systematic and comprehensive analysis of LIM gene family in sorghum (Sorghum bicolor L.)
Source: Genomics Inform. 2023 Sep 27;21(3):e36. doi: 10.5808/gi.23007 (PMC10584642; doi:10.5808/gi.23007)
Supplement: Supplementary Table. 1. — The details GO analysis of the identified five Sorghum bicolor genes was performed using the online tool Plant Transcription Factor Database (PlantTFDB, http://planttfdb.cbi.pku.edu.cn/). [file gi-23007-Supplementary-Table-1.pdf]

Supplemental Table 1. The details GO analysis of the identified five *Sorghum bicolor* genes was performed using the online tool Plant Transcription Factor Database (PlantTFDB, http://planttfdb.cbi.pku.edu.cn//).

ID	SbLIM1	SbLIM2	SbLIM3	SbLIM4	SbLIM5
GO:0043167	Present	Present	Present	Present	Present
GO:0071840	Present	Present	Absent	Present	Absent
GO:0016043	Present	Present	Absent	Present	Absent
GO:0006996	Present	Present	Absent	Present	Absent
GO:0043169	Present	Present	Present	Present	Present
GO:0044085	Present	Present	Absent	Present	Absent
GO:0046872	Present	Present	Present	Present	Present
GO:0043933	Present	Present	Absent	Present	Absent
GO:0022607	Present	Present	Absent	Present	Absent
GO:1902589	Present	Present	Absent	Present	Absent
GO:0046914	Present	Present	Present	Present	Present
GO:0044877	Present	Present	Absent	Present	Absent
GO:0071822	Present	Present	Absent	Present	Absent
GO:0008092	Present	Present	Absent	Present	Absent
GO:0008270	Present	Present	Present	Present	Present
GO:0032403	Present	Present	Absent	Present	Absent
GO:0007010	Present	Present	Absent	Present	Absent
GO:0003779	Present	Present	Absent	Present	Absent
GO:0030029	Present	Present	Absent	Present	Absent
GO:0030036	Present	Present	Absent	Present	Absent
GO:0007015	Present	Present	Absent	Present	Absent
GO:0051015	Present	Present	Absent	Present	Absent
GO:0051017	Present	Present	Absent	Present	Absent
GO:0061572	Present	Present	Absent	Present	Absent
