# Supplementary material for: A genome‑wide approach to the systematic and comprehensive analysis of LIM gene family in sorghum (Sorghum bicolor L.)
Source: Genomics Inform. 2023 Sep 27;21(3):e36. doi: 10.5808/gi.23007 (PMC10584642; doi:10.5808/gi.23007)
Supplement: Supplementary Table 2. — Identified in a total of 334 transcription factors associated with the regulation of identified five SbLIM genes in Sorghum bicolor genome [file gi-23007-Supplementary-Table-2.pdf]

Supplementary Table 2. Identified in a total of 334 transcription factors associated with the regulation of identified five SbLIM genes in *Sorghum bicolor* genome

TF	Family	Gene name	p-value
Sobic.003G058200	ERF	SbLIM1	4.67E-07
Sobic.005G087600	ERF	SbLIM1	4.66E-06
Sobic.004G295500	ERF	SbLIM1	5.96E-06
Sobic.001G486800	ERF	SbLIM1	8.30E-06
Sobic.010G063900	ERF	SbLIM1	1.58E-05
Sobic.004G331300	ERF	SbLIM1	1.58E-05
Sobic.003G028000	ERF	SbLIM1	1.75E-05
Sobic.006G059800	ERF	SbLIM1	1.98E-05
Sobic.006G156100	ERF	SbLIM1	2.11E-05
Sobic.007G162700	ERF	SbLIM1	2.19E-05
Sobic.004G310600	ERF	SbLIM1	2.32E-05
Sobic.009G103800	ERF	SbLIM1	2.96E-05
Sobic.002G071600	ERF	SbLIM1	3.75E-05
Sobic.004G319700	ERF	SbLIM1	4.68E-05
Sobic.002G411000	ERF	SbLIM1	4.96E-05
Sobic.004G159500	ERF	SbLIM1	5.13E-05
Sobic.006G168000	ERF	SbLIM1	5.99E-05
Sobic.006G184700	ERF	SbLIM1	6.87E-05
Sobic.009G184300	ERF	SbLIM1	7.66E-05
Sobic.003G012800	ERF	SbLIM1	8.39E-05
Sobic.006G156100	ERF	SbLIM2	2.59E-06
Sobic.001G002500	ERF	SbLIM2	4.15E-06
Sobic.003G012800	ERF	SbLIM2	4.51E-06
Sobic.009G103800	ERF	SbLIM2	4.76E-06
Sobic.003G058200	ERF	SbLIM2	5.65E-06
Sobic.005G087600	ERF	SbLIM2	7.36E-06
Sobic.010G063900	ERF	SbLIM2	8.26E-06
Sobic.004G331300	ERF	SbLIM2	8.26E-06
Sobic.006G167800	ERF	SbLIM2	1.12E-05
Sobic.009G233100	ERF	SbLIM2	1.13E-05
Sobic.004G310600	ERF	SbLIM2	1.36E-05
Sobic.002G069166	ERF	SbLIM2	1.38E-05
Sobic.006G059800	ERF	SbLIM2	1.46E-05
Sobic.009G184300	ERF	SbLIM2	1.69E-05
Sobic.004G295500	ERF	SbLIM2	2.21E-05
Sobic.010G071700	ERF	SbLIM2	2.48E-05
Sobic.002G411000	ERF	SbLIM2	3.66E-05
Sobic.002G071600	ERF	SbLIM2	3.79E-05
Sobic.004G159500	ERF	SbLIM2	3.81E-05

Sobic.004G073100	ERF	SbLIM2	3.82E-05
Sobic.004G319700	ERF	SbLIM2	3.95E-05
Sobic.006G184700	ERF	SbLIM2	4.15E-05
Sobic.001G486800	ERF	SbLIM2	4.91E-05
Sobic.002G375400	ERF	SbLIM2	5.06E-05
Sobic.006G168000	ERF	SbLIM2	5.23E-05
Sobic.003G028000	ERF	SbLIM2	5.89E-05
Sobic.007G162700	ERF	SbLIM2	6.26E-05
Sobic.003G058200	ERF	SbLIM3	6.83E-07
Sobic.005G087600	ERF	SbLIM3	4.47E-06
Sobic.003G012800	ERF	SbLIM3	9.19E-06
Sobic.007G162700	ERF	SbLIM3	9.96E-06
Sobic.010G063900	ERF	SbLIM3	1.77E-05
Sobic.004G331300	ERF	SbLIM3	1.77E-05
Sobic.009G103800	ERF	SbLIM3	1.82E-05
Sobic.006G167800	ERF	SbLIM3	5.12E-05
Sobic.006G156100	ERF	SbLIM3	5.35E-05
Sobic.004G319700	ERF	SbLIM3	5.58E-05
Sobic.004G159500	ERF	SbLIM3	6.35E-05
Sobic.006G168000	ERF	SbLIM3	7.29E-05
Sobic.006G184700	ERF	SbLIM3	8.32E-05
Sobic.006G059800	ERF	SbLIM3	9.97E-05
Sobic.004G319700	ERF	SbLIM4	4.38E-06
Sobic.004G310600	ERF	SbLIM4	5.61E-06
Sobic.006G156100	ERF	SbLIM4	5.86E-06
Sobic.004G295500	ERF	SbLIM4	6.10E-06
Sobic.006G184700	ERF	SbLIM4	7.03E-06
Sobic.003G028000	ERF	SbLIM4	1.01E-05
Sobic.001G002500	ERF	SbLIM4	1.28E-05
Sobic.010G052600	ERF	SbLIM4	1.37E-05
Sobic.006G240500	ERF	SbLIM4	1.37E-05
Sobic.006G167800	ERF	SbLIM4	1.44E-05
Sobic.007G162700	ERF	SbLIM4	1.57E-05
Sobic.003G012800	ERF	SbLIM4	2.25E-05
Sobic.010G063900	ERF	SbLIM4	2.62E-05
Sobic.004G331300	ERF	SbLIM4	2.62E-05
Sobic.003G442100	ERF	SbLIM4	3.43E-05
Sobic.006G059800	ERF	SbLIM4	3.87E-05
Sobic.003G058200	ERF	SbLIM4	3.99E-05
Sobic.004G283201	ERF	SbLIM4	4.11E-05
Sobic.005G087600	ERF	SbLIM4	4.30E-05
Sobic.006G168000	ERF	SbLIM4	4.40E-05
Sobic.001G486800	ERF	SbLIM4	4.77E-05

Sobic.006G184800	ERF	SbLIM4	5.02E-05
Sobic.009G233100	ERF	SbLIM4	5.33E-05
Sobic.002G225700	ERF	SbLIM4	6.41E-05
Sobic.009G103800	ERF	SbLIM4	6.56E-05
Sobic.010G071700	ERF	SbLIM4	9.32E-05
Sobic.006G059800	ERF	SbLIM5	2.20E-07
Sobic.003G012800	ERF	SbLIM5	4.84E-07
Sobic.006G156100	ERF	SbLIM5	8.76E-07
Sobic.004G310600	ERF	SbLIM5	1.48E-06
Sobic.006G167800	ERF	SbLIM5	1.50E-06
Sobic.010G063900	ERF	SbLIM5	1.70E-06
Sobic.004G331300	ERF	SbLIM5	1.70E-06
Sobic.002G071600	ERF	SbLIM5	1.83E-06
Sobic.006G168000	ERF	SbLIM5	1.98E-06
Sobic.005G087600	ERF	SbLIM5	2.04E-06
Sobic.010G071700	ERF	SbLIM5	2.16E-06
Sobic.001G486800	ERF	SbLIM5	2.59E-06
Sobic.003G058200	ERF	SbLIM5	3.29E-06
Sobic.009G233100	ERF	SbLIM5	5.90E-06
Sobic.006G184800	ERF	SbLIM5	6.38E-06
Sobic.002G411000	ERF	SbLIM5	7.17E-06
Sobic.004G073100	ERF	SbLIM5	7.23E-06
Sobic.004G159500	ERF	SbLIM5	1.02E-05
Sobic.006G184700	ERF	SbLIM5	1.25E-05
Sobic.002G069166	ERF	SbLIM5	1.39E-05
Sobic.004G283201	ERF	SbLIM5	1.44E-05
Sobic.009G103800	ERF	SbLIM5	1.47E-05
Sobic.007G162700	ERF	SbLIM5	1.54E-05
Sobic.004G295500	ERF	SbLIM5	2.05E-05
Sobic.001G002500	ERF	SbLIM5	2.28E-05
Sobic.003G028000	ERF	SbLIM5	2.36E-05
Sobic.004G319700	ERF	SbLIM5	2.70E-05
Sobic.003G442100	ERF	SbLIM5	2.89E-05
Sobic.010G052600	ERF	SbLIM5	4.03E-05
Sobic.006G240500	ERF	SbLIM5	4.03E-05
Sobic.009G184300	ERF	SbLIM5	4.15E-05
Sobic.002G375400	ERF	SbLIM5	5.06E-05
Sobic.002G225700	ERF	SbLIM5	6.37E-05
Sobic.004G219800	MYB	SbLIM1	3.12E-06
Sobic.006G199800	MYB	SbLIM1	4.71E-06
Sobic.004G231700	MYB	SbLIM1	7.95E-06
Sobic.009G157500	MYB	SbLIM1	1.18E-05
Sobic.008G112200	MYB	SbLIM1	2.12E-05

Sobic.009G036500	MYB	SbLIM1	2.13E-05
Sobic.008G020300	MYB	SbLIM1	2.41E-05
Sobic.002G308400	MYB	SbLIM1	2.74E-05
Sobic.007G132600	MYB	SbLIM1	3.58E-05
Sobic.006G266600	MYB	SbLIM1	6.49E-05
Sobic.002G279100	MYB	SbLIM1	6.56E-05
Sobic.003G409900	MYB	SbLIM1	6.72E-05
Sobic.006G115200	MYB	SbLIM1	7.89E-05
Sobic.003G087600	MYB	SbLIM1	7.92E-05
Sobic.008G020300	MYB	SbLIM2	1.75E-06
Sobic.002G201000	MYB	SbLIM2	1.71E-05
Sobic.009G036500	MYB	SbLIM2	3.28E-05
Sobic.008G050500	MYB	SbLIM2	3.51E-05
Sobic.003G409900	MYB	SbLIM2	6.15E-05
Sobic.008G050500	MYB	SbLIM3	3.85E-05
Sobic.009G036500	MYB	SbLIM4	1.22E-08
Sobic.006G199800	MYB	SbLIM4	6.58E-08
Sobic.004G219800	MYB	SbLIM4	1.49E-07
Sobic.002G201000	MYB	SbLIM4	1.10E-06
Sobic.002G308400	MYB	SbLIM4	1.41E-06
Sobic.004G231700	MYB	SbLIM4	1.45E-05
Sobic.004G070900	MYB	SbLIM4	2.98E-05
Sobic.008G020300	MYB	SbLIM4	5.10E-05
Sobic.007G132600	MYB	SbLIM4	5.56E-05
Sobic.009G157500	MYB	SbLIM4	6.46E-05
Sobic.009G036500	MYB	SbLIM5	1.75E-07
Sobic.002G308400	MYB	SbLIM5	1.18E-06
Sobic.006G199800	MYB	SbLIM5	1.44E-06
Sobic.004G219800	MYB	SbLIM5	1.90E-06
Sobic.008G020300	MYB	SbLIM5	3.87E-06
Sobic.002G201000	MYB	SbLIM5	8.23E-06
Sobic.004G231700	MYB	SbLIM5	9.19E-06
Sobic.003G331100	MYB	SbLIM5	3.91E-05
Sobic.007G132600	MYB	SbLIM5	5.31E-05
Sobic.002G141300	MYB	SbLIM5	5.44E-05
Sobic.003G409900	MYB	SbLIM5	6.05E-05
Sobic.008G112200	MYB	SbLIM5	6.40E-05
Sobic.006G266600	MYB	SbLIM5	6.66E-05
Sobic.009G157500	MYB	SbLIM5	7.18E-05
Sobic.003G087600	MYB	SbLIM5	8.22E-05
Sobic.008G133700	MYB	SbLIM5	9.07E-05
Sobic.001G242900	C2H2	SbLIM1	1.03E-06
Sobic.009G024400	C2H2	SbLIM1	5.09E-06

Sobic.008G014801	C2H2	SbLIM1	5.09E-06
Sobic.007G173200	C2H2	SbLIM1	6.13E-06
Sobic.003G194600	C2H2	SbLIM1	8.29E-06
Sobic.001G469100	C2H2	SbLIM1	1.60E-05
Sobic.003G394900	C2H2	SbLIM1	2.54E-05
Sobic.007G166000	C2H2	SbLIM1	2.85E-05
Sobic.002G359300	C2H2	SbLIM1	7.61E-05
Sobic.002G345300	C2H2	SbLIM1	8.96E-05
Sobic.009G024400	C2H2	SbLIM2	1.14E-06
Sobic.008G014801	C2H2	SbLIM2	1.14E-06
Sobic.007G173200	C2H2	SbLIM2	4.71E-06
Sobic.001G242900	C2H2	SbLIM2	5.60E-06
Sobic.003G194600	C2H2	SbLIM2	9.73E-06
Sobic.001G469100	C2H2	SbLIM2	1.37E-05
Sobic.003G370700	C2H2	SbLIM2	8.99E-05
Sobic.009G024400	C2H2	SbLIM3	1.32E-06
Sobic.008G014801	C2H2	SbLIM3	1.32E-06
Sobic.003G394900	C2H2	SbLIM3	6.34E-05
Sobic.009G024400	C2H2	SbLIM4	5.48E-06
Sobic.008G014801	C2H2	SbLIM4	5.48E-06
Sobic.003G394900	C2H2	SbLIM4	9.95E-06
Sobic.004G351700	C2H2	SbLIM4	2.48E-05
Sobic.001G242900	C2H2	SbLIM4	6.30E-05
Sobic.001G079500	C2H2	SbLIM4	8.80E-05
Sobic.009G024400	C2H2	SbLIM5	2.46E-07
Sobic.008G014801	C2H2	SbLIM5	2.46E-07
Sobic.001G079500	C2H2	SbLIM5	9.15E-06
Sobic.004G351700	C2H2	SbLIM5	1.96E-05
Sobic.007G173200	C2H2	SbLIM5	2.57E-05
Sobic.003G370700	C2H2	SbLIM5	4.34E-05
Sobic.003G394900	C2H2	SbLIM5	4.65E-05
Sobic.001G469100	C2H2	SbLIM5	4.65E-05
Sobic.002G359300	C2H2	SbLIM5	7.61E-05
Sobic.003G194600	C2H2	SbLIM5	7.87E-05
Sobic.003G329500	bZIP	SbLIM1	1.97E-05
Sobic.008G037900	bZIP	SbLIM1	2.87E-05
Sobic.010G194900	bZIP	SbLIM1	4.63E-05
Sobic.002G247300	bZIP	SbLIM1	5.23E-05
Sobic.004G085600	bZIP	SbLIM2	1.62E-05
Sobic.008G037900	bZIP	SbLIM2	5.35E-05
Sobic.006G233500	bZIP	SbLIM2	5.51E-05
Sobic.003G329500	bZIP	SbLIM2	6.25E-05
Sobic.002G247300	bZIP	SbLIM2	9.30E-05

Sobic.002G418100	bZIP	SbLIM3	7.39E-05
Sobic.002G247300	bZIP	SbLIM3	8.31E-05
Sobic.008G037900	bZIP	SbLIM4	1.43E-05
Sobic.008G046600	bZIP	SbLIM4	4.36E-05
Sobic.003G329500	bZIP	SbLIM4	5.09E-05
Sobic.002G418100	bZIP	SbLIM4	5.91E-05
Sobic.008G046600	bZIP	SbLIM5	1.42E-05
Sobic.002G418100	bZIP	SbLIM5	3.27E-05
Sobic.003G363400	bZIP	SbLIM5	3.96E-05
Sobic.004G031100	bZIP	SbLIM5	4.11E-05
Sobic.006G233500	bZIP	SbLIM5	4.50E-05
Sobic.007G155900	bZIP	SbLIM5	4.74E-05
Sobic.010G194900	bZIP	SbLIM5	5.19E-05
Sobic.009G237600	bZIP	SbLIM5	5.27E-05
Sobic.004G085600	bZIP	SbLIM5	5.36E-05
Sobic.008G083200	bZIP	SbLIM5	5.36E-05
Sobic.009G178800	bZIP	SbLIM5	5.42E-05
Sobic.003G329500	bZIP	SbLIM5	6.78E-05
Sobic.009G152100	bZIP	SbLIM5	7.83E-05
Sobic.009G014400	Dof	SbLIM1	1.25E-07
Sobic.001G420300	Dof	SbLIM1	1.25E-07
Sobic.006G267900	Dof	SbLIM1	2.67E-07
Sobic.004G266200	Dof	SbLIM1	3.80E-07
Sobic.001G034300	Dof	SbLIM1	2.15E-06
Sobic.008G136100	Dof	SbLIM1	2.18E-06
Sobic.004G254000	Dof	SbLIM1	8.54E-06
Sobic.009G014400	Dof	SbLIM3	1.49E-06
Sobic.001G420300	Dof	SbLIM3	1.49E-06
Sobic.006G267900	Dof	SbLIM3	1.69E-06
Sobic.004G266200	Dof	SbLIM3	9.50E-06
Sobic.008G136100	Dof	SbLIM3	1.78E-05
Sobic.004G266200	Dof	SbLIM4	1.78E-07
Sobic.009G014400	Dof	SbLIM4	1.62E-06
Sobic.001G420300	Dof	SbLIM4	1.62E-06
Sobic.006G267900	Dof	SbLIM4	7.86E-06
Sobic.008G136100	Dof	SbLIM4	2.99E-05
Sobic.001G034300	Dof	SbLIM4	4.54E-05
Sobic.004G254000	Dof	SbLIM4	7.26E-05
Sobic.003G367100	Dof	SbLIM5	1.10E-06
Sobic.006G267900	Dof	SbLIM5	1.90E-06
Sobic.008G136100	Dof	SbLIM5	2.83E-06
Sobic.004G254000	Dof	SbLIM5	3.00E-06
Sobic.001G034300	Dof	SbLIM5	3.21E-06

Sobic.004G266200	Dof	SbLIM5	4.28E-06
Sobic.009G014400	Dof	SbLIM5	1.00E-05
Sobic.001G420300	Dof	SbLIM5	1.00E-05
Sobic.003G121400	Dof	SbLIM5	1.64E-05
Sobic.002G251700	NAC	SbLIM1	8.08E-06
Sobic.002G253000	NAC	SbLIM1	3.63E-05
Sobic.007G209200	NAC	SbLIM1	4.16E-05
Sobic.002G290800	NAC	SbLIM1	4.69E-05
Sobic.006G279400	NAC	SbLIM1	6.03E-05
Sobic.008G071400	NAC	SbLIM1	6.24E-05
Sobic.001G071000	NAC	SbLIM1	6.69E-05
Sobic.004G237900	NAC	SbLIM2	2.65E-05
Sobic.002G342100	NAC	SbLIM2	4.72E-05
Sobic.008G071400	NAC	SbLIM2	5.19E-05
Sobic.002G253000	NAC	SbLIM2	7.89E-05
Sobic.001G522700	NAC	SbLIM3	1.44E-06
Sobic.003G035100	NAC	SbLIM3	7.72E-06
Sobic.003G379700	NAC	SbLIM3	1.38E-05
Sobic.003G251800	NAC	SbLIM3	1.41E-05
Sobic.007G209200	NAC	SbLIM3	2.63E-05
Sobic.002G290800	NAC	SbLIM3	2.79E-05
Sobic.007G166100	NAC	SbLIM3	4.46E-05
Sobic.001G290400	NAC	SbLIM3	4.64E-05
Sobic.009G143700	NAC	SbLIM3	7.36E-05
Sobic.008G071400	NAC	SbLIM3	9.20E-05
Sobic.007G166100	NAC	SbLIM5	1.38E-05
Sobic.006G279400	NAC	SbLIM5	3.00E-05
Sobic.009G143700	NAC	SbLIM5	3.33E-05
Sobic.006G092100	NAC	SbLIM5	6.77E-05
Sobic.002G251700	NAC	SbLIM5	8.76E-05
Sobic.003G379700	NAC	SbLIM5	9.33E-05
Sobic.010G155100	NAC	SbLIM5	9.49E-05
Sobic.010G254300	G2-like	SbLIM1	6.65E-05
Sobic.002G161800	G2-like	SbLIM1	7.28E-05
Sobic.004G273000	G2-like	SbLIM2	5.20E-05
Sobic.001G386700	G2-like	SbLIM2	5.49E-05
Sobic.008G036900	G2-like	SbLIM2	6.10E-05
Sobic.003G046800	G2-like	SbLIM2	6.27E-05
Sobic.004G270600	G2-like	SbLIM2	6.59E-05
Sobic.008G004200	G2-like	SbLIM2	8.71E-05
Sobic.010G254300	G2-like	SbLIM3	4.67E-05
Sobic.003G046800	G2-like	SbLIM3	8.19E-05
Sobic.008G004200	G2-like	SbLIM4	1.52E-05

Sobic.008G036900	G2-like	SbLIM4	1.60E-05
Sobic.004G273000	G2-like	SbLIM4	3.32E-05
Sobic.004G270600	G2-like	SbLIM4	3.32E-05
Sobic.001G386700	G2-like	SbLIM4	4.20E-05
Sobic.002G161800	G2-like	SbLIM4	4.27E-05
Sobic.010G096300	G2-like	SbLIM4	8.50E-05
Sobic.004G273000	G2-like	SbLIM5	1.51E-05
Sobic.008G004200	G2-like	SbLIM5	1.87E-05
Sobic.010G254300	G2-like	SbLIM5	2.14E-05
Sobic.010G096300	G2-like	SbLIM5	2.51E-05
Sobic.001G386700	G2-like	SbLIM5	2.55E-05
Sobic.003G046800	G2-like	SbLIM5	4.22E-05
Sobic.004G270600	G2-like	SbLIM5	4.66E-05
Sobic.008G036900	G2-like	SbLIM5	4.71E-05
Sobic.002G161800	G2-like	SbLIM5	8.37E-05
Sobic.002G202700	WRKY	SbLIM1	9.15E-05
Sobic.008G060300	WRKY	SbLIM2	3.30E-05
Sobic.004G117600	WRKY	SbLIM2	3.66E-05
Sobic.004G271800	WRKY	SbLIM2	3.71E-05
Sobic.003G337800	WRKY	SbLIM2	4.37E-05
Sobic.009G234900	WRKY	SbLIM2	4.53E-05
Sobic.003G341100	WRKY	SbLIM2	4.74E-05
Sobic.006G206000	WRKY	SbLIM2	4.97E-05
Sobic.002G355000	WRKY	SbLIM2	5.10E-05
Sobic.003G248400	WRKY	SbLIM2	5.28E-05
Sobic.009G092100	WRKY	SbLIM2	5.62E-05
Sobic.003G296300	WRKY	SbLIM2	5.84E-05
Sobic.008G107500	WRKY	SbLIM2	6.10E-05
Sobic.003G287200	WRKY	SbLIM2	8.39E-05
Sobic.002G174200	WRKY	SbLIM2	8.43E-05
Sobic.003G000600	WRKY	SbLIM2	9.40E-05
Sobic.005G117400	WRKY	SbLIM2	9.64E-05
Sobic.010G209200	WRKY	SbLIM2	9.66E-05
Sobic.004G298400	WRKY	SbLIM5	6.88E-05
Sobic.002G174200	WRKY	SbLIM5	7.35E-05
Sobic.003G337800	WRKY	SbLIM5	7.82E-05
Sobic.003G000600	WRKY	SbLIM5	9.36E-05
