# Supplementary material for: A genome‑wide approach to the systematic and comprehensive analysis of LIM gene family in sorghum (Sorghum bicolor L.)
Source: Genomics Inform. 2023 Sep 27;21(3):e36. doi: 10.5808/gi.23007 (PMC10584642; doi:10.5808/gi.23007)
Supplement: Supplementary Table 3. — Identified 34 transcriptions family and 136 associated with the regulation of identified five SbLIM genes in Sorghum bicolor genome [file gi-23007-Supplementary-Table-3.pdf]

Supplementary Table 3. Identified 34 transcriptions family and 136 associated with the regulation of identified five SbLIM genes in *Sorghum bicolor* genome

**Family**	**SbLIM1**	**SbLIM2**	**SbLIM3**	**SbLIM4**	**SbLIM5**
ERF	20	27	14	26	33
MYB	14	5	1	10	16
C2H2	10	7	3	6	10
bZIP	4	5	2	4	13
Dof	7	0	5	7	9
NAC	7	4	10	0	7
G2-like	2	6	2	7	9
WRKY	1	17	0	0	4
MIKC_MADS	6	0	1	4	9
LBD	4	4	2	1	4
HD-ZIP	1	1	4	4	3
B3	2	2	3	2	3
SBP	5	1	1	0	5
GATA	4	1	0	3	3
ARF	0	1	0	6	3
MYB_related	3	0	1	2	4
TALE	3	1	0	3	3
Trihelix	2	2	1	1	3
BBR-BPC	2	0	2	2	2
bHLH	1	0	0	0	5
HSF	1	0	0	0	5
TCP	0	1	0	0	5
WOX	2	2	0	1	1
GRAS	1	1	1	1	1
AP2	0	1	1	1	1
CPP	1	1	0	0	2
YABBY	0	0	1	1	2
BES1	0	0	0	1	2
RAV	1	1	0	0	1
C3H	0	0	0	1	1
E2F/DP	1	1	0	0	0
EIL	0	0	0	0	2
ARR-B	0	0	0	0	1
LFY	0	0	0	1	0
