# Supplementary material for: A genome‑wide approach to the systematic and comprehensive analysis of LIM gene family in sorghum (Sorghum bicolor L.)
Source: Genomics Inform. 2023 Sep 27;21(3):e36. doi: 10.5808/gi.23007 (PMC10584642; doi:10.5808/gi.23007)
Supplement: Supplementary Table 4. — Identified 136 unique transcription factors (TFs) associated with the regulation of identified five SbLIM genes in Sorghum bicolor genome [file gi-23007-Supplementary-Table-4.pdf]

Supplementary Table 4. Identified 136 unique transcription factors (TFs) associated with the regulation of identified five SbLIM genes in *Sorghum bicolor* genome

TF	SbLIM1	SbLIM2	SbLIM3	SbLIM4	SbLIM5
Sobic.003G058200	Yes	Yes	Yes	Yes	Yes
Sobic.005G087600	Yes	Yes	Yes	Yes	Yes
Sobic.004G295500	Yes	Yes	No	Yes	Yes
Sobic.001G486800	Yes	Yes	No	Yes	Yes
Sobic.010G063900	Yes	Yes	Yes	Yes	Yes
Sobic.004G331300	Yes	Yes	Yes	Yes	Yes
Sobic.003G028000	Yes	Yes	No	Yes	Yes
Sobic.006G059800	Yes	Yes	Yes	Yes	Yes
Sobic.006G156100	Yes	Yes	Yes	Yes	Yes
Sobic.007G162700	Yes	Yes	Yes	Yes	Yes
Sobic.004G310600	Yes	Yes	No	Yes	Yes
Sobic.009G103800	Yes	Yes	Yes	Yes	Yes
Sobic.002G071600	Yes	Yes	No	No	Yes
Sobic.004G319700	Yes	Yes	Yes	Yes	Yes
Sobic.002G411000	Yes	Yes	No	No	Yes
Sobic.004G159500	Yes	Yes	Yes	No	Yes
Sobic.006G168000	Yes	Yes	Yes	Yes	Yes
Sobic.006G184700	Yes	Yes	Yes	Yes	Yes
Sobic.009G184300	Yes	Yes	No	No	Yes
Sobic.003G012800	Yes	Yes	Yes	Yes	Yes
Sobic.001G002500	No	Yes	No	Yes	Yes
Sobic.006G167800	No	Yes	Yes	Yes	Yes
Sobic.009G233100	No	Yes	No	Yes	Yes
Sobic.002G069166	No	Yes	No	No	Yes
Sobic.010G071700	No	Yes	No	Yes	Yes
Sobic.004G073100	No	Yes	No	No	Yes
Sobic.002G375400	No	Yes	No	No	Yes
Sobic.010G052600	No	No	No	Yes	Yes
Sobic.006G240500	No	No	No	Yes	Yes
Sobic.003G442100	No	No	No	Yes	Yes
Sobic.004G283201	No	No	No	Yes	Yes
Sobic.006G184800	No	No	No	Yes	Yes
Sobic.002G225700	No	No	No	Yes	Yes
Sobic.004G219800	Yes	No	No	Yes	Yes
Sobic.006G199800	Yes	No	No	Yes	Yes
Sobic.004G231700	Yes	No	No	Yes	Yes
Sobic.009G157500	Yes	No	No	Yes	Yes
Sobic.008G112200	Yes	No	No	No	Yes
Sobic.009G036500	Yes	Yes	No	Yes	Yes

Sobic.008G020300	Yes	Yes	No	Yes	Yes
Sobic.002G308400	Yes	No	No	Yes	Yes
Sobic.007G132600	Yes	No	No	Yes	Yes
Sobic.006G266600	Yes	No	No	No	Yes
Sobic.002G279100	Yes	No	No	No	No
Sobic.003G409900	Yes	Yes	No	No	Yes
Sobic.006G115200	Yes	No	No	No	No
Sobic.003G087600	Yes	No	No	No	Yes
Sobic.002G201000	No	Yes	No	Yes	Yes
Sobic.008G050500	No	Yes	Yes	No	No
Sobic.004G070900	No	No	No	Yes	No
Sobic.003G331100	No	No	No	No	Yes
Sobic.002G141300	No	No	No	No	Yes
Sobic.008G133700	No	No	No	No	Yes
Sobic.002G202700	Yes	No	No	No	No
Sobic.008G060300	No	Yes	No	No	No
Sobic.004G117600	No	Yes	No	No	No
Sobic.004G271800	No	Yes	No	No	No
Sobic.003G337800	No	Yes	No	No	Yes
Sobic.009G234900	No	Yes	No	No	No
Sobic.003G341100	No	Yes	No	No	No
Sobic.006G206000	No	Yes	No	No	No
Sobic.002G355000	No	Yes	No	No	No
Sobic.003G248400	No	Yes	No	No	No
Sobic.009G092100	No	Yes	No	No	No
Sobic.003G296300	No	Yes	No	No	No
Sobic.008G107500	No	Yes	No	No	No
Sobic.003G287200	No	Yes	No	No	No
Sobic.002G174200	No	Yes	No	No	Yes
Sobic.003G000600	No	Yes	No	No	Yes
Sobic.005G117400	No	Yes	No	No	No
Sobic.010G209200	No	Yes	No	No	No
Sobic.004G298400	No	No	No	No	Yes
Sobic.002G251700	Yes	No	No	No	Yes
Sobic.002G253000	Yes	Yes	No	No	No
Sobic.007G209200	Yes	No	Yes	No	No
Sobic.002G290800	Yes	No	Yes	No	No
Sobic.006G279400	Yes	No	No	No	Yes
Sobic.008G071400	Yes	Yes	Yes	No	No
Sobic.001G071000	Yes	No	No	No	No
Sobic.004G237900	No	Yes	No	No	No
Sobic.002G342100	No	Yes	No	No	No
Sobic.001G522700	No	No	Yes	No	No

Sobic.003G035100	No	No	Yes	No	No
Sobic.003G379700	No	No	Yes	No	Yes
Sobic.003G251800	No	No	Yes	No	No
Sobic.007G166100	No	No	Yes	No	Yes
Sobic.001G290400	No	No	Yes	No	No
Sobic.009G143700	No	No	Yes	No	Yes
Sobic.006G092100	No	No	No	No	Yes
Sobic.010G155100	No	No	No	No	Yes
Sobic.003G329500	Yes	Yes	No	Yes	Yes
Sobic.008G037900	Yes	Yes	No	Yes	No
Sobic.010G194900	Yes	No	No	No	Yes
Sobic.002G247300	Yes	Yes	Yes	No	No
Sobic.004G085600	No	Yes	No	No	Yes
Sobic.006G233500	No	Yes	No	No	Yes
Sobic.002G418100	No	No	Yes	Yes	Yes
Sobic.008G046600	No	No	No	Yes	Yes
Sobic.003G363400	No	No	No	No	Yes
Sobic.004G031100	No	No	No	No	Yes
Sobic.007G155900	No	No	No	No	Yes
Sobic.009G237600	No	No	No	No	Yes
Sobic.008G083200	No	No	No	No	Yes
Sobic.009G178800	No	No	No	No	Yes
Sobic.009G152100	No	No	No	No	Yes
Sobic.001G242900	Yes	Yes	No	Yes	No
Sobic.009G024400	Yes	Yes	Yes	Yes	Yes
Sobic.008G014801	Yes	Yes	Yes	Yes	Yes
Sobic.007G173200	Yes	Yes	No	No	Yes
Sobic.003G194600	Yes	Yes	No	No	Yes
Sobic.001G469100	Yes	Yes	No	No	Yes
Sobic.003G394900	Yes	No	Yes	Yes	Yes
Sobic.007G166000	Yes	No	No	No	No
Sobic.002G359300	Yes	No	No	No	Yes
Sobic.002G345300	Yes	No	No	No	No
Sobic.003G370700	No	Yes	No	No	Yes
Sobic.004G351700	No	No	No	Yes	Yes
Sobic.001G079500	No	No	No	Yes	Yes
Sobic.009G014400	Yes	No	Yes	Yes	Yes
Sobic.001G420300	Yes	No	Yes	Yes	Yes
Sobic.006G267900	Yes	No	Yes	Yes	Yes
Sobic.004G266200	Yes	No	Yes	Yes	Yes
Sobic.001G034300	Yes	No	No	Yes	Yes
Sobic.008G136100	Yes	No	Yes	Yes	Yes
Sobic.004G254000	Yes	No	No	Yes	Yes

Sobic.003G367100	No	No	No	No	Yes
Sobic.003G121400	No	No	No	No	Yes
Sobic.010G254300	Yes	No	Yes	No	Yes
Sobic.002G161800	Yes	No	No	Yes	Yes
Sobic.004G273000	No	Yes	No	Yes	Yes
Sobic.001G386700	No	Yes	No	Yes	Yes
Sobic.008G036900	No	Yes	No	Yes	Yes
Sobic.003G046800	No	Yes	Yes	No	Yes
Sobic.004G270600	No	Yes	No	Yes	Yes
Sobic.008G004200	No	Yes	No	Yes	Yes
Sobic.010G096300	No	No	No	Yes	Yes
