# Supplementary material for: A genome‑wide approach to the systematic and comprehensive analysis of LIM gene family in sorghum (Sorghum bicolor L.)
Source: Genomics Inform. 2023 Sep 27;21(3):e36. doi: 10.5808/gi.23007 (PMC10584642; doi:10.5808/gi.23007)
Supplement: Supplementary Table 5. — The predicted cis-acting regulatory elements of the upstream promoter region (2.0 kb genomic sequences) of LIM gene families in Sorghum bicolor [file gi-23007-Supplementary-Table-5.pdf]

Supplementary Table 5. The predicted cis-acting regulatory elements of the upstream promoter region (2.0 kb genomic sequences) of LIM gene families in *Sorghum bicolor*

Term	Function	Group	SbLIM1	SbLIM2	SbLIM3	SbLIM4	SbLIM5
AE-box	part of a module for light response	LR	Present	Absent	Present	Present	Present
G-Box	cis-acting regulatory element involved in light responsiveness		Present	Absent	Absent	Present	Absent
G-box	cis-acting regulatory element involved in light responsiveness		Present	Present	Present	Present	Absent
GATA-motif	part of a light responsive element		Present	Present	Absent	Absent	Present
GT1-motif	light responsive element		Present	Present	Absent	Present	Present
GTGGC-motif	part of a light responsive element		Absent	Absent	Absent	Absent	Present
I-box	part of a light responsive element		Present	Present	Absent	Absent	Present
L-box	part of a light responsive element		Absent	Absent	Absent	Absent	Present
MRE	MYB binding site involved in light responsiveness		Absent	Absent	Absent	Present	Present
Sp1	light responsive element		Present	Present	Present	Absent	Present
TCCC-motif	part of a light responsive element		Present	Absent	Absent	Absent	Present
TCT-motif	part of a light responsive element		Absent	Present	Absent	Present	Present
3-AF1 binding site	light responsive element		Absent	Present	Absent	Present	Absent
ACE	cis-acting element involved in light responsiveness		Absent	Absent	Absent	Present	Absent
ATCT-motif	part of a conserved DNA module involved in light responsiveness		Absent	Absent	Absent	Present	Absent
Box 4	part of a conserved DNA module involved in light responsiveness		Absent	Absent	Absent	Present	Absent
chs-CMA1a	part of a light responsive element		Present	Absent	Absent	Present	Absent
chs-CMA2a	part of a light responsive element		Absent	Absent	Absent	Present	Absent
Gap-box	part of a light responsive element		Present	Absent	Present	Absent	Absent
ATC-motif	part of a conserved DNA module involved in light responsiveness		Present	Absent	Absent	Absent	Absent
Box II	part of a light responsive element		Present	Absent	Absent	Absent	Absent

ABRE	cis-acting element involved in the abscisic acid responsiveness	HR	Present	Present	Present	Present	Present
AuxRR-core	cis-acting regulatory element involved in auxin responsiveness		Present	Absent	Absent	Absent	Present
CGTCA-motif	cis-acting regulatory element involved in the MeJA-responsiveness		Present	Present	Present	Present	Present
GC-motif	enhancer-like element involved in anoxic specific inducibility		Present	Present	Present	Absent	Present
O2-site	cis-acting regulatory element involved in zein metabolism regulation		Present	Present	Present	Present	Present
P-box	gibberellin-responsive element		Absent	Absent	Absent	Present	Present
TATC-box	cis-acting element involved in gibberellin-responsiveness		Absent	Absent	Absent	Absent	Present
TCA-element	cis-acting element involved in salicylic acid responsiveness		Absent	Present	Absent	Present	Present
TGA-element	auxin-responsive element		Absent	Present	Present	Present	Present
TGACG-motif	cis-acting regulatory element involved in the MeJA-responsiveness		Present	Present	Present	Present	Present
GARE-motif	gibberellin-responsive element		Absent	Absent	Present	Absent	Absent
MBS	MYB binding site involved in drought-inducibility	SR	Present	Absent	Present	Present	Present
TC-rich repeats	cis-acting element involved in defense and stress responsiveness		Absent	Absent	Absent	Absent	Present
LTR	cis-acting element involved in low-temperature responsiveness		Present	Present	Present	Absent	Absent
A-box	cis-acting regulatory element	OT	Absent	Present	Present	Absent	Present
ARE	cis-acting regulatory element essential for the anaerobic induction		Present	Absent	Present	Present	Present
CAAT-box	common cis-acting element in promoter and enhancer regions		Present	Present	Present	Present	Present
CAT-box	cis-acting regulatory element related to meristem expression		Absent	Present	Present	Present	Present

CCAAT-box	MYBHv1 binding site		Present	Present	Present	Absent	Present
GCN4_motif	cis-regulatory element involved in endosperm expression		Present	Present	Present	Present	Present
TATA-box	core promoter element around -30 of transcription start		Present	Present	Present	Present	Present
WUN-motif	wound-responsive element		Present	Present	Absent	Absent	Present
AT-rich sequence	element for maximal elicitor-mediated activation (2copies)		Absent	Absent	Absent	Present	Absent
HD-Zip 1	element involved in differentiation of the palisade mesophyll cells		Absent	Absent	Absent	Present	Absent
circadian	cis-acting regulatory element involved in circadian control		Present	Absent	Absent	Present	Absent
Box III	protein binding site		Absent	Present	Absent	Absent	Absent
AAGAA-motif	NA	Un	Present	Present	Present	Present	Present
AT~TATA-box	NA		Present	Present	Present	Present	Present
CCGTCC motif	NA		Absent	Present	Present	Absent	Present
CCGTCC-box	NA		Absent	Absent	Present	Absent	Present
DRE core	NA		Present	Present	Present	Present	Present
ERE	NA		Absent	Present	Present	Absent	Present
H-box	NA		Absent	Absent	Absent	Absent	Present
MYB	NA		Present	Present	Present	Present	Present
MYB recognition site	NA		Present	Absent	Present	Absent	Present
MYB-like sequence	NA		Present	Present	Present	Present	Present
MYC	NA		Present	Present	Present	Present	Present
Myb-binding site	NA		Present	Absent	Present	Absent	Present
STRE	NA		Present	Present	Present	Present	Present
TATA	NA		Present	Present	Present	Present	Present
TCA	NA		Present	Present	Present	Present	Present
Unnamed__1	NA		Present	Present	Present	Present	Present
Unnamed__2	NA		Absent	Present	Absent	Absent	Present

Unnamed__4	NA	Present	Present	Present	Present	Present
W box	NA	Present	Present	Absent	Present	Present
WRE3	NA	Present	Present	Present	Present	Present
as-1	NA	Present	Present	Present	Present	Present
box S	NA	Absent	Absent	Absent	Present	Present
CARE	NA	Absent	Absent	Absent	Present	Absent
CTAG-motif	NA	Absent	Absent	Absent	Present	Absent
Unnamed__6	NA	Present	Absent	Absent	Present	Absent
ABRE3a	NA	Present	Absent	Present	Absent	Absent
ABRE4	NA	Present	Present	Absent	Absent	Absent
DRE1	NA	Absent	Present	Present	Absent	Absent
W box	NA	Absent	Absent	Present	Absent	Absent
CCGTCC-box	NA	Absent	Present	Absent	Absent	Absent
E2Fb	NA	Present	Present	Absent	Absent	Absent
MYB recognition site	NA	Absent	Present	Absent	Absent	Absent
Unnamed__16	NA	Absent	Present	Absent	Absent	Absent
plant_AP-2-like	NA	Present	Present	Absent	Absent	Absent
JERE	NA	Present	Absent	Absent	Absent	Absent
