# Supplementary material for: A genome‑wide approach to the systematic and comprehensive analysis of LIM gene family in sorghum (Sorghum bicolor L.)
Source: Genomics Inform. 2023 Sep 27;21(3):e36. doi: 10.5808/gi.23007 (PMC10584642; doi:10.5808/gi.23007)
Supplement: Supplementary Fig. 1. — Full-length genomic sequences of LIM gene families of Arabidopsis thaliana (Doc). [file gi-23007-Supplementary-Fig-1.pdf]

Supplementary Fig. 1. Full-length genomic sequences of LIM gene families of *Arabidopsis thaliana* (Doc).

>AtWLIM1

AACAATAATTGACAATCTCTTTCTTTCATCTTCATCTTATTCTTCTTTATCTCTCTCTC

TCTCTCAAGACTATTGAGCTTCTTTCTTTTTTCCTGAGTCTTTTAGTCTGATCTCTTAC

AAGAATGGCGTTCGCAGGAACAACCCAGAAATGCATGGCATGTGACAAAACAGTTT

ATCTTGTCGACAAGTTAACCGCCGATAACCGGGTCTACCACAAAGCTTGTTTCCGAT

GTCACCATTGCAAAGGAACTCTCAAGGTTTGATCTTTGATTCTAGTTTGTGATGATTT

CTCATTAATCCAGAAGTAGATCTAGTAGATATATATGTAGATGAAGAAGTCCAATCA

ATTAAGTAGAAATCTTTGAATTTTTGGCTATGGAAACTGATTGGTCTAAAGATTTGA

CTGTTCTGTTTCTTCTTCTTCTTCTTCTTCTTCTTGATGAGTCAGCTTAGCAATTACAA

CTCCTTTGAAGGAGTTCTCTACTGCAGACCACATTTCGATCAAAACTTCAAGAGAAC

TGGAAGTCTTGAGAAAAGCTTCGAAGGTAAATTACCAAAAACAGATCAAAATGTGA

AAAGCTTTGTGAGTGGGGATTTCAAATTTGGAATTATCTATCTAAAGTTTGATTCAT

ATATATGTACAGGGACACCAAAGATTGGGAAACCTGATAGGCCTTTGGAGGGAGAG

GTACTTTCGGACAGCAGAACAAAACCCTTTTTCTGTTTTTGAGGTTCTTGAATTTTTT

TAACATTTAACATGAGAGAAGTTTAACAGAGACCTGCTGGAACCAAAGTTTCGAAT

ATGTTTGGTGGAACACGAGAGAAATGCGTTGGTTGCGACAAAACCGTGTATCCAATT

GAGAAGGTATCCAATCTCAAATCTTTCCTGATCAGAATCAAGTTGTGTCAAAGAAGA

TTGTTAGATTGTTTCCATAACGAATCTGATCTGTTCTTGGCTTAAACCAGAGCTGAAA

CATTGCTTATAATGTGGACTTTGTGTAGGTATCGGTGAATGGAACATTGTACCACAA

GAGCTGCTTCAAGTGTACACATGGAGGCTGCACGATAAGCCCTTCGAATTACATAGC

TCACGAGGGTAAGCTATATTGCAAGCATCATCATATTCAGCTGATCAAGGAGAAAG

GAAACTTGAGCCAGCTCGAAGGAGGAGGAGAGAATGCCGCCAAGGACAAAGTCGT

CGCTGCTTAAAGAAACAATTCAATCTCTCTGGGATGATTTTGACTATCGAAGAATCA

AAAGAGTCACTACTTCTCCATCGATCGATAACATATATTGGTTTTGTTTCTTCCTACG

TTTGTGTTTTTCTTTTTGGTCTTTAATATGTTTTTTTTTTTTTGGGACCTTTGTTCACTC

TTTTATTGGGTTTCTTGAAATGAAACTAGAGAAACAAAGAAATAATGGATGTTTGCT

TATGAGATATATATGTATTACGTTCATATGATATAAATTAATTTTCGTGAAGTTTAAG

CTTCAGC

>AtWLIM2a

ATTTTTTATCAATCGTGCTCCGTAATCATTACCTTTCTTCTTCATCTTCATCTTCACGC

AGCGACTTCTTTGATTCTCTCTTTTTATTCTCAGCTCCAATTTCAACCTGCATTTCTCT

CTTCTCAATTTTGGGTTTCTTTTTTTGATATCTCATTTGATCAGATCGACGAAGAGGT

AACCATTTTGCCGTTTGTTAGTTCTTAACGATTTTATTCTGTTAAAAATCAGATTTTTT

TTTGCAATGCTTGTAGAAAAAGATTCGATTTTTGTGTACTGATTGAATCAAAATATG

TGGTTATGGATCCATGGATTGATTTGTTTGAACTGATGCAAATGGCTAATGTGAAAA

GTCAAGAATGTGTTAATGTAGTTTTTTTTGTTAAACCTCTTTGTGTTGTTGGGTTTTG

AAATGTTGTTGTTACAGAGGAAGAAGGTTTGAGCTGAGAGATCTTTGGAGGTAGAA

AGAAGATCTCAAAAACGATGTCGTTTACAGGAACTCAGCAGAAATGCAGGGCGTGC

GAGAAGACAGTGTACCCTGTGGAGCTTCTCTCAGCTGATGGAATCTCTTATCACAAG

GCTTGCTTCAAATGCTCTCACTGCAAAAGCAGACTTCAAGTCAGTTTTCCTCTTTTGC

CTTTCCTAAATGTAGTTATTAGTGTTTTGAGAGTTTCATTTAGATATTTAGCTAGAGA

GATACTTGATCTGAATTGGTCTTCGTTAAAGTGATAAAGTTAAAACGTGTTGATGGT

TTCTGAACAGCTGAGCAATTATTCATCAATGGAAGGAGTGGTGTATTGTAGGCCTCA

TTTTGAGCAGCTTTTCAAGGAGTCTGGTAGTTTCAGCAAGAACTTTCAGTCACGTAT

GTAAAACTCTTTTCTTCACCTTCTGGTCCAGCTTTTACATGTGGTCACTTTAATTTTAA

TCTTCAAACTTTTTTTTTCCTTTCACAGCTGCAAAGCCATTGACTGATAAGCCAACCC

CGGAGCTGGTAAACGCTTCTTAACAACGATGTTTTTATTTAGTTACACGACTTGAGA

TACATGACTCTATTTCTCTTCTTGGTTGTTCTCAGAATAGGACACCTAGTCGACTTGC

TGGTATGTTCTCTGGAACGCAAGACAAATGCGCTACTTGCACTAAAACCGTGTATCC

TATCGAAAAGGTTAGTATACATTTAAGCTTGTATATGTCATTACCATTGCTTTCCAAT

ACACTTTTGTGTTCTCTCTGTGATTGTTGTCAAGAAAATCACAGTTAAACTTATTTGG

ATCATTTGCAATATGAGTTACATGGATCCGAATCACATAAAGTTGAGATCTCTTTAA

TACAGTCAATTCTTATATCTCATTGTTCTGTTTTCTAAAAATTGTAGGTAACTGTGGA

AAGCCAGTGTTACCATAAATCTTGCTTCAAGTGTTCTCATGGAGGCTGTCCGATATC

TCCATCGAACTATGCAGCTCTAGAGGGAATATTGTACTGCAAGCACCATTTCGCTCA

GCTTTTCAAGGAGAAAGGAAGTTACAATCATCTCATCAAATCTGCTTCCATCAAACG

CGCTACTGCTGCTGCTACCGCAGCTGCTGCAGCTGTAGCAGCCGTTCCCGAATCTTG

AATTCAAATTCTCTTTATTTTTGTGTATGTGCTGTGTTGTTTACATGTATTCGATATGA

TTCATGTTCTTTGTCTTCCATAATTGAACCAGGCATAAAATTCTCCCAGTTTTCTCTG

AGTTTCTTGTTTGTGATACTACTACATCTATAATAACCGAGGACTTTTGGCTTT

>AtWLIM2b

AATAAAAAAAATATACAGAGGACAAGCCAAAGTGGAACCCCACCCACTGTCTTCTC

AAAGATTAAAAAAAAGAAAAGGATTTTACCCAAAATCTCCGATATTCTTATTCCTTT

GCTGTATCATCTTCTTCTTCTACTTCGTCCTCATCATCGTCACATAGATCCTCCTTTTT

CGTTAATTGGGGAGAGATATACGTAACAGCTCTTCTCTCTACAAAGAAGTTGACTTT

TGCATCCTTACATCTCTCAAATCTTTCTCTGGTTAATCTGTATTCGTGTATTTTTGCAG

TTTCTTTTGAGGATCAGATCGTGACAGGGTTTAGCTCAAGGAGAGGTATTAGATCCG

TACTTTGTGATAAACTTTCTCAAGGTTTACGATCTCATTCTATCTAGATCGGCTTCGT

ATAGATACAATCTTGTTTCAATTAGCAATGGATCGATTTTGTTATTCGAATATTTAGA

AAAAGGAAAGATTTTTCCAAAGGGGTTTCCTCTTATACCATTTTTTCTGGGTTTTTTG

AAATTTAGGGTGAGAAAAAGAAGAAGAAGGTGAGAAAAGTGAAGGCAAGGCTTTG

AGGATGTCTTTTACAGGAACTCAACAGAAATGCAAGGCTTGTGAGAAGACTGTTTAT

GCTGTTGAGCTTCTCTCTGCTGATGGAGTTGGATATCACAAGTCTTGCTTCAAATGCA

CTCACTGCAAAAGCAGGCTTCAGGTCTGATTCTCTCATCTTTGTATTCATGTTAACGA

TGCTGCTTTAATTAGAGGAATGAGTTTACGAATTACCCATCTTCTTGCTTAAATGATG

AATTGAATGTCTATTGTAACCATAAGCAGCTCTTGATAAAGTAAATGTCTTTTTACA

GCTGAGTAGTTACTCATCAATGGAAGGTGTTTTGTACTGTAAGCCTCATTTTGAGCA

GCTCTTTAAGGAGAGTGGTAGTTTCAACAAGAACTTTCAGTCACGTATGTAGTTTTG

TTTCTGCTTCGATTCATGTTTTTTTTCCGCTTGCCCTTCTTTTGGTTATTGATTTAATCA

GTCTTGCAACTTTGTTTCCTCACAGCTGCAAAATCGGCTGACAAATCAACTCCTGAG

CTGGTAAATGTTTCTTTTCTGGTTTGGTCAGATGCTCTTTGGATACATTTTCTGATTG

AACTTTTCTCTTATATTTCCTTCTTGGTTTGTTTTCAGACAAGGACGCCTAGCCGAGT

TGCTGGCAGGTTCTCTGGTACACAAGAGAAATGCGCCACTTGTAGTAAAACTGTGTA

TCCTATTGAAAAGGTTATAACTCGTTTAATCTTTCATTGCTCTTCCTTCACTCATGCA

TTTTCCATTAACTGAAGCTTTGGAATCACCTTTCTGCACTCTAAGGTCTATGTTGGAG

TATTACTCTAAAACACAGTTCCATCATTTGCAAGAATTCTCCTGTGTTTTCTATTTCA

TGTTGAGGATAGATACATAATCCCCTCAGTTACCGAGAACTTGCAAGGAAACCAAA

TGTTTTGCACAGATGCATAGACCCTGGAGATATCGGTTCTTGTTACTTTAATTTGCAT

GTTAAACCTATAAAGGAGACTTAATATGAAAACTCTTTATGGGATTTTTAAAAGCTA

TTTGGTCACAAATATCCGTCTTTTTCGATTCTGAAATATGCAGGTAACAGTCGAGAG

CCAGACATATCACAAGTCCTGCTTCAAGTGCTCACATGGAGGTTGCCCAATTTCACC

TTCCAACTACGCAGCTCTTGAAGGAATCCTGTACTGCAAGCACCATTTCGCTCAGCT

CTTCAAGGAGAAGGGAAGTTACAACCACTTAATCAAATCCGCTTCCATCAAACGCTC

TGCAGCCGCAGCAGTCGCCGCCGGTGTACCAGCAGCCTCCGTTCCTGAATCTTAAAA

TCCAATAATTTCTCCTCTCTAGTCTAAAATTTGAAGTTAATGCCAAACTGATTCAGTT

TGATTTCTCTTTGTGAGAGCGTGTGTGTAATCTATGCTTTGATGTTCTTATTTCCATTG

ATGTCTCCTAAAATTCTATGTTTGTGTGGTGATTTGGTTATCTATAAAAAGTCTGAAA

CTTTCCTTATAAATCTTCAATTTCGTTT

>AtPLIM2a

ACCACAAATTCTCATTTCTTCAAGAATTTTTGATAAATAAGACAGAGAACTTGAGAG

ATGAAAAGAAAAAAACACAAAACCTGGTGTTTTTCTCTCGTCGTCGTTTGGTCGTG

TCCTAAGACAAGAACCGGCGAACGAAGAACAAACTTTTTGAATCTAACATCCGAAA

GAAAGAGAGAAAATGTCGTTTACAGGAACATTGGATAAATGCAAGGCCTGTGACAA

AACCGTGTACGTGATGGATTTGTTAACATTAGAGGGTAATACTTATCACAAATCATG

TTTCAGATGCACCCATTGCAAAGGCACTCTCGTGGTATTTCTCTTTTCTTCTTCTTCCT

CCATTGTTACTCTGTTTTTCGACAATGCTTCATTGTTTTAAAAGACCACATCTTTTCTT

AACACAGATAAGCAATTACTCATCAATGGATGGAGTTCTTTACTGTAAGCCACACTT

CGAACAGCTTTTCAAAGAATCTGGCAATTACAGCAAGAATTTTCAGGCAGGAAAGA

CCGAGAAGCCCAATGATCATCTGGTAATGTCTTCAAAGATTCATCATCTTGTTTTTAT

TTCCTCTTTTTTTTCATCTGATTTGTCTCAATTGATGGATACTGCAAGACTCGAACTC

CAAGCAAGTTATCATCATTCTTCAGTGGAACACAAGACAAATGTGCGACTTGTAAGA

AGACGGTTTACCCACTTGAGAAAGTAACAATGGAAGGAGAAAGTTACCACAAGACT

TGCTTCAGGTGCACACACAGTGGTTGTCCTTTGACTCACTCTTCTTACGCTTCTCTTA

ATGGCGTCCTCTACTGTAAAGTCCACTTCAATCAGCTCTTTCTCGAGAAAGGCAGTT
ACAATCACGTCCATCAAGCCGCTGCTAACCACCGTCGATCCGCCTCTTCTGGTGGCG
CTTCTCCTCCTTCTGATGATCACAAACCTGACGACACCGCCTCAATTCCCGAAGCAA
AAGAAGATGACGCTGCCCCTGAAGCTGCAGGAGAAGAGGAGCCTGAGCCGGTCGTT
GAGTCTTGAAAATGTCGTGGAGGAATGATCTTATCAGTTCTCAAGTTTGAAGTTTTT
ATATGCGTTTCTGTTTAATGAATATATAAATACAAATTGTTGTTGTTTCTTCTTGTAA
ATTCTTGTAAAGTTTTAAAGCTCTTTTTGGAGAGAATCTGTTGTAAACAAAATCTTTA
ACATCCAATTTTTTTTGAAGAGGATATTTTTAACCTTTAGTTTTAAAA

>AtPLIM2b

TCTGAGGTAAAGGTTTCCTTCTTTAACTAATTCCTCAGATGACCTCTTGATTTGTTGG
AAAAAAAATTAAAAAATCTGTTTCATACAGGATCTTGGAGAAGGAAGGAAACAACA
ACAACCAAAAAAAAACCAAAAAAAAAAAGATAAAACAAAAAAAATGTCTTTCAC
AGGAACTCTCGACAAATGCAATGTTTGTGATAAGACAGTCTATGTGGTGGACATGTT
GTCCATTGAAGGAATGCCTTACCACAAGTCTTGCTTCAGGTGTACCCATTGCAAAGG
AACCCTTCAGGTGCACTCATTACTCATTTTCTTGTTAGATCTTCTATGATCATATTAT
ATCAACAATTTATCTATGTGTTGATGGCAGATGAGCAACTATTCCTCCATGGACGGA
GTTTTGTACTGCAAGACTCATTTTGAGCAACTCTTCAAGGAATCTGGCAATTTCAGC
AAAAACTTTCAACCAGGTTCGTATTATAAAATCTCTGACATCGAATCTCTCTATCTTT
CAAATCATCATGATCATAACAAGTCTCTTTCTTTTCTCAGGAAAAACTGAGAAGCCA
GAGCTGGTAGGTTCATCATTACATGTCCTTGTATATGATTAGCGTGTCAATATTTCAT
TTTCTTTCTTTTATGATCGACTAATAGGATCTGTGTACTGATTTAAACATTACAGACT
AGGACTCCCAGCAAGATATCTTCCATCTTCTGTGGAACACAAGACAAGTGTGCCGCT
TGCGAAAAAACTGTTTACCCTCTTGAAAAGGTTAGTGGTATACGCATCTGCAAATGT

TCTCATGGCCTAAAATACCAACGAACTAGTCTCGTAGAGAAATTTATTTGCTCTAGA
ATTTGTTTTTTGTTTTTTCATTTCCGTAGAATTTTTCTGTTCGGATAAAATCATTACTA
GATCAAATGGAAATAAAATTTTGATTTTGGTTTGGTTTAGTATACTTTTGGTTCGATT
TGAGAACGTGATGTTGGTTGGTCCAAGCAAATGTGGAATCAATTTTGGCTTGGTTCT
AATCGGGTTATTTGGTAACAGATACAAATGGAAGGAGAATGCTTCCACAAGACATG
TTTCCGGTGCGCTCACGGTGGGTGTACGCTGACTCACTCCTCCTACGCCTCCTTAGAT
AGCGTTCTCTATTGCCGACATCACTTTAACCAACTCTTCATGGAGAAAGGAAACTAC
GCTCACGTCCTCCAAGCCGCCAATCACCGTCGCACAGCCTCAGGCAACACTCTTCCA
CCGGAACCTACCGAAGACGTCGCCGTGGAGGCCAAGGAAGAGAATGGCGTTTCAGA
GTCTTGAATATCTCTTACATGAGTGTTTTTGAGTGAATGATTGAATGAATGTTGTTGC
GGGGGAAAGATTTGATTTCATTTGTAATGAGTTGTTTCCATATGAAGACATGATTTTT
TTTGTTATTTTTTTAATTCATTCGTTATACATTTATTATTACACACGAAAAAAA
>AtPLIM2c
TTCGTGTCTAAACGAACAACAAAAAGAAGAAGAAAACTTTTGATCCATCTTCCAACT
CTTTCGTAATTTGAAAGAAGAAGAAGAAGAGATACCAATGGCGGCGTTTACAGGGA
CAACAGACAAATGCAAGGCGTGTGACAAGACGGTATACGTTATGGACTTGATGACT
TTGGAAGGAATGCCTTATCACAAGTCATGCTTCAGGTGCAGCCATTGCAATGGCACT
CTCGTGGTATCTATCTATCTTCCTTTTCCTCCATTGTTCCCTCATGAAAAAAAAAACA
CAAAGAGAACATCTTTCTTCTCCTTTTCAGATATGTAACTACTCATCCATGGATGGA
GTTTTGTATTGCAAGACCCATTTTGAACAGCTCTTCAAAGAATCTGGAAATTTCAGC
AAGAATTTCCAAACAGGTTGCTAACAAATCCTCTGTTTTCTTTGCTCTGTTTTTTATT
AACCTCTCTTGAAAAGCTAACAATCTTTGCTACTTTCTCTTCTTGGTCTTTTACAGCT
GGAAAGACCGAGAAATCGAATGATGCGGTAAGCACATAATTCATCAACATTCCTCT

GTTTTCTCTTTTTGTTTTCTAATCTGATTTCTTGGTGTGATCATTTGCATAGACGAAGG

CTCCAAACAGGTTATCATCCTTCTTTAGCGGCACACAAGACAAATGTGCAGCTTGTA

AGAAAACAGTTTATCCTCTAGAGAAGATGACAATGGAAGGAGAATCTTACCACAAG

ACTTGCTTCAGATGTGCACATAGTGGTTGTCCATTAACACATTCTTCATATGCTGCTC

TCGATGGCGTCCTTTACTGTAAGGTCCACTTCAGCCAGCTTTTCCTTGAGAAAGGTA

ATTACAATCATGTCCTCCAAGCCGCGGCTAACCACCGTCGCTCGACGGCTGAGGAA

GACAAAACCGAACCCAAAGAAGATGAAGCAAACCCTACAGAAGAAGAAACTTCTG

ATGCAGCAGCTGAAGAACATGAATCCTAAGAAATATCCGGGAGTTCCTTTTGTGAA

ATATATATAATCATAAATTGTTAAAGAGATAATTTCTTAACTAATGCAATGAAGTAG

ACTCAAATTTTGTTTAGTTTCTTCTTATGGAACTATATATTATTCTGTTTGTAAAGTA

ATTATATTCAAGTTTATGAGTAATTACCAAAACA
