# Supplementary material for: A genome‑wide approach to the systematic and comprehensive analysis of LIM gene family in sorghum (Sorghum bicolor L.)
Source: Genomics Inform. 2023 Sep 27;21(3):e36. doi: 10.5808/gi.23007 (PMC10584642; doi:10.5808/gi.23007)
Supplement: Supplementary Fig. 2. — Full-length coding sequences of LIM gene families of Arabidopsis thaliana (Doc). [file gi-23007-Supplementary-Fig-2.pdf]

Supplementary Fig. 2. Full-length coding sequences of LIM gene families of *Arabidopsis thaliana* (Doc).

>AtWLIM1

ATGGCGTTCGCAGGAACAACCCAGAAATGCATGGCATGTGACAAAACAGTTTATCT
TGTCGACAAGTTAACCGCCGATAACCGGGTCTACCACAAAGCTTGTTTCCGATGTCA
CCATTGCAAAGGAACTCTCAAGCTTAGCAATTACAACTCCTTTGAAGGAGTTCTCTA
CTGCAGACCACATTTCGATCAAAACTTCAAGAGAACTGGAAGTCTTGAGAAAAGCT
TCGAAGGGACACCAAAGATTGGGAAACCTGATAGGCCTTTGGAGGGAGAGAGACCT
GCTGGAACCAAAGTTTCGAATATGTTTGGTGGAACACGAGAGAAATGCGTTGGTTG
CGACAAAACCGTGTATCCAATTGAGAAGGTATCGGTGAATGGAACATTGTACCACA
AGAGCTGCTTCAAGTGTACACATGGAGGCTGCACGATAAGCCCTTCGAATTACATAG
CTCACGAGGGTAAGCTATATTGCAAGCATCATCATATTCAGCTGATCAAGGAGAAA
GGAAACTTGAGCCAGCTCGAAGGAGGAGGAGAGAATGCCGCCAAGGACAAAGTCG
TCGCTGCTTAA

>AtWLIM2a

ATGTCGTTTACAGGAACTCAGCAGAAATGCAGGGCGTGCGAGAAGACAGTGTACCC
TGTGGAGCTTCTCTCAGCTGATGGAATCTCTTATCACAAGGCTTGCTTCAAATGCTCT
CACTGCAAAAGCAGACTTCAACTGAGCAATTATTCATCAATGGAAGGAGTGGTGTA
TTGTAGGCCTCATTTTGAGCAGCTTTTCAAGGAGTCTGGTAGTTTCAGCAAGAACTTT
CAGTCACCTGCAAAGCCATTGACTGATAAGCCAACCCCGGAGCTGAATAGGACACC
TAGTCGACTTGCTGGTATGTTCTCTGGAACGCAAGACAAATGCGCTACTTGCACTAA
AACCGTGTATCCTATCGAAAAGGTAACTGTGGAAAGCCAGTGTTACCATAAATCTTG
CTTCAAGTGTTCTCATGGAGGCTGTCCGATATCTCCATCGAACTATGCAGCTCTAGA
GGGAATATTGTACTGCAAGCACCATTTCGCTCAGCTTTTCAAGGAGAAAGGAAGTTA
CAATCATCTCATCAAATCTGCTTCCATCAAACGCGCTACTGCTGCTGCTACCGCAGC
TGCTGCAGCTGTAGCAGCCGTTCCCGAATCTTGA

>AtWLIM2b

ATGTCTTTTACAGGAACTCAACAGAAATGCAAGGCTTGTGAGAAGACTGTTTATGCT
GTTGAGCTTCTCTCTGCTGATGGAGTTGGATATCACAAGTCTTGCTTCAAATGCACTC
ACTGCAAAAGCAGGCTTCAGCTGAGTAGTTACTCATCAATGGAAGGTGTTTTGTACT
GTAAGCCTCATTTTGAGCAGCTCTTTAAGGAGAGTGGTAGTTTCAACAAGAACTTTC
AGTCACCTGCAAAATCGGCTGACAAATCAACTCCTGAGCTGACAAGGACGCCTAGC
CGAGTTGCTGGCAGGTTCTCTGGTACACAAGAGAAATGCGCCACTTGTAGTAAAACT
GTGTATCCTATTGAAAAGATACATAATCCCCTCAGTTACCGAGAACTTGCAAGGAAA
CCAAATGTTTTGCACAGATGCATAGACCCTGGAGATATCGGTTCTTGTTACTTTAATT
TGCATGTAACAGTCGAGAGCCAGACATATCACAAGTCCTGCTTCAAGTGCTCACATG
GAGGTTGCCCAATTTCACCTTCCAACTACGCAGCTCTTGAAGGAATCCTGTACTGCA
AGCACCATTTCGCTCAGCTCTTCAAGGAGAAGGGAAGTTACAACCACTTAATCAAAT

CCGCTTCCATCAAACGCTCTGCAGCCGCAGCAGTCGCCGCCGGTGTACCAGCAGCCT
CCGTTCCTGAATCTTAA

>AtPLIM2a

ATGTCGTTTACAGGAACATTGGATAAATGCAAGGCCTGTGACAAAACCGTGTACGT
GATGGATTTGTTAACATTAGAGGGTAATACTTATCACAAATCATGTTTCAGATGCAC
CCATTGCAAAGGCACTCTCGTGATAAGCAATTACTCATCAATGGATGGAGTTCTTTA
CTGTAAGCCACACTTCGAACAGCTTTTCAAAGAATCTGGCAATTACAGCAAGAATTT
TCAGGCAGGAAAGACCGAGAAGCCCAATGATCATCTGACTCGAACTCCAAGCAAGT
TATCATCATTCTTCAGTGGAACACAAGACAAATGTGCGACTTGTAAGAAGACGGTTT
ACCCACTTGAGAAAGTAACAATGGAAGGAGAAAGTTACCACAAGACTTGCTTCAGG
TGCACACACAGTGGTTGTCCTTTGACTCACTCTTCTTACGCTTCTCTTAATGGCGTCC
TCTACTGTAAAGTCCACTTCAATCAGCTCTTTCTCGAGAAAGGCAGTTACAATCACG
TCCATCAAGCCGCTGCTAACCACCGTCGATCCGCCTCTTCTGGTGGCGCTTCTCCTCC
TTCTGATGATCACAAACCTGACGACACCGCCTCAATTCCCGAAGCAAAAGAAGATG
ACGCTGCCCCTGAAGCTGCAGGAGAAGAGGAGCCTGAGCCGGTCGTTGAGTCTTGA

>AtPLIM2b

ATGTCTTTCACAGGAACTCTCGACAAATGCAATGTTTGTGATAAGACAGTCTATGTG
GTGGACATGTTGTCCATTGAAGGAATGCCTTACCACAAGTCTTGCTTCAGGTGTACC
CATTGCAAAGGAACCCTTCAGATGAGCAACTATTCCTCCATGGACGGAGTTTTGTAC
TGCAAGACTCATTTTGAGCAACTCTTCAAGGAATCTGGCAATTTCAGCAAAAACTTT
CAACCAGGAAAAACTGAGAAGCCAGAGCTGACTAGGACTCCCAGCAAGATATCTTC
CATCTTCTGTGGAACACAAGACAAGTGTGCCGCTTGCGAAAAAACTGTTTACCCTCT
TGAAAAGATACAAATGGAAGGAGAATGCTTCCACAAGACATGTTTCCGGTGCGCTC
ACGGTGGGTGTACGCTGACTCACTCCTCCTACGCCTCCTTAGATAGCGTTCTCTATTG
CCGACATCACTTTAACCAACTCTTCATGGAGAAAGGAAACTACGCTCACGTCCTCCA
AGCCGCCAATCACCGTCGCACAGCCTCAGGCAACACTCTTCCACCGGAACCTACCG
AAGACGTCGCCGTGGAGGCCAAGGAAGAGAATGGCGTTTCAGAGTCTTGA

>AtPLIM2c

ATGGCGGCGTTTACAGGGACAACAGACAAATGCAAGGCGTGTGACAAGACGGTATA
CGTTATGGACTTGATGACTTTGGAAGGAATGCCTTATCACAAGTCATGCTTCAGGTG
CAGCCATTGCAATGGCACTCTCGTGATATGTAACTACTCATCCATGGATGGAGTTTT
GTATTGCAAGACCCATTTTGAACAGCTCTTCAAAGAATCTGGAAATTTCAGCAAGAA
TTTCCAAACAGCTGGAAAGACCGAGAAATCGAATGATGCGACGAAGGCTCCAAACA
GGTTATCATCCTTCTTTAGCGGCACACAAGACAAATGTGCAGCTTGTAAGAAAACAG
TTTATCCTCTAGAGAAGATGACAATGGAAGGAGAATCTTACCACAAGACTTGCTTCA
GATGTGCACATAGTGGTTGTCCATTAACACATTCTTCATATGCTGCTCTCGATGGCGT
CCTTTACTGTAAGGTCCACTTCAGCCAGCTTTTCCTTGAGAAAGGTAATTACAATCAT
GTCCTCCAAGCCGCGGCTAACCACCGTCGCTCGACGGCTGAGGAAGACAAAACCGA
ACCCAAAGAAGATGAAGCAAACCCTACAGAAGAAGAAACTTCTGATGCAGCAGCTG
AAGAACATGAATCCTAA
