# Supplementary material for: A genome‑wide approach to the systematic and comprehensive analysis of LIM gene family in sorghum (Sorghum bicolor L.)
Source: Genomics Inform. 2023 Sep 27;21(3):e36. doi: 10.5808/gi.23007 (PMC10584642; doi:10.5808/gi.23007)
Supplement: Supplementary Fig. 3. — Full-length peptide sequences of LIM gene families of Arabidopsis thaliana (Doc). [file gi-23007-Supplementary-Fig-3.pdf]

Supplementary Fig. 3. Full-length peptide sequences of LIM gene families of *Arabidopsis thaliana* (Doc).

>AtWLIM1

MAFAGTTQKCMACDKTVYLVDKLTADNRVYHKACFRCHHCKGTLKLSNYNSFEGVL
YCRPHFDQNFKRTGSLEKSFEGTPKIGKPDRPLEGERPAGTKVSNMFGGTREKCVGCDK
TVYPIEKVSVNGTLYHKSCFKCTHGGCTISPSNYIAHEGKLYCKHHHIQLIKEKGNLSQL
EGGGENAAKDKVVAA

>AtWLIM2a

MSFTGTQQKCRACEKTVYPVELLSADGISYHKACFKCSHCKSRLQLSNYSSMEGVVYC
RPHFEQLFKESGSFSKNFQSPAKPLTDKPTPELNRTPSRLAGMFSGTQDKCATCTKTVYP
IEKVTVESQCYHKSCFKCSHGGCPISPSNYAALEGILYCKHHFAQLFKEKGSYNHLIKSA
SIKRATAAATAAAAAVAAVPES

>AtWLIM2b

MSFTGTQQKCKACEKTVYAVELLSADGVGYHKSCFKCTHCKSRLQLSSYSSMEGVLYC
KPHFEQLFKESGSFNKNFQSPAKSADKSTPELTRTPSRVAGRFSGTQEKCATCSKTVYPIE
KIHNPLSYRELARKPNVLHRCIDPGDIGSCYFNLHVTVESQTYHKSCFKCSHGGCPISPSN
YAALEGILYCKHHFAQLFKEKGSYNHLIKSASIKRSAAAAVAAGVPAASVPES

>AtPLIM2a

MSFTGTLDKCKACDKTVYVMDLLTLEGNTYHKSCFRCTHCKGTLVISNYSSMDGVLYC
KPHFEQLFKESGNYSKNFQAGKTEKPNDHLTRTPSKLSSFFSGTQDKCATCKKTVYPLE
KVTMEGESYHKTCFRCTHSGCPLTHSSYASLNGVLYCKVHFNQLFLEKGSYNHVHQAA
ANHRRSASSGGASPPSDDHKPDDTASIPEAKEDDAAPEAAGEEEPEPVVES

>AtPLIM2b

MSFTGTLDKCNVCDKTVYVVDMLSIEGMPYHKSCFRCTHCKGTLQMSNYSSMDGVLY
CKTHFEQLFKESGNFSKNFQPGKTEKPELTRTPSKISSIFCGTQDKCAACEKTVYPLEKIQ
MEGECFHKTCFRCAHGGCTLTHSSYASLDSVLYCRHHFNQLFMEKGNYAHVLQAANH
RRTASGNTLPPEPTEDVAVEAKEENGVSES

>AtPLIM2c

MAAFTGTTDKCKACDKTVYVMDLMTLEGMPYHKSCFRCSHCNGTLVICNYSSMDGVL
YCKTHFEQLFKESGNFSKNFQTAGKTEKSNDATKAPNRLSSFFSGTQDKCAACKKTVYP
LEKMTMEGESYHKTCFRCAHSGCPLTHSSYAALDGVLYCKVHFSQLFLEKGNYNHVLQ
AAANHRRSTAEEDKTEPKEDEANPTEEETSDAAAEEHES
