# Supplementary material for: A genome‑wide approach to the systematic and comprehensive analysis of LIM gene family in sorghum (Sorghum bicolor L.)
Source: Genomics Inform. 2023 Sep 27;21(3):e36. doi: 10.5808/gi.23007 (PMC10584642; doi:10.5808/gi.23007)
Supplement: Supplementary Fig. 4. — Full-length genomic sequences of LIM gene families of Arabidopsis thaliana with 2,000 bp upstream region (Doc). [file gi-23007-Supplementary-Fig-4.pdf]

Supplementary Fig. 4. Full-length genomic sequences of LIM gene families of *Arabidopsis thaliana* with 2,000 bp upstream region (Doc).

>AtWLIM1_2000bp

TATGAATTGGCATCATGACATCTTTCAAAGCAATGACATTCTCATGTCGAAGATGGC
GTAGAAGCTTGAGCTCCCGAAGAGTCCTCAACGCATCGATCCTATTCTCATAAACAT
TGTGAATCTTCTTGATAGCAACTTTCTCGTTGGTGTCACTGTTAACAGAGGAGCAGA
CAACACCGTAAGCTCCACGACCAATAGGCTTGATAGGCATGTACTTAGTGTCGATCT
CGAACAGAGTTTGCCACATTGAGAAGTAATGCTTCCCTTCATTCCTTATCCCATTAG
GAGGATCAACCAAAGTCGCCATTTTTTGTTTTCCCTCTTCATCACAATTCACACACAA
TCAAGAAATCAAATTTACACACACAATTAACAAATTATTATACTAACTATTTATCAA
AACTTTTCAAGTGATAAGTTACTGTAGATTTCTTGTGTAAACAGATTGGTCTGTTAAT
AATAGTGAAAAGGAAAAAGATCAAACTTTATCGATTTGGTGAAGGAACATAAACGA
TCGGAATCAATGGGAAAAAAGTTTACGGGATAAAGCGGGAAGGAAATTCTAACCGG
TGATAAGTGACCAACGACGTTCCCCGGAGGTTAGATTTGGCCGGAGAGAGAAACCT
GTGGAAGTGAGAGAGATTTGGCCAACGCTCGGACAGTTGACCTGCTACTTGTTCGTT
ACTAGGTAGTCTTTCCTTATATAATTTGGGCTTAAGTTGAGCCCAATTAATATTGGCA
AACAGATTCACGTCTCTATCTTCTAAAATCAATTTCGGTATTTTTTAGTAACTGATCA
CCAAAGTAGCATCATTTGTGAATATGTGTTCTACGAAGATTTATAAAAATTGAAAAT
GAAGAAAAGTATTTTAAAGTAAAATATATCATAAATGTAAACAATGACACATGACT
AAAATATTGCATCACCAATTCACCATCTTCTTTTGCTTTAACTTTACGTTTCCATCAC
ACATCTTTTCATTTTGCTAAAATTAGCATCTAGAGGAAAATAAACTACAAAAGATTT
AAAAGTTTTCATTAAGTCAGATTATTAATATAAGAGAGGAGGCCCATGCAGACAGA
AAGGGAAAGCCCATAAATCTAAGCAGTGGCAAAAATGTTAGAAACTGTATAAAATG
ATACTAATAAATTGGTTGGAGTCTATTATAGTAGCAAATAAAAATAACGTTAGGTAT
TTTTGATTAAATAAAAGATGTTAGGTATCATTATGATCACTCATTCTCTTCAACCAAT
TATGTCAATACTACAGCTCATCAAAAGCCTTTATGGTAAAGCATGTTTCAGATATTTT
CATTATTCATATACACACTACACAACATATAGTATTAAAGAAAATAAATAGTGTATA
GATATTTAGAGATTTCCAACTAATTCAACATAACCAATACAGCTTTTAATCATTATCT
AAAACCAGACAAAGATTCTTTCCAGTTGGTTTTCCCTGTTCTATTTCCTTACAAACAG
TTATTGACTATTGAGCATTGCCTTCACTGTTGAGAAAAATTTGTGTAAATCTTACGCA
CTGTCAAGCAACTGCATGCATTCTCTAAATAATTACCTGACTCTAGATATAAAAGAA
CTGAATTAATGTAAAAAAAATATAAGCAAAATATCTAGGAGACAGATATTGACGGG
TTACTTCAAGTTTGGACAAAAATAATTAAGAACACAAAAAATGCCGTTGAAACCTTT
TCTTTTTGTAGTACAGTTTTTAGTTTTGTTGCTAACGTTCAAATTATTTATTTATTCAT
CGCTTATCTTGTTTTGTGGATTAGACAACCACGAAAAACAATGCTAGGGTTGTCAGA
AACACCAAATATTCGAATTTGCATCCAAAATTCGATACTCATCTTGAACTTTTTTGTT
TGTGACATTTTATTAAAAATTTCAATTTTTCCTAAAAGAATTTTACTTTAAATATTTA
AAAAGAAGAAGCATGTTCATTTTTCAATATATCTGGTCCCCATATTAAAACAAGCAA
CAATAATTGACAATCTCTTTCTTTCATCTTCATCTTATTCTTCTTTATCTCTCTCTCTCT
CTCAAGACTATTGAGCTTCTTTCTTTTTTCCTGAGTCTTTTAGTCTGATCTCTTACAAG
AATGGCGTTCGCAGGAACAACCCAGAAATGCATGGCATGTGACAAAACAGTTTATC

TTGTCGACAAGTTAACCGCCGATAACCGGGTCTACCACAAAGCTTGTTTCCGATGTC
ACCATTGCAAAGGAACTCTCAAGGTTTGATCTTTGATTCTAGTTTGTGATGATTTCTC
ATTAATCCAGAAGTAGATCTAGTAGATATATATGTAGATGAAGAAGTCCAATCAATT
AAGTAGAAATCTTTGAATTTTTGGCTATGGAAACTGATTGGTCTAAAGATTTGACTG
TTCTGTTTCTTCTTCTTCTTCTTCTTCTTCTTGATGAGTCAGCTTAGCAATTACAACTC
CTTTGAAGGAGTTCTCTACTGCAGACCACATTTCGATCAAAACTTCAAGAGAACTGG
AAGTCTTGAGAAAAGCTTCGAAGGTAAATTACCAAAAACAGATCAAAATGTGAAAA
GCTTTGTGAGTGGGGATTTCAAATTTGGAATTATCTATCTAAAGTTTGATTCATATAT
ATGTACAGGGACACCAAAGATTGGGAAACCTGATAGGCCTTTGGAGGGAGAGGTAC
TTTCGGACAGCAGAACAAAACCCTTTTTCTGTTTTTGAGGTTCTTGAATTTTTTTAAC
ATTTAACATGAGAGAAGTTTAACAGAGACCTGCTGGAACCAAAGTTTCGAATATGTT
TGGTGGAACACGAGAGAAATGCGTTGGTTGCGACAAAACCGTGTATCCAATTGAGA
AGGTATCCAATCTCAAATCTTTCCTGATCAGAATCAAGTTGTGTCAAAGAAGATTGT
TAGATTGTTTCCATAACGAATCTGATCTGTTCTTGGCTTAAACCAGAGCTGAAACAT
TGCTTATAATGTGGACTTTGTGTAGGTATCGGTGAATGGAACATTGTACCACAAGAG
CTGCTTCAAGTGTACACATGGAGGCTGCACGATAAGCCCTTCGAATTACATAGCTCA
CGAGGGTAAGCTATATTGCAAGCATCATCATATTCAGCTGATCAAGGAGAAAGGAA
ACTTGAGCCAGCTCGAAGGAGGAGGAGAGAATGCCGCCAAGGACAAAGTCGTCGCT
GCTTAAAGAAACAATTCAATCTCTCTGGGATGATTTTGACTATCGAAGAATCAAAAG
AGTCACTACTTCTCCATCGATCGATAACATATATTGGTTTTGTTTCTTCCTACGTTTGT
GTTTTTCTTTTTGGTCTTTAATATGTTTTTTTTTTTTTGGGACCTTTGTTCACTCTTTTA
TTGGGTTTCTTGAAATGAAACTAGAGAAACAAAGAAATAATGGATGTTTGCTTATGA
GATATATATGTATTACGTTCATATGATATAAATTAATTTTCGTGAAGTTTAAGCTTCA
GC

>AtWLIM2a_2000bp

GCTTCATGAGTTTGGTGGCAGAAGACACATTCGCTATAGAGACCTTGCTGGATTCAT
TTACGGTAAATAAAAGACCAAACCAGCTCTCTTAATCTCTTCTCTGTTGTTACCTTCA
TTGGAATGATCTCTCTTTTTATCAGGTAGGAAGGCTTATCATCTTACATGGGGATTGC
AATATGTTAATCTTTTCATGATTAATTGTGGATTCATTATCCTAGCTGGTTCCGCCTT
AAAGGTAGTAAAACATTTTCTTCTCTTGATTCTTGATTTCGTTAGACGAGTTAGTTAA
TTTTGGTTGTGTGTTCTGTTTCTGCAGGCTGTTTATGTGCTTTTCAGGGATGATCATA
CCATGAAACTACCTCATTTTATAGCCATTGCTGGTTTGATTTGTGCGATTTTCGCTAT
TGGGATTCCTCATTTATCGGCTCTTGGGGTCTGGCTTGGAGTTTCGACCTTCCTCAGT
CTCATCTATATTGTTGTGGCAATCGTGCTATCGGTTAGAGATGGTATAACTAATCAA
ATGATCATCAAGATTCAACATTACTAATGAGTTTAACTACTGCAAAAATCTAATTCT
TGCTTTGTTTTGTTTGTTTCTAGGAGTAAAAACACCTTCAAGAGATTACGAGATACA
AGGATCATCATTGAGCAAACTTTTTACCATCACAGGAGCAGCCGCAAATCTGGTTTT
CGCATTCAACACCGGGATGCTTCCAGAAATTCAGGTGACTTTTCCCTAGACTTCAAA
GTTCAATTGTTTGTTTGTGTTTGCTTTTATGTATTAGTGTACCTTTTGTTCAAGTAAAA
CCTGCATCTTAAACAAAGTTTGTTTGTTAATCATATTGACACTATAATTTCCACAATG
TTAGGCAACAGTGAGGCAACCAGTTGTTAAAAACATGATGAAGGCTCTATACTTTCA
ATTCACAGCGGGTGTTTTACCGATGTACGCAGTTACATTCATCGGATATTGGGCTTA

TGGATCCTCAACCTCGACCTATCTACTAAACAGTGTTAATGGCCCACTCTGGGTCAA
AGCCCTTGCTAACGTCTCTGCTATCCTTCAATCTGTTATCTCTTTGCACGTAAGTTTCT
TAGCGCCAAGTCTACGCTTTTTTAACAAAGACTGTCGTCTCTAACTATATGTCTGACT
CAATTCTTTGTTGTAGATATTTGCGAGTCCAACATATGAGTACATGGACACAAAGTA
TGGGATCAAAGGAAACCCATTTGCGATAAAGAACCTGCTGTTTAGGATCATGGCGA
GAGGCGGGTACATAGCGGTAAGCACGCTTATCTCAGCGCTGTTGCCGTTCCTCGGTG
ACTTCATGAGCCTAACTGGTGCAGTGAGCACATTCCCTCTCACATTCATTCTAGCCA
ACCACATGTACTATAAGGCAAAGAACAATAAGCTGAATGCTATGCAAAAGCTATGG
CATTGGCTTAACGTTGTCTTCTTTAGTTTGATGTCTGTTGCTGCAGCCATTGCAGCTG
TCAGACTCATCGCCGTTGATTCCAAAAATTTCCATGTTTTTGCAGATTTGTAATTCAT
TATATTATATAGTCAATTGTGTATCCTTTATTCTTAGGCAGGTTTATTTCTCCTGTAA
GCTTGTATTCTCTCTCTATTTGTCTTACCATTAGAGCCTAAATAAATAAATCAAGTTC
TAAAATCTAAAAAGCATATAAAAGACCACTTTCATCATAATCATTCTGAAAATTTAC
CAACAAAGAATACTAAGTTGCATGATTAAAGTCTCTATTAGAAATGTAAATCATAGT
ATTAGAGAAGAACATAAAAACACCTAACTTAAAAAAACCTTAATTTAGTGAGATAG
AGAAGAGAAGAAACAGAATTGTTGTTGGTTTAACAAAAACCTAATCTTTCAATGATT
AGCTGTTTAATTTGAAATTATAACAATAATTTAGACAGGCCAAAGTGGAACCCCACT
GTCTCCACCAAAGCTCTAGATAAAAAAAAAAAAAACGAAAGCGTTAGATATTTTTTAT
CAATCGTGCTCCGTAATCATTACCTTTCTTCTTCATCTTCATCTTCACGCAGCGACTT
CTTTGATTCTCTCTTTTTATTCTCAGCTCCAATTTCAACCTGCATTTCTCTCTTCTCAA
TTTTGGGTTTCTTTTTTTGATATCTCATTTGATCAGATCGACGAAGAGGTAACCATTT
TGCCGTTTGTTAGTTCTTAACGATTTTATTCTGTTAAAAATCAGATTTTTTTTGCAAT
GCTTGTAGAAAAAGATTCGATTTTTGTGTACTGATTGAATCAAAATATGTGGTTATG
GATCCATGGATTGATTTGTTTGAACTGATGCAAATGGCTAATGTGAAAAGTCAAGAA
TGTGTTAATGTAGTTTTTTTTGTTAAACCTCTTTGTGTTGTTGGGTTTTGAAATGTTGT
TGTTACAGAGGAAGAAGGTTTGAGCTGAGAGATCTTTGGAGGTAGAAAGAAGATCT
CAAAAACGATGTCGTTTACAGGAACTCAGCAGAAATGCAGGGCGTGCGAGAAGACA
GTGTACCCTGTGGAGCTTCTCTCAGCTGATGGAATCTCTTATCACAAGGCTTGCTTCA
AATGCTCTCACTGCAAAAGCAGACTTCAAGTCAGTTTTCCTCTTTTGCCTTTCCTAAA
TGTAGTTATTAGTGTTTTGAGAGTTTCATTTAGATATTTAGCTAGAGAGATACTTGAT
CTGAATTGGTCTTCGTTAAAGTGATAAAGTTAAAACGTGTTGATGGTTTCTGAACAG
CTGAGCAATTATTCATCAATGGAAGGAGTGGTGTATTGTAGGCCTCATTTTGAGCAG
CTTTTCAAGGAGTCTGGTAGTTTCAGCAAGAACTTTCAGTCACGTATGTAAAACTCT
TTTCTTCACCTTCTGGTCCAGCTTTTACATGTGGTCACTTTAATTTTAATCTTCAAACT
TTTTTTTTCCTTTCACAGCTGCAAAGCCATTGACTGATAAGCCAACCCCGGAGCTGGT
AAACGCTTCTTAACAACGATGTTTTTATTTAGTTACACGACTTGAGATACATGACTCT
ATTTCTCTTCTTGGTTGTTCTCAGAATAGGACACCTAGTCGACTTGCTGGTATGTTCT
CTGGAACGCAAGACAAATGCGCTACTTGCACTAAAACCGTGTATCCTATCGAAAAG
GTTAGTATACATTTAAGCTTGTATATGTCATTACCATTGCTTTCCAATACACTTTTGT
GTTCTCTCTGTGATTGTTGTCAAGAAAATCACAGTTAAACTTATTTGGATCATTTGCA
ATATGAGTTACATGGATCCGAATCACATAAAGTTGAGATCTCTTTAATACAGTCAAT
TCTTATATCTCATTGTTCTGTTTTCTAAAAATTGTAGGTAACTGTGGAAAGCCAGTGT
TACCATAAATCTTGCTTCAAGTGTTCTCATGGAGGCTGTCCGATATCTCCATCGAACT

ATGCAGCTCTAGAGGGAATATTGTACTGCAAGCACCATTTCGCTCAGCTTTTCAAGG
AGAAAGGAAGTTACAATCATCTCATCAAATCTGCTTCCATCAAACGCGCTACTGCTG
CTGCTACCGCAGCTGCTGCAGCTGTAGCAGCCGTTCCCGAATCTTGAATTCAAATTC
TCTTTATTTTTGTGTATGTGCTGTGTTGTTTACATGTATTCGATATGATTCATGTTCTT
TGTCTTCCATAATTGAACCAGGCATAAAATTCTCCCAGTTTTCTCTGAGTTTCTTGTT
TGTGATACTACTACATCTATAATAACCGAGGACTTTTGGCTTT

>AtWLIM2b_2000bp

AGTCACCTTACTTGGCTATGTTATTGGAAATTTTATTAGGAATGTGGAGCAGAACAG
TGTATGTGCCACGGTCAGAAACTTCAGGGACTGAGACACCAGGACCAGATTTTTGGT
CATGGACACCTCCTCAAGGTAGTGAAATTAGTTCTGTGGACTTGCAGGCTGTGGAAA
AGCCTGCTGAGTTTCCAACTTTGCCAAATCCTGTATTGGAGAAAGATAAATCAGCGG
ATTCTCTTTCGATACCATATGAGAGTATGCTTTCTTCTGAAAGACATAGCTTTACTAT
CCCGCCTTTTGAGTCTTTGATTGAGGTTCGAAAAGAGGCTGAGACGAAGCCTAGCTC
CGAGACTTTATCGACAGAACATGACCTTGATCTCATATCTTCAGCAAACGCGGAAGA
AGTAGCTCGTGTTCTTGATAGTTTGGATGAATCTTCAACGCATGGAGTTAGCGAAGA
TGGATTGAAGTGGTGGAAGCAAACGGGTGTGGAGAAAAGACCTGATGGTGTGGTTT
GCAGGTGGACAATGATACGTGGGGTTACTGCTGATGGTGTTGTTGAGTGGCAAGAT
AAGTATTGGGAGGCTTCTGATGATTTTGGGTTCAAGGAACTTGGTTCTGAGAAATCA
GGACGTGATGCCACTGGAAACGTGTGGCGTGAGTTCTGGAGAGAGTCAATGAGCCA
GGTTAGTGTAAAGTAGTTGCCGTAGTAAATCTAACGCACTATAGACTATTTGAGACT
TAATTTGAATTGTCTATTACAGGAGAATGGTGTTGTGCATATGGAGAAAACTGCAGA
CAAATGGGGAAAGAGTGGACAAGGTGATGAATGGCAAGAGAAATGGTGGGAGCAT
TACGATGCTACCGGAAAATCAGAAAAATGGGCTCATAAGTGGTGCAGCATTGACCG
CAACACGCCTCTTGACGCTGGCCACGCTCATGTCTGGCACGAGAGGTATTCTCAAAA
TCCTCTTTTCACATAACGAAACACTAATAACATAATCGAATCTTTATCCTTTAACTTT
CAGTCCTTAAAATATCAATCAATCTCTTTAACACAAGTTAGAATGTTTGTCCTTTAAC
TTTTACACCTTATATTGTTGAACATCAAATCAGTTCAGTAGCAAAACATTGCCTTTTC
ATACTCTTTAAGGTGCTCTATTAACATTTAGAAGCTTAGTCTGCGAGCTTTGTTGAAC
TGTTATCAAAACAGAACTGACACTAGACAAGCTGTGACCAAATAACAGGGAATGTA
GAGTGTTTCTAAATATGTCTTGATTGGTTTTGACCAGGTGGGGAGAGAAGTATGACG
GGCAAGGCGGAAGCACAAAGTACACAGACAAGTGGGCGGAACGGTGGGTAGGTGA
CGGTTGGGACAAATGGGGAGACAAATGGGACGAGAACTTTAACCCGAGCGCTCAAG
GAGTGAAACAAGGTGAGACTTGGTGGGAAGGGAAGCACGGCGACAGATGGAACCG
AAGCTGGGGAGAAGGTCACAACGGATCAGGATGGGTTCACAAATACGGAAAAAGC
AGCAGCGGTGAACACTGGGACACACATGTACCACAAGAAACTTGGTATGAGAAGTT
CCCTCACTTTGGCTTCTTCCACTGTTTTGACAACTCTGTTCAGCTCCGAGCCGTTAAG
AAGCCTTCTGATATGTCCTAGATATATCAAATACTAAGCATAAAATATATAAATAAG
CTGGGACAATCAAAGAATTGGCCTACAATATTAGAGTAGTTTGATCAAGTGCAATGT
ATGTAATATTTATTATGACTTCTAATTTGTTCTATGCTTTGGTGTTTCTTAATGAGTAT
GGCCAAAGTTTATTTGGTAGTTACTAATATAACACAAATCTGATTAGAAAACTGGAA
CACATTCCATTCCAGGAGCATAAAGTTATATGAACAGATAATCTAAACTCTTTATTT
GGTAAAGATAAGAAGACAAATATTTCTGTAACCAATAATAATCTTCAGTTACATTT

GTATTAAATTCTAATAAAAAAAAATATACAGAGGACAAGCCAAAGTGGAACCCCACC
CACTGTCTTCTCAAAGATTAAAAAAAAGAAAAGGATTTACCCAAAATCTCCGATAT
TCTTATTCCTTTGCTGTATCATCTTCTTCTTCTACTTCGTCCTCATCATCGTCACATAG
ATCCTCCTTTTTCGTTAATTGGGGAGAGATATACGTAACAGCTCTTCTCTCTACAAAG
AAGTTGACTTTTGCATCCTTACATCTCTCAAATCTTTCTCTGGTTAATCTGTATTCGTG
TATTTTTGCAGTTTCTTTTGAGGATCAGATCGTGACAGGGTTTAGCTCAAGGAGAGG
TATTAGATCCGTACTTTGTGATAAACTTTCTCAAGGTTTACGATCTCATTCTATCTAG
ATCGGCTTCGTATAGATACAATCTTGTTTCAATTAGCAATGGATCGATTTTGTTATTC
GAATATTTAGAAAAAGGAAAGATTTTTCCAAAGGGGTTTCCTCTTATACCATTTTTTC
TGGGTTTTTTGAAATTTAGGGTGAGAAAAAGAAGAAGAAGGTGAGAAAAGTGAAGG
CAAGGCTTTGAGGATGTCTTTTACAGGAACTCAACAGAAATGCAAGGCTTGTGAGA
AGACTGTTTATGCTGTTGAGCTTCTCTCTGCTGATGGAGTTGGATATCACAAGTCTTG
CTTCAAATGCACTCACTGCAAAAGCAGGCTTCAGGTCTGATTCTCTCATCTTTGTATT
CATGTTAACGATGCTGCTTTAATTAGAGGAATGAGTTTACGAATTACCCATCTTCTTG
CTTAAATGATGAATTGAATGTCTATTGTAACCATAAGCAGCTCTTGATAAAGTAAAT
GTCTTTTTACAGCTGAGTAGTTACTCATCAATGGAAGGTGTTTTGTACTGTAAGCCTC
ATTTTGAGCAGCTCTTTAAGGAGAGTGGTAGTTTCAACAAGAACTTTCAGTCACGTA
TGTAGTTTTGTTTCTGCTTCGATTCATGTTTTTTTTCCGCTTGCCCTTCTTTTGGTTATT
GATTTAATCAGTCTTGCAACTTTGTTTCCTCACAGCTGCAAAATCGGCTGACAAATC
AACTCCTGAGCTGGTAAATGTTTCTTTTCTGGTTTGGTCAGATGCTCTTTGGATACAT
TTTCTGATTGAACTTTTCTCTTATATTTCCTTCTTGGTTTGTTTTCAGACAAGGACGCC
TAGCCGAGTTGCTGGCAGGTTCTCTGGTACACAAGAGAAATGCGCCACTTGTAGTAA
AACTGTGTATCCTATTGAAAAGGTTATAACTCGTTTAATCTTTCATTGCTCTTCCTTC
ACTCATGCATTTTCCATTAACTGAAGCTTTGGAATCACCTTTCTGCACTCTAAGGTCT
ATGTTGGAGTATTACTCTAAAACACAGTTCCATCATTTGCAAGAATTCTCCTGTGTTT
TCTATTTCATGTTGAGGATAGATACATAATCCCCTCAGTTACCGAGAACTTGCAAGG
AAACCAAATGTTTTGCACAGATGCATAGACCCTGGAGATATCGGTTCTTGTTACTTT
AATTTGCATGTTAAACCTATAAAGGAGACTTAATATGAAAACTCTTTATGGGATTTT
TAAAAGCTATTTGGTCACAAATATCCGTCTTTTTCGATTCTGAAATATGCAGGTAAC
AGTCGAGAGCCAGACATATCACAAGTCCTGCTTCAAGTGCTCACATGGAGGTTGCCC
AATTTCACCTTCCAACTACGCAGCTCTTGAAGGAATCCTGTACTGCAAGCACCATTT
CGCTCAGCTCTTCAAGGAGAAGGGAAGTTACAACCACTTAATCAAATCCGCTTCCAT
CAAACGCTCTGCAGCCGCAGCAGTCGCCGCCGGTGTACCAGCAGCCTCCGTTCCTGA
ATCTTAAAATCCAATAATTTCTCCTCTCTAGTCTAAAATTTGAAGTTAATGCCAAACT
GATTCAGTTTGATTTCTCTTTGTGAGAGCGTGTGTGTAATCTATGCTTTGATGTTCTT
ATTTCCATTGATGTCTCCTAAAATTCTATGTTTGTGTGGTGATTTGGTTATCTATAAA
AAGTCTGAAACTTTCCTTATAAATCTTCAATTTCGTTT

>AtPLIM2a_2000bp

GTTTTAACTTTAGAGAAAGTTATGTTCAGGGAAAAACTAGAAATGTCCTTTTGGGCA
AAAATCCAACAATGTTTGTTAATAAGAAAAATCTAGCTCAGGGGTAATAGAATTAG
GAACTCACCTCATCCATTACAAGCATGGCACAATCTTTCAAAACACACACACCCTTT
TTGGCAAGATCTAATATTCTTCCAGGAGTTCCAACCAGTAAATGGACAGGTTGATAC

AATCGCATGATATCATCCCTCAGACTGGTACCGCCAGTGGTGACCATAACCTCAATT
TTCAAATATTTGGAAAGCTCCTTGCAAACCTGGGATGTCTGAAGGGCGAGCTCTCGG
GTTGGAACTAGAATTACGGCTGCATAAGAAAAAAGATCAACAAAAGTACTGAAAAC
TCAGTAAGAAAGTAGGGTTTCACTGGAAGTTCCACTGATCAACTAACTCACTACAGA
GAAAGGTAGGAGATCTTTTAACAGAGAAAAGTTCTAAAAGGAAAGTAAAAAACCTT
GAATGACATTGTTCTCTGGATCAATTTTCTCAAGGGTAGGAATGCAGAAGGCACCAG
TCTTCCCAGTACCATTTTTGGCTCTAGCAAGAATATCACTACCAGTCAAAGCAATGG
GAATGCTTTCTTCCTGAATAGGAGATGGTTTTTCAAAACCCTTCTCATATATTCCCCT
AAGCAGATCCCTCTTCAAAAAGTAATCTTCAAATTCATTTCCTTTAGTGGCAGTCAC
ATCCTGAAGACAAGATATGGAAAAACTAAGTTCTAAGGGAACTGCTGTCTTGTCAA
AACAAAAATCATATAACTATGCAATCATTCATACCTCTGTCTGGTAACGATTATCTC
GTGGTGGCAGCTTTAAGGTTGCTTTCCAGTCTTCATTACTGATAAGCATAAGAAAAC
ACATAACCCGTTAAACATCACAACAAGAGCACCATTCATAATAACCATTCTTCCAAA
CGAGCACTTACTTAGAATCGCTAATAGCTTCAGATTGAACCGTTTTTTCGACCTCATC
TATGTAACTTGGATCACCAGGAAGCTGAGCCCGTCGAGACCATTGTTGTTCTTGCTG
CTGCTGTTGTTGTTGTTGTTGCTGCTGCAGTTGCTGTTGCTGTTGAATCTGCTGGGGA
TAACCTCGCTGAACGTACTGCTGAGCATCAGATTGAGACTGGAGATACTGAGGAGG
CTGAGGCTGAGGTTGCTGTGGAAACGGAGTCCGTGACTGAAGGTACTGTTGAGGCT
GAGGTGGATTCGGATTCCGTGACTGAAAATTAGGATCCGGACCAGGACCCGCCGCA
CCAATCCCCGGTGGAAATCTTCCTCTATTATTATTATTATTATTCATCTCGATCTCGA
AACAGAGAACTCTAATTCTACCAAGAAATCTTCACAAGCTCACAAATCGTAAATCTA
AGAAGAGGCTATAAACAACAATCTGGAGAATGTTAGGTCACACGTAGCTTCGTAAG
AGATGATTGATTGATTGATTGATTGGACTCTCTCTCTCGGGGGAGGGCAAGATCGGA
ATATGCTAACAATTATCCACAAAACCGTTTCCGCCAGAAACTAATCGAGCTTAGCTA
AGATGAATAATTTTCTTTTGTCGTCTGGTTGGTTAGTGAGAAAGTGAAAGATACATA
CACTCGCCGGAGTCGTTGTTGTTGTTTAGGTCGTCAAGTGGATTTTCATTACACGGAC
AGGCTCTTCTTCAGTTTCACTCACTCTTATCCACATTTATTTCACTAAATTAGCCTTGT
ACGTTCGTATAATTATGAAATAACCCCCACATTTCTTTGTATTTATCATTATTCTCTC
GTAATTTTTAAAAATAGATCTTCGGATCTTCCGCAAACAGAGCGTACGGCTGAAAAC
GTCGATACCCTTCATTGTGCTCGGAGTCTAAAATTGTAAAATTGAAATTAGAATTTT
GATGATCGACAATGATTGAATGGCTTCTCCTCTTTCGAATCTCTACCACTAGAACCA
AAATTACCACAAATTCTCATTTCTTCAAGAATTTTTGATAAATAAGACAGAGAACTT
GAGAGATGAAAAGAAAAAAACACAAAACCTGGTGTTTTTTCTCTCGTCGTCGTTTGG
TCGTGTCCTAAGACAAGAACCGGCGAACGAAGAACAAACTTTTTGAATCTAACATC
CGAAAGAAAGAGAGAAAATGTCGTTTACAGGAACATTGGATAAATGCAAGGCCTGT
GACAAAACCGTGTACGTGATGGATTTGTTAACATTAGAGGGTAATACTTATCACAAA
TCATGTTTCAGATGCACCCATTGCAAAGGCACTCTCGTGGTATTTCTCTTTTCTTCTT
CTTCCTCCATTGTTACTCTGTTTTTCGACAATGCTTCATTGTTTTAAAAGACCACATCT
TTTCTTAACACAGATAAGCAATTACTCATCAATGGATGGAGTTCTTTACTGTAAGCC
ACACTTCGAACAGCTTTTCAAAGAATCTGGCAATTACAGCAAGAATTTTCAGGCAGG
AAAGACCGAGAAGCCCAATGATCATCTGGTAATGTCTTCAAAGATTCATCATCTTGT
TTTTATTTCCTCTTTTTTTTCATCTGATTTGTCTCAATTGATGGATACTGCAAGACTCG
AACTCCAAGCAAGTTATCATCATTCTTCAGTGGAACACAAGACAAATGTGCGACTTG

TAAGAAGACGGTTTACCCACTTGAGAAAGTAACAATGGAAGGAGAAAGTTACCACA
AGACTTGCTTCAGGTGCACACACAGTGGTTGTCCTTTGACTCACTCTTCTTACGCTTC
TCTTAATGGCGTCCTCTACTGTAAAGTCCACTTCAATCAGCTCTTTCTCGAGAAAGGC
AGTTACAATCACGTCCATCAAGCCGCTGCTAACCACCGTCGATCCGCCTCTTCTGGT
GGCGCTTCTCCTCCTTCTGATGATCACAAACCTGACGACACCGCCTCAATTCCCGAA
GCAAAAGAAGATGACGCTGCCCCTGAAGCTGCAGGAGAAGAGGAGCCTGAGCCGG
TCGTTGAGTCTTGAAAATGTCGTGGAGGAATGATCTTATCAGTTCTCAAGTTTGAAG
TTTTTATATGCGTTTCTGTTTAATGAATATATAAATACAAATTGTTGTTGTTTCTTCTT
GTAAATTCTTGTAAAGTTTTAAAGCTCTTTTTGGAGAGAATCTGTTGTAAACAAAAT
CTTTAACATCCAATTTTTTTTGAAGAGGATATTTTTAACCTTTAGTTTTAAAA

>AtPLIM2b_2000bp

ATGATGTAGTAGCATTTTTTGGCCCCATCGTCGCTTGAAATGACTCCTTATGGTGCCT
TAAATTGGAGTTCATTTCTTATCAAGTGTATGTACGTATTTTACTTGTGTTTTTTCCTT
CTTTCTTTCAGGTTCGATCTTGGATGGAAGAAACCATTCCTGGTGTTAACCATTGTAT
TCTTTCTTATGTGATTGGGGTTGATAGCTTGAAGGCGACAAGCAACGGCACTGAATC
TTGGCAGTCATGTGGAGATATTAGATTACGTATGGATGGTCTTTTTAAACTGAAGGA
ACATGCGGTCCAGTTAACAAAAGAGTTTACTGCTCTATATACAAACGGACCAGCTGG
TGGTGGTGGAATTAGGTAATTTCTTCACGTTGATGTTCGCTCCATATATCAATCCAAT
GCTAATTGTAGCTTTACAGTAGTACCAAATCCTGTTGAGTTGAGACATCTTATGACT
AGGGACAATGTATTGAAACTAGTTATTTGTTATGAGATTCGGGGTGGTCTTTTTGGTT
TGACAAAACTGCTTGTAAATTATATGCAGTACCGGACACAAGATGGAAATTGTTCTT
GAAAAGCGTTTGGTAGGTCGTCTCCATCAAAAAATTTAAACTAATATAGCCTTGCAT
CAATCAATTTTACGGATTCTGATGTTATATGTTTGGTTATCCAGGTCAGCCGTGAATC
TGTAATGTGGAAAACTGGACTTCAGCACACCAACACTTCAGAGCCAGAGACCTCTG
AACATCATTCTCCAGAGAAAATGCCAAAGGTTAGCTCGAGAGTTCTTACTAAACTAA
TTGTGATGACTGATAGATAATGGAAATAAACATGACCTGAATTCAAATTCCTTGGTG
AATCAATCCTAGTGTGTTCGAACATCAAACTAACTCTGATGACTGATAGATATGTTT
TACTATTTGAAAGCTGAAAATGTGAAAGAACTCTAACATGTGCATTTGTATTAACAC
CTTGTTATTACTTCCATTTGTAGTTACCTAAGGAAAATCCAAAGAACCTTACAATGA
GAGGCTATCAATCTGGATTTCATCACTCTCCTGCTCCATCTGGCCAAAAGATCCCAC
TCTACTCTGTTGCACATAGCCGGGCTGGCGATAAAGGAAACGACATAAACTTCTCAA
TAATCCCGCATTATTCCCCAGATGTCGAGCGTCTGAAACTCATAATTACTCCTCAAT
GGGTAAAGCATGTGATGTCAGTTCTGTTATCAACCTCTTCGTTTCTGGAATTGGATGC
AAAACCAATGGATGAAAATGTGTCAGTAGAGATATATGACGTTGAAGGCATTCATG
CTATGAATGTTGTCGTACGGAACATATTAGATGGTGGAGTCAACTGTTCCAGGAGGA
TCGACCGACACGGAAAGACAATCTCAGATCTCATCTTGTGTCAACAAGTTGTGTTGT
AAGACTACTTTAACTTTGCATATCAGATTTTTCTATTCAAATTCTGTGGTAATATGTT
AATAAAAAGGGAGACTTGAACTACACTATAGTTCTAAAAGATAATAGAAAAAGAAG
GAATCGACTTAGTGGTGCTTCACTTTCTTTTACTTCTATACATTAAAATTACTTTAAT
TTACAACTGGATATCTTGATAGATACGCATACAGATAAGTATTAAAAGCATTGTTTT
GATTGAGAGAGAATGATATTATATATTTGTGTAATGTTAGGTTGTTGTTATATTCTTT
TGCAGGATCTGACTGACATGGGTTGCACCACCTCAGAGACAGTTGGCTTAAAGTAAC

AATCCCTTATTGGATTGAAATTGTCAGAAACCACAACCCTTCTTTGCTCAACTCCAA
TCACTTTGCCACCCAATTTTATCTCATTCATTTTGCAAAAAAGATCAAAGAGAGAGA
GAGAGAGAGAGAGAAAGAAAAAAAGGAAAAAACCTTCCTCCGTTACAACCTCACA
ATTGCAACAAAGTTTCATATAAAAGAGGACAGAAATTTCGCAGGTTCCTCCGTTGTC
TGAGGTAAAGGTTTCCTTCTTTAACTAATTCCTCAGATGACCTCTTGATTTGTTGGAA
AAAAAATTAAAAAATCTGTTTCATACAGGATCTTGGAGAAGGAAGGAAACAACAAC
AACCAAAAAAAAACCAAAAAAAAAAAAGATAAAACAAAAAAAATGTCTTTCACAG
GAACTCTCGACAAATGCAATGTTTGTGATAAGACAGTCTATGTGGTGGACATGTTGT
CCATTGAAGGAATGCCTTACCACAAGTCTTGCTTCAGGTGTACCCATTGCAAAGGAA
CCCTTCAGGTGCACTCATTACTCATTTTCTTGTTAGATCTTCTATGATCATATTATATC
AACAATTTATCTATGTGTTGATGGCAGATGAGCAACTATTCCTCCATGGACGGAGTT
TTGTACTGCAAGACTCATTTTGAGCAACTCTTCAAGGAATCTGGCAATTTCAGCAAA
AACTTTCAACCAGGTTCGTATTATAAAATCTCTGACATCGAATCTCTCTATCTTTCAA
ATCATCATGATCATAACAAGTCTCTTTCTTTTCTCAGGAAAAACTGAGAAGCCAGAG
CTGGTAGGTTCATCATTACATGTCCTTGTATATGATTAGCGTGTCAATATTTCATTTT
CTTTCTTTTATGATCGACTAATAGGATCTGTGTACTGATTTAAACATTACAGACTAGG
ACTCCCAGCAAGATATCTTCCATCTTCTGTGGAACACAAGACAAGTGTGCCGCTTGC
GAAAAAACTGTTTACCCTCTTGAAAAGGTTAGTGGTATACGCATCTGCAAATGTTCT
CATGGCCTAAAATACCAACGAACTAGTCTCGTAGAGAAATTTATTTGCTCTAGAATT
TGTTTTTTGTTTTTTCATTTCCGTAGAATTTTTCTGTTCGGATAAAATCATTACTAGAT
CAAATGGAAATAAAATTTTGATTTTGGTTTGGTTTAGTATACTTTTGGTTCGATTTGA
GAACGTGATGTTGGTTGGTCCAAGCAAATGTGGAATCAATTTTGGCTTGGTTCTAAT
CGGGTTATTTGGTAACAGATACAAATGGAAGGAGAATGCTTCCACAAGACATGTTTC
CGGTGCGCTCACGGTGGGTGTACGCTGACTCACTCCTCCTACGCCTCCTTAGATAGC
GTTCTCTATTGCCGACATCACTTTAACCAACTCTTCATGGAGAAAGGAAACTACGCT
CACGTCCTCCAAGCCGCCAATCACCGTCGCACAGCCTCAGGCAACACTCTTCCACCG
GAACCTACCGAAGACGTCGCCGTGGAGGCCAAGGAAGAGAATGGCGTTTCAGAGTC
TTGAATATCTCTTACATGAGTGTTTTTGAGTGAATGATTGAATGAATGTTGTTGCGGG
GGAAAGATTTGATTTCATTTGTAATGAGTTGTTTCCATATGAAGACATGATTTTTTTT
GTTATTTTTTTAATTCATTCGTTATACATTTATTATTACACACGAAAAAAA

>AtPLIM2c_2000bp

TGGAAAGCTCCTTGCAAACTTGTGATGTCTGAAGGGCCAGCTCTCGCGTTGGAACTA
GAATCATGGCTGCATATAAAAAATAGAACAAGTGGTTCAAGGACAAACACAAACAG
TCAACTGATCACTTATCAACTCAACAGGGAAAAAGAAGTATACCTTGAATAACATTG
TTATTTGGGTCAATTTTCTCGAGGACTGGAATGCAGAAGGCACCAGTCTTTCCTGTA
CCGTTTTTAGCTCTAGCAAGAATATCACTACCAGTTAAAGCAATTGGAATGCTCTCT
TCTTGAATTGGAGATGGCTTCTCAAAACCCTTCTCATATATTCCCTTTAACAGATCTC
TTTTCAGAAAGTAATCTTCGAATTCATTTCCCTTTGTAGCTGTCACATCCTGCAGACA
ATATGGAAGACATTGCTCAACACGAGTTTATTCAATATCCTCAATATTCATAATTCCT
ACACAGAAAAACTCAGAAGAAAGCAGCCATACCGCTGTCTGATAACGAGTATCAGG
AGGTGGTAGCCTTAACGTAGCCTTCCAGTCTTGACCACTGAGAGTAACAGAGAATCC
ATGAGCACACTAAAGAGATATAACTTAACCTCAAAATAACACATACATCTCTAACA

GCACAAATCATTACTCAATGCAAACAAAATCATTCATAATAATTACTCCAGCATCAT
AATTTGCAACAAGACGTATTCAGGAACAAGAACTTACTTAGCATCGCTGCTAGCTTC
GGGCTGGGTCGTCTGCTGAACCACCTCATTAGCGTTACTAGCGTTTCCAGGAAGCTG
AGGGCGTCTTGACCACTGTTGCTGCTGCTGTTGTTGTTGATGTTGTTGCTGGAGTTGC
ATCTGCTGAGGGTTCTGAGAATAACCGCGTTGAACATACTGCTGATCTTGAGGTGGT
TGTTGCTGCCGATACGACTGATGATAATCTGGATTCGGAGGCGCACCACGACCCGTT
CCAACACCCGGTGGATATCTTCCTCTGTTAGTATTCATCACAAATCTCTCTCGATCTC
GAAAACTAGACACAGACAAACCCTAAATCTACCGAAATACCTTTTACAAAACTACA
AAGAATAGAACGAAAGAGTCTGCGATAATCTGGAGAAACAGAATGCTTGTGTAAAA
CCTAGGGTTTAAAGAAACTGATCGTGGAACTCAGGGCAAGATCGGGAATGTTCAAA
TAAAAAACCACAAAACCGGTCCGCCACAGAAGAACGGAGAAGAAAGCGAATCGAG
AGTTCCGATTGGAATTACAACAATCTAAAGAAGAAGAAGAAGAAGAACGAAA
GAGAGATTAAGAAAAAATTTCGTTTGAGTGGGTTGGGTTACAGGAAACTAGAGAAA
GTGACGAATACACTCGCCGGCGTCTTTTGTTAGGGCGTCAATGGATTTTCTCCTCTTT
CTTTTTTTCAGACTCTCACTATTTATCAAAATGGCCTTTTATGTTTACGTTAAATAAA
TACATGACCCATAAGCTTTATATTATTTACAGTATAACTCGGATTTACTTTTCTTTCA
GGTATTCACCCCATGTTGTATTGTTTTTGTCATTTTAGACCCCCAGATTCTTTGCCCTT
TGATTAATGATATCTAACAAAACAAATTATGCTTATGATTATTAAAAGCTATGCTTA
AAAGGCAGTCTTGTGATGTTAGATTAGGTGTTACTTATTTGTATAAAGATCCTTATA
ATTGTGTATATGAATTAATAACATCTTAAAATCGGAGGCTAACTATATATAAACTTT
GCACCTAATCCCTTGGTTTACACGATATAATTTAGAAAGCAAAACAAAAATAAGTGT
TAAATAAAATAAAATATAGCCAGAAATAGATTTAGAATGTAGAAGAACATTTTAAG
GGCTCTCCTCATTTGAATCTCAACCATCAGAAGAAAATTTTACCCCTAAAAATCTCA
ATCCATGATTTTGATATAATAAAAAGGCAGAGAGCTCATAGATGATGGCAAAACAA
ACCTTGATTCGTGTCTAAACGAACAACAAAAAGAAGAAGAAAACTTTTGATCCATCT
TCCAACTCTTTCGTAATTTGAAAGAAGAAGAAGAAGAGATACCAATGGCGGCGTTT
ACAGGGACAACAGACAAATGCAAGGCGTGTGACAAGACGGTATACGTTATGGACTT
GATGACTTTGGAAGGAATGCCTTATCACAAGTCATGCTTCAGGTGCAGCCATTGCAA
TGGCACTCTCGTGGTATCTATCTATCTTCCTTTTCCTCCATTGTTCCCTCATGAAAAA
AAAAACACAAAGAGAACATCTTTCTTCTCCTTTTCAGATATGTAACTACTCATCCAT
GGATGGAGTTTTGTATTGCAAGACCCATTTTGAACAGCTCTTCAAAGAATCTGGAAA
TTTCAGCAAGAATTTCCAAACAGGTTGCTAACAAATCCTCTGTTTCTTTGCTCTGTT
TTTTATTAACCTCTCTTGAAAAGCTAACAATCTTTGCTACTTTCTCTTCTTGGTCTTTT
ACAGCTGGAAAGACCGAGAAATCGAATGATGCGGTAAGCACATAATTCATCAACAT
TCCTCTGTTTTCTCTTTTTGTTTTCTAATCTGATTTCTTGGTGTGATCATTTGCATAGA
CGAAGGCTCCAAACAGGTTATCATCCTTCTTTAGCGGCACACAAGACAAATGTGCAG
CTTGTAAGAAAACAGTTTATCCTCTAGAGAAGATGACAATGGAAGGAGAATCTTAC
CACAAGACTTGCTTCAGATGTGCACATAGTGGTTGTCCATTAACACATTCTTCATAT
GCTGCTCTCGATGGCGTCCTTTACTGTAAGGTCCACTTCAGCCAGCTTTTCCTTGAGA
AAGGTAATTACAATCATGTCCTCCAAGCCGCGGCTAACCACCGTCGCTCGACGGCTG
AGGAAGACAAAACCGAACCCAAAGAAGATGAAGCAAACCCTACAGAAGAAGAAAC
TTCTGATGCAGCAGCTGAAGAACATGAATCCTAAGAAATATCCGGGAGTTCCTTTTG
TGAAATATATATAATCATAAATTGTTAAAGAGATAATTTCTTAACTAATGCAATGAA

GTAGACTCAAATTTTGTTTAGTTTCTTCTTATGGAACTATATATTATTCTGTTTGTAA
AGTAATTATATTCAAGTTTATGAGTAATTACCAAAACA
