# Supplementary material for: A genome‑wide approach to the systematic and comprehensive analysis of LIM gene family in sorghum (Sorghum bicolor L.)
Source: Genomics Inform. 2023 Sep 27;21(3):e36. doi: 10.5808/gi.23007 (PMC10584642; doi:10.5808/gi.23007)
Supplement: Supplementary Fig. 5. — Full-length genomic sequences of LIM gene families of Sorghum bicolor (Doc). [file gi-23007-Supplementary-Fig-5.pdf]

Supplementary Fig. 5. Full-length genomic sequences of LIM gene families of *Sorghum bicolor* (Doc).

>SbLIM1

CAGCACGGCACTGTGCTGTGCCTCCATCATCATCATCCGATTCCCACATCCCTCGTC
GTCGCTTGTTTGTTGCCGCTGCTGCTGCCTTTTCCTTTCCGCCGCGGCAAAAGTGAGG
CGCGGGCGGGCGAGCTGGGGGAGAGGGAGACGAACCCATCATAATTCCATCGGTGC
TCCTGCGTATATAAGCGCGGCAATCGGGACGCCGGGCGAGGAGGTGTTCATTCCTCA
TCCATCCCACGCCACGCGCGCGCAGGCCAAGGCCGGATCGGGCTCTCAGTCAGATC
TCGCCCGGCGGCCACGACCCGAGAGAGAGAGAGAGAGAAAGAGGGGGGTTGGCCG
GCGATCGGGAGAAGATGTTTAGCGGGACGCAGCAGAAGTGCAAGGTGTGCACCAAG
ACGGTGTACCCGATGGACCAGCTCTCCACCGACGGCGTCGCCTTCCACCGCTCCTGC
TTCAAGTGCCAGCACTGCAAGTCCACCCTCTCCGTACGTTCCCTTGCGCATGTTCTCT
TGTGATGGGTAGGTGGATGGATCTGTAGGGGCAATGCCATGTAGCATCGGGTAGTA
GATCTGCGAATGGTCGCCGCGAACGGATCTGGTGCGAGTTTCCTAGTATCAGGAAA
ATTCTGTATGCGCAGAAGAGATCATGATTTTGTCCGTTTCCCATTTGCTAACTTGGTA
AAACAGAGAGGATTAGGCAGGAAATCGATGCAAATGAAAACTCCATTTTGATCTTC
TGTTTAGGACGACCTGTCGATGTATGTCTAGGCAAAGATAAGCAGTTGTGTAAGGTC
TGACAGCCTCCGTCTTCATCCTTTTCGCCTTCGATAGCTGAGCAACTATTCCTCGTTC
GAAGGAGTGCCGTACTGCAAGGCCCATTTCGAGCAGCTGTTCAAGGAGACCGGGAG
TTACAACAAGAGCTTCCAATCACAATCACGTACGCTTTCATTCAGTACCGAGCATTG
ACATGAAAATACAAGTTTTTGTGTTCCTGTGATCTGATGCAGTGATGTGCTCGTGGTT
TTCTTCTTTCAGCCGCAAAGATTACTCCGGAAAAGTTGGCCCCTGAGCTGGTGAGTG
CTAGTCTGTACGCTTTAGTCTTTGTCTCATTGTATTCTTGAAATGGAGAAGTAAACAG
TTTCGTTCAAAATCTGCTATCCAGACCAGATCACCAAGCAAAGCTGCAAGGATGTTT
TCAGGAACACAAGACAAGTGTGCGACTTGCGGTAAAACCGCATATCCTCTTGAGAA
GGTAATTTTGATCACATTATGCATTTTTACTTTTCATTATTTCCTTTCAATTTTTTGGG
ATGTAGCTTGTGAAACTCTGGTATCTAATTCAGTAGTTCCTCAAGGTCAATATGTGCT
ATGGAAGATGTGGGTATCAATGGCTTTCACAACTGAACAAATATTTGTCCCCTTCTA
GTCATGTTAACCTGGTCGCGCTGTCAACCTAAAATTTGCTCCATTTAACAATAAAAT
TATATCTTTGTTCATCTTAATTTGTCATTATCCTTTCTTGATTGCCTTGAATCATCAAA
ACTCTTTCTGATTGTTTTCCTTGAGCCAACAAAACATGTGGACAGTGGTGGTTCTAAT
GCATGGCTGTTAAAAACATTGCAGGTAACAGTTGAAGAAAAGGCATACCATAAGTC
ATGCTTCAAATGCTCCCACGGGGGCTGTGCGATTACACCTTCCAACTATGCAGCCTT
GGAGGGCATCCTCTACTGCAAACACCATTTCTCTCAACTTTTCAAGGAGAAGGGAAG
CTACAACCACTTGATCAAGTGTGCTTCGGTCAAGCGCGCTGCTGAAGCACAGCCAG
AACAACCAGCCTCCGATTCCTCCTGATCGTGCCATTTCAACAGTGGGGGTTTGAAGG
CGAGAGTTGGGAAGCCTCCTGTTTTTACTTAAAAACGCATGCTGTATGTCATTCATCT
ATGCCCTGAGGATACCGGACTATCTATTTTGGTATCCTTTGCTGTGCTAGCTGGATCA
CATCGGTGAACCATGGAACTGATGTCATTTCCAATGAAATTGTTGTGCATCAAGTAG
TGAAATCTTCACTGGAATTGTTGTGCAAGTAATGAAATCGGGTATCTTGCCCTTATT
GATCGATAGTCCTATGTTGTTTTGTTGTCTT

>SbLIM2

GGAAATAGGATAGGAGAAAGGCGCCGGGACCGGGACAGACAGACCGCGGAGACAA
ATCAATCTTTGCCTGAATTTGTGTGTGCGGCTTAGAAGCCCTCGCGCCTCCTATCTTT
GCTCCCGAGCTCACGCGCTGTGTCCTTTAAAGCGCATCCGTCTCGCTCCCGGAGACG
TCAACCTCTGCCTTGTTCCTTACCGGCCCCCGAATCGCGAGCGAGGCGAGAGTTCCT
CTCCTCCGTCCCGCGCCCGTTTGCTCGTCGATCGATCGGTTGTTGCCTGCCGGCCGCG
AGCACGAGGAGCGGCTAGCCAGACGGACGTGTCGTCTTGGCAGGTGCAGCAAGCGC
GGAGCTCGATCGCCGCCATGTCTTTCACCGGCACGCAGGACAAGTGCAAAACCTGC
GACAAGACGGTCCACTTCATCGACCTCCTCACCGCCGACGGCGTCTCGTACCACAAG
ACATGCTTCAAGTGCAGCCACTGCAAGGGCACACTCTCGGTACGCAACGCATATAT
AATGCGCTGCTCATTTCCGTCGAAAGAAATGAAGCTTTGTGTTATTTGATTGATTGAT
CTCGACAGATGAAAGTGTAGAAATGTGGTGGGCTCATCATTTTCATTTCTGCATGCG
TTCTCCTCTAGATTAGCAGCTACTCTTCCATGGACGGTGTCCTGTACTGCAAGACGC
ACTTTGAACAGCTCTTCAAGGAGACAGGGACCTTCTCCAAGAAATTTCAAGGTAATC
TACAGCGCATCAACTTGTCTATGCTGTAACGTTCAACTCTGGTCCTCTGATTCAATAT
ACCGGGACGTTTTGTACCTGAACCTGTAAAAATGTTTACTGCACATTTGTCATCTTCT
GAATGTTACACACATCAAACATTCTGATCCTTCCGTTGTGCTACACAGGTGGAGCAT
CTTCAACCAAGACCGACCAGGTACGGTTCCTCAAATATTCTAAACAGATGATACAAT
CTGACATCAGTAAGAGTTGACTAGTCATGCACGATGCCAATTCGAAAATCTCTGCAG
GCAAAGGCTCCGAGCAAGCTATCATCTGCATTCTCTGGAACTCAAGATAAATGCGCA
GCCTGCCAGAAAACCGTGTATCCATTGGAGAAGGTACAGAAATGCTGTTTATTTCTG
TAACTCTGGGCGGCAAACAGTAGCACACAATTATCTGATCAAATCAATCACCACGA
CTAATGCTAATGTTGGCACCTACGCTTGATCTTCTTGTTTAGATGACGTTGGAAGGC
GAGTCTTACCACAAGAGCTGCTTCAAGTGCTCGCACGGGGGCTGCATCCTGACAACC
TCCTCCTACGCCGCGCTCAATGGGATCCTCTACTGCAAGATCCACTTCTCGCAGCTG
TTCAAGGAGAAGGGCAGCTACAACCACCTCATCCAGACGGCGCAGACCAAGAAGA
ACGAGGCTGCGGAGGCCGCACCGGAGGCACCGGCGGATGCAGGCGCGGCTGAGCC
AGAAGCAGCGTAGATAGCAGCAAACGCAAAGAAAAATAGAGCCGTGTGTGTGTGTG
TGTGTGTGTGTCTATCTCCTATATATACGAACTCTGCATTCAGTAAGTAGTTGTCACA
AATATATGTTGTTGTTGTTATGTTTACCTTGCTCTGGCTTATTCTGTATTATGAGGCTC
TGGATTCATGACGATGAAATGAGAAT

>SbLIM3

GAGAGAAACGATTGCGGATTGCATTGGGGGCGACGAGTGCGTGCGTGCAGCCACAG
GCGTCGGCACCATGTCTTTCACCGGCACGCAGGACAAGTGCACGGCGTGCGACAAG
ACCGTCCATTTCATCGACCTCCTCACGGCCGACGGCGTCATCTACCACAAGACATGC
TTCAAGTGCAGCCACTGCAAGGGGATCCTCTCGGTACGTGTGCATCATGCAAATATA
ATGTCCTGTCGTCCATGGCATAGAAAAACGATCGAGTTTATTTGCATCCCATCTCTG
CGTGAATGCAAGCCACAAAAGCAAGAGTTTTTCTGACGCTGCAAACCGAGTGATGT
ATAATGCGTTGGTCGGCTGCAGATGTGCAGCTACTCTTCCATGGACGGTGTGCTGTA
CTGCAAGACCCACTTCGAGCAGCTCTTCAAGGAGACCGGGAGCTTCTCCAAGAAGT
TCACGCCAGGTAATGCAGCACTACGACGTGATCTTCGGTTGTCACAACAATTCTGTT

TAATTTGTCGTGCCGGAGAGAACATTATGCTGAAAACTTTGTTGTGTAATTCAGGTT
GCAAGTCAGACAAGGGTGAACTGGTACAGCTACCCTCACAAGACTAGTATCATCAT
GATTGATTTTGGAATGTCTCTGTTTATTGTAGTAACTGGATAAATGTTTGCAATGCAA
ATTGGAATCCCTTTGCACTTTGCAGGCAAGGGCCCCAAGCAAGCTATCGTCTGCATT
TTCTGGTACTCAGGATAAGTGTGCAGCATGCCAGAAAACAGTGTACCCGCTGGAGA
AGGTACTGAAATGATGAATATTTTTTTCTTAGTACTTACTTCCCAATATCTAATAATA
ACAAATTGCCATACTTTTGGGAAACAGTAACAACAGTCAATAATTTCTCTTGCCCTA
CTAAATTCTAATGAATGTGTAACGCTAACAAAATTGTTCTATTGTTCAGTTAACTTTG
GAAGGCGAGGCCTACCACAAGAGCTGCTTCAAGTGCTCACACGGGGGCTGCATCCT
GACCACCTCCTCCTACGCCGCGCTCAACGGCGTCTTGTACTGCAAGATCCACTTCGG
GCAGCTGTTCATGGAGAAGGGGAGCTACAACCACATGAAGAAGAAGAGCACGTCCC
AGGAGGTGCTGCCGGACTTGGCTGCCGAGGAGCAACCTCCGCAACCAGCGGCACCG
GAAGATGAGAAAGGAGAGGACAACTAGGAGCAAGCGATGATCCATATATATCACG
CAGACAGAAGTTTGCGTCTGCCATTTTTCTTTACGACGCTTCTTTATTCCCTCCTCTTT
TCTTTTCTTAATCTTTCTGTGAACTCGGTTTGTCATCAAGCTGTAAGTGTACCAGATG
CCCTCGTTACGTCTGAGGCTCATGACAATGGCAATTTTGGCATGGAGCTGTGTTAGC
CTCGCGGAAAAATTGTAACTTTGTAAGCAGAACTCTACTACTTTGAATATACGATCC
TAACAATGTTG

>SbLIM4

CATTTCCTTTCCTCCTCCCCATTCCCATTCATGCAGGACCGACCCCCCACCTCGTATG
CTCTTTCTATCTCCTCCCTCCCTCCCTTCCTGACACCCCCAACATTCCCTGCCCCCCCT
CCTCCCTCCCTCCCTAGCTGTGTCCTGCCTAGCGGTATCAATACCTGGCTGCAGCTCA
CCACCCACCAACCACACCACACCAGAGTTCCCAGCTCCCATATAACCACTGATCAGC
AGCAACTCAAGGCTGGCTGGGGAAGTTCAGAGTGCGGCTACACCATAGTATACCTC
TGTAGGCAGATCAGTGTGTCGTTGCAGAGGAGGGAAGAGGACCAGCCATGGCGACC
TCCTTCCAGGGGACGACCACCAAGTGCACCGCCTGCGACAAGACGGTGTACCTCGT
CGACAAGCTCACCGCCGACAACCGCATCTACCACAAGGCCTGCTTCCGCTGCCACCA
CTGCAAGGGCACCCTCAAGGTAGCCACAAACCTGCATGCTTCAGTTGATCGATCGAT
CTGTATGTAAAAAGCTGAGTCGAGTGAGATGATAGTAGTGATTAGGCGGTGAAAGT
TTACCGGCATTGATTTGTTTTGTCTGAGTGAGGATGTTCTCTGCCGCAGCTTGCCAAC
TACAACTCCTTCGAGGGAGTGCTCTACTGCAGGCCTCACTTCGACCAGCTGTTCAAG
AGGACCGGGAGTTTGGACAAGAGCTTCGAAGGTATCATCAGTTTTTTTCCCCCTCC
CCTTTCGCAATACAAAATAGATCCAGTGGTTCTTGCATGTCCTCACCTACAAATGCTT
CAGGAACTCCAAAGGTTGTCAAGCCAGAAAGAAACGTTGGGAATGAGGTATGCAAC
AACTTAGGATTCATGTTATGCAGAAGAGAATTTTTCTTTTTTTTTCTCTCAGTACCTA
CAAAGCTGCATTTTCACTTCAGTGCCGAGTAGCACAGGAAAAGAAATGCATTCAATC
ATTGATGCACAAACAACTCCTAAAGCATGATGTGAGATGCATGGATTTTGAATATCC
CCATCTGTTAGAGAGGAAGAGACTGAGATAAATAGCCACTCAAAACTTAAGTGTGA
TGCTTGGATCAGAACAGAGTAAATCTAGTCCAATAAAAAGATCAGATATTTATCAG
ATCACAAGAAAATTATTGATAAAACCTTTGATCTTCATGAGGCAGATGACTAGATCA
GTGTTGTATTGTTTTAATGCTTCATATCATCCAGTTGGCACTATTCATCTTTTATTCTT
TATTTATCAACAACTGGCACTAGATGATGCTCATCATGATAGCATAATTGGCAGAAT

GCTGTTAAAGTCTCAAGCGCCTTTGCTGGCACCAGAGAGAAATGTGTTGGATGCAGC
AAGACAGTCTATCCAATTGAGAGGGTAAGATTCTATGTTCAACTATCTCAATCATTC
GTCAGTAGTTTGAGTCTTCTTCACTTGACAACCCGTCACTGAATTCTCATTCAAGAAT
CAGTGAATCACATCATATAAGATTCCCAGATAAAAAACGTTATATCCATGAAGACA
GCGATTGCTGCATTCTCAAGTTGAAAGTTCAATTATTGATATTTTTGCCTAGAGATAA
GAAAAGGTGACAGGTATATTGTACCTTTTGAGAGTTAATTTGCGTGATTATCAGTAT
GCTGATTGCAACTCACCGATTAAGCATCCAGGTTACTGTCAACAACACTATGTATCA
CAAGAGCTGCTTCAAGTGCTGCCATGGAGGATGCACCATCAGCCCTTCTAACTACAT
TGCGCACGAGGGGAAGCTCTACTGCAAGCACCACCACATTCAGCTGATCAAAGAGA
AGGGAAACTTCAGCCAGCTTGAGAATGATCACGAGAAGACGTCACAGGCTGGGTCA
CTGGAGGATGAAGAAGAGTATTAATCACTGAGCACTATCACAGATGAATAATTAAT
CTTCTTGCATTTGCTTAGAGCACTATATATTTCTGTGGCATGGTTCGATTTCAATTTT
ACCAATGGAGCTCATGTGCGTTCAGAGAACAATGAAATTATCGTCTTGTATCTGCAT
GTAAACCTTTGTTATCTTTGAGTCAAATACTCTACCTGTTTCACCATTTTCATTGATC
AGAAGATGCCTCTGCAGTCTGCACCAAGAAGACACAGAACTGCAAGCTCTTGGT

>SbLIM5

CCAACTCGCCGTCCGCGCCTCCGTTATTCTCCCACGCCTCTCCCTCTCCCTCCACGAC
ACACAGGCCGCCTGAGCTGTGCTGCCCTCTCCTCTCCTTCCGTTTTCACCTCCGCCAA
TCATCCACCCCACCCGTCGCCGTCGCCTCCTTCTCTTTCCCTCTCTCCTTTTTCTCCCT
CCTCGATCCTTCGCTACGACGGAGCAGCAGGAGGGCGGCGACGGCGAGGGGATGTC
GGGGGCGTGGGGCGGCACCACGCAGAAGTGCGCGTCGTGCGGCCGGACGGTGTACC
CCGTCGAGGAGCTTGCCGCCGACGGCCGCGTCTACCACCGCCCCTGCTTCCGGTGCC
ACCACTGCAAGAGCACACTCCAGGTCCCTCTCTCTTCAGCCCCCTTCGTTTCCTCCCT
CCCCTGCTCGGCTCGCGCATGGACTCTTGGGGTGTTGTCGAGCACAGTCTTGTTATTA
CGAAGCGAAAGTTCACGTGTAGGATCAGTCGCACTGTTACTGGTGCTTTTGTGATTC
TGGCTATTATTGCGAGCGTCGCTCAAGTCAGAGTAGCTGGGCTGTCTTGTCTTGCTTT
CTTGTCCTTGCTTCGAATTGCTTGGCTGCTTCCTTCAATTGCTGTACTTCCTTGGTCTG
GGTTTGTCAGTTATAGGTTGCATGATCATGTCGACTGCGATTGTGCAGGATTTGCGC
ACGGAGTTACTTGTTACGCGTTGAATTTGTACTGGTTCTTGCTTGTTAGCGAACTGGG
TACAAAATCACGGATAGGGAGTACACAGTACAGGGTACAGAATTTGTTGTTTCATTT
AAGCACGATTGTGCCATCAGTGAACTTTTCTCTTGCAACCACATGGCAAGTGCGCAT
TTAAATCTTCAGCTGTTCCCCGATGAATACCTAATGACAATTGATCTTACCTTTTAAT
CATGATAATGTAGAGTGTGAAATAATGTCATTATCTAAAGATTTCTGTCCTTCTATG
AATATAATATGCATTTAACTTTTTGAGGAATCCATCTCAATAGGCTACTGATGTAAT
GAGATGTCATATCTATCACTTTAAGTATTAAAACAGTGCCTTGCTATATCATGATCA
CTTTGTACTTGCTTTCCCTTGCACATGGCATAGCTGTCATAATTCTTGCAGTCAGTTG
ACATATAATCATGTAATAATGCAGTGCAAGACAAATGCCTTTCCAATATGGATGTAG
TATTGGTACCTGATTAGACTTGCTTGCATTATTCCATGAGAACGTGTTGTTTGGCACA
GCTGCACCTTAATTCAGAGACTTCTAATTTTGTTGAGAGCAATAATGATCATAGTGA
ATCTCTTACGCTACAATCATACTAGTGGGCATCAGTGCTATTTTTTCTTATTTGCAGC
CAAAGCTACACTTGTGAAACCTAACCAGAATAATACTAGTTCCTGCATGATAGTGAG
CTGGGTAGAAATCACAAAGCGCAAGACTTGAGACATGCTTGAAGATTTTTTCTCTT

CTAGAAAAACAGTCTAGATTCTTGAAGCACTTTTGTTCTTGGTAAAGTGGGGAGTTC
AGTGTACTGGAATTTGTTTGTATATAATTGTTTTCCCTCAAACATGCAGGATAGCTGT
GTATCAATATATTAAGAAGAAAAAAAAGAGGGGGAGTAGAGCCCCATACAGATCAC
CCGCACACATAAACAGCAGAACAAAACTCAGAAGAAGCCTATGCTACCACTATTAC
TTTAGAGCACATTGTTTGACACACTGTAGCTTTATTCAGAAGCTTTACATTTTTACAA
CAGCAATAATGATAATACTTATGCTACGTCAAACTAGTTGACAATGGTATTATCTTC
ATATGTTTACAGCCAAAATGTGCATCTGTGAAACCTAATCAAACTAATACTGGAGCT
AACCCCATTCTTCTGGTTCTTCTTTCTGCAGTTTAGTAATTATTCTTCCGTGGAAGGT
GTCCTATACTGCAAGCCTCACTATGACCAGATATTAAAATCAACAGGCAGTTTGGAG
AAAAGTTTTGAAGGTATTAGAGACAATCAATTGGACTAAGATGCTGTGATATATATA
TATTTTAAATACCTTATTGTCCTCACAATTTGCATGAGTAGATGCTACAGTGCATATG
TTTCTGATCGAAATTATACAACTCCAAAAAATAAATCAACCCTAGGAACAGCCTCCC
CAAAGCATTATGATTAAGAAGAAGCCTCAACCAGACAAGCCAAGAAAAAACCCCA
AACCCTGGATTCACCCTATAGGGCCATAACATAATCTGGGCCGACCACGACTTTATG
GGGTTGTGACAACTGCAACTACCCTGATAGGAGGGCCCAAACCAACCACAAGCACC
ACAGGCGCCCTGGCACACTGTCTTTTTGGGTTGCACACAAAGGGATTTTTTTTGGTTG
CCACCAGGATTTGGACCATGGCCGGTTTCTCGTTCAATTGGGGAGCTTACTGCTACA
CTACAAGAGTGTTGGCTACAGTTCTTATAGCTGCAATTGAATATGAAATTATATTAT
CACATCTTATTATGCACACGATCACAATGGTTTTCTCTTTTCATCCTGTAAACCAGGT
GCTTTATTTTAGCATTGTTTTTATAATATAACACATCTTTGTGAGCTAACACTTTTTCA
GTGGTGTTTGTGTGGGATACGACTCTCTTCTTCTACTAATGCAATGATACATATCTCT
CCTGCGTGTTTGAGAAAAAAGCGAACTAATGTTGTTCTCTGAAATATTACTGTGTTA
TATGTCAGGTGTGGCCCGATCAGCTAAGTCAGAAAAATCAAATGGACATAAGGTAT
CTGAGCTTCTCTAGGGCATGCCTGGTGCCTAATACATTTCACTGATACTGCAATATA
CAGCAAATTTATGCCAATTTCCTCTATTTTTTTTATTGTCAATGTGCAGGGCCAACAA
AGCAGTAGATTCTCTAATATGTTTGTTGGCACACAAGAGAAATGTGTAGTTTGCAAC
AAGACTGTGTACCCGCTTGAGAAGGTCAGTATTAGTCAGTCTTCCTCTTTTGTACTAA
ACTAGAGTAAAAAATGCTTTGGCAGTTAAGCACAGTTTGCTCCTAAGTGTACTCGCT
GACAACTATTCATGTCCTAAGTGTGTGTGTGTGTGTGTATGTGTGTGGTGGTGGTAG
GTGTGTGTGGGGGGGTGGGGTGGGGGGAGTCCTCTGTTTAAATTCATAATCTTGCTC
AGAGGTATTTTATCAGAAGCATAGTTCAGCTGTGTACCGTCCACGGATGGTAGAGGC
TGGAACTTTGTTCCATTATCTGAAAAATCAGAAAGCATAGTTTCTTTACAACTGCTA
ATGTAATTAAACTTTTTATGACTATTAGTATTGGAAAAATTCTATAGTAATTGCAATT
TCAGGATAGTGCATCTAATATAATTCTCCATTGCATGTAACATGTAGAAAAAAAATC
TTAGTATTAAAAATTCGATCTCTTCACCACAAGCCAAAGTGATGGTGTATGTCCTTC
AGAATTCAGTGACCTCTATCATAGTACACTAAGTGCTTTTTTTTGGGAATGGAATCA
TAGTGCACTAAGTGCATAGCTGTCAAAGGGTCATAGAATAATTTTAACCAGCCTCTT
TTGCAGAGTATAATTTAAGGGCACATTTACATCACGCTGTTACATTTTTGCAGGTTGC
TCTTAATGGAAATTCTTATCATAAATCATGCTTCCGCTGCACCCATGGTGGTTGTACG
CTCAGCCCATCCAATCATATCACCCATGAAGGCAAACTTTATTGCAAGACCCACCAT
TCTCAACTGTTTATGGTTAAGGGGAATTTCAGTCAGTTCGAGGACAATTCTGGGAAT
GCAAAAGTTGCTAGTGAGAAACAACCAGAAACTGAAGAAGCCACCAAAAATCCAA
ATCAAGGTGATGAAGTCACACAGAAACCAGTAGAAAATGAACCTATAGATGAGAAA

ACTTCAAAGAATGATGTTGCAGCTGAGAAACAATTGCAAAGTAGTGTTGATGTCAC
AAAACCATCTGAAAGCACCATGGCAGAAAATGAACGAGGTACTGAGAGTGAGTCAA
AGAGTAATGTTGTCAACAACAAGCCATCAGAAAGTAGTGTAGAAAAGCCACTGCAG
AACAGTGTGGTTGATGTAAAGCCATCAGGAAACAGTGCAGCCATGAGAAAACCCTG
GCAACGAAGTCTGCAAACAGATAAACCATTTCTGAGTAGCACAAGCACTGTAAAGC
CATCACCGAGCAGTGATGCCACTGAGAAGCCATCATCAAGTAATGGGGTTGATATG
AGACAGCCTGAAAGCAGCACATTAGTAAAAAAACCAGGGCAGCAAAATGTGCCAA
CTGAGAATCCACCACAGATCGTTTTACCATCAGATAAGCCATCAGCGACCAGTGTAG
ATGATGCAAAGCCATCAGAAAGCAGCAAAGTGGTCAAAAAACCATGGCAACGCAAT
ATGGCTGCTGAGAAGCAATTACAGAACAGTGCACCAACTGAGAAGTCACATAAAAG
TGTAGCTACTGATAAACCATCACCAACAACCGACATGAAGTCATTAGATAACACCA
CAGAAGTTAAAAGTCCATGGGGACGCAGGATGTTCAATAATAAGTCACTAAAGAGC
ACTGTAGGTACTGAGAAATCGTCTGCAACCAGTGTGGTTGATGTGAGACCAGGGGA
AACCAGTACAGTAGCCCCTGTGCCACAGCAACAAACTGAAAACGTTGAGAAACCTT
CAGACACCAGTGCAGATGATGCAAAGAGTGCAGATGATGTGAAGAGTGCAGATGAT
GTGAAGAGTGCAGATGATGTGAAGCTATTGGTCGCCAGTCCAGATGACACTAAGAG
CGCAGATGATACGAAGACTACAGATGGTGTAAAGCCATCAGAGACTACTGCAGCTG
TAGTTAGAAAGTCATGGCAACGCAACATAGACACTGGGAAGCAACCGCTGACCACT
GCAGTTGATCCAAAGACGACTGAAGCTAGTGGAACTGTCAAAAGGTTGTGGCAGCG
CAGTGCTGCAACTGAGAAGCTGTCACAAAGTGGTACGGCTGTTGTGAAACCATTGC
AAAGCAGTGTGGCTGTCTCCAAGCCGTTCCAAAGCAACGTAGCTGTGAAAAAGACA
TGGCAAAGAAGTGTAACTCCAGAAAACCAGCGAGAGAGTAATATGTCTAGCAATAA
GCCATTGGCAAGCAAGGTGGTCGTTGAGAGTCTAGTGCAAAGCAACACGGTTGAGA
AAATGTTTCAGAGCAATGTACCTACTGAGGAGCCACAGAAAGTCATTGTGGCCACT
GAAAACCAATCGCAGACCATCAAAGTTACAAAGAAGAGCAATGATACATCCATGAA
GCTATCTGTAACAAGTGAGACAACCAAAGTGCCACCACTTGCTGCAACCGCATTGC
AAAGTGATGTTTCCACAGAGAAACCATCACAAACTGACATGCCTACCATAACACCT
AGTCAGATCCCTGAGCCCACTGAGAAACCATCAGAAAGTGCTTTTAATGCTGAGAA
GTTATCAAATGTTGACACTGCTACTGAGAAACCACTTCAAAGTATGATCACTGAGAA
GGTAGAAAGTGTAGCAGCCACATTGAAGCCATCTCAAAGTGATACAGCCCCTCAGG
AGATCTTGGAGAGGAATATGGATACTGAGAAAATATTGCAAAGTGCCATGGCTGTT
GAGAAGCCACCTCCAACCAATTTAATCACTGAGAAGCCATCAATAAAAGATGCTTC
AGAGGAGCCAGTTCAAACTAACGAACAATCTGAGCAGCCACTGAAAACTGAAGAGG
TTGAGAAGCCACATCAAAGTGAAAAGATTGCTGCGGAGACGAAAGGGAGTGAAGT
ATCTATTGAGAATATGCTAGAGCTTGAAAGTAATGCCACTAAATTAAACAAGGATC
ACTCAGAACCTGAAGGGCTTTCATCTGGTACGAATCCTCCAGACTTCCAAAGCAATC
CAAATGCTGGGCAGCAATTAGAGTCTAAAGGCATTGTGGCTGAGAAGGAAGCTGAC
AATATAATGGAAGCTAAAAATGATGCAGTTGCTGAGCACTCATCAGAATCTCAACA
CGTTGCACCTGCTGAGGTTCCAAAGGAGCAACCATCAGAACATCAGAAGGATGCGG
ATATGCAGCTGCTATTGGAACCTCAAAATGAGGATCATTCTGGGAATCCACTAGAGC
CTGTTAGTGATACAGCTGCCAAAGATTCATCAGAGCCTAAAAGTGACATAGCTACA
GAAAAACTGCAGAATCACAAAATAATGCAGATCAGTCAGTTGAGCAGTCACCAGA
ACCGCAAAGCGATAAATCAACTGAGAAGCCAGAAGTGCATCAAAGTAGCACACCTA

GTGATGAGCTTTCTAGGCTTCAAAGTGATGCAGGTGCTGATAAACTATCAGTACCAT
CATCAGATCCTGAGAGCAATGCATCTGTCAGTAAGCCATCAGAGTCTCAAACTGATG
TGATTACCATGGAGGCACCAGAACTCCAAATTGATGCTCTTCCTGATAAGGCAACTG
ATCAGCCAGTGAAACCTCAAGATGATGCATCTGCTAAGAAGCCAATGGGAACTGAA
AGTGATGCTGCTTGTGATAAACCGTCGGAAAGCAGCTCAGATACTGAAACACTTCCT
GTATGCCATCAGAACAGCAGCATAACCACTGATGAACCTGTACAGGGTGACATTTCT
TATGAGACACCACATCAGAGAAGTGCACCCATAGAAACAACACCAGGAAGTGACAC
AGTTGTTGAAGATTGCATACACCATGAAGATACCAGCAGCAAACCATCAGAGGAAA
ACAAAGCTATTGAGGAGCCAGAAGAGGTGAGTGCCAAGCTGCCAGATGACCATGTG
ACTTCTGAGAAGTCATCAGAGGAAGACAAGGAAAATGCAGAGCCATCAGTAGACAA
TGCTCCCCTGGGGAAACCATTGGAGGCCAATGAGGAGAGCTCAAAGTCTTCAGGGG
ATACTGTAACTCCTGAGAAGCCACTGGAGGAGGACGAGACGAGTGCGGAGCCATCA
GAAAGTGACGCATCCTTTGGGAAACTGTTGGAGGCCGATGAAGTGAGTGCCAACCC
ATCAGAGGATATTGCAACTCCTGAGAAGCCACTGGAGGAAGGTGTGGCAAGTGTGG
AACCATCAGAAGACAATTCTGTTCTCGATAAACCATTGAAGGAAGAAGAGGTCACT
GCCAAGCCATCGAAGGATGTTGTAACTCCCGAGAAGCCACTGGAGGAAGGCTCAAC
AACTGCAGAGCGATTAGAAGACAATGCTGCCATTGGGGAAGCAAAAGAAGAAGAT
GAGGTGATTCCCAAGCCAGAGTCCAGTGTGGCACTTGAGAAGTCATTGGAGGGAAG
TGAGGCAAGTGTAGAGCCATTGGAAGACAATGCTGCTCTTGAGAAACCATCGGAGG
ACGACGAGGCAAATGCCAAGTCATCAGAGGACAGTGTAGCTGTGGAGAAGCCACAG
CAGGAAGAGGACAATGTAGCTACGGAGAAGCCACAGCAGGAAGAGGACAATGGTG
TCAAGGCATTAGAGGAGGACGTGTCCCCTGAGAAATCAGCCAATGGGAAACCATTA
GAGGAAGAGGACCCAGTCCATGAGAAGCTGGCAGACGCCGACACAGTCGTTGAGCC
GTCATCTCAGGACGACACTGCCACTGAAAAGCCCTCAGCTACAACTGACACTGCAG
AAACTGCATGAGACAGCAGTTAGCTGGTTACAGTTACTAGTTAGCAACAGTTTTTAA
CAGAGTGTGGATCGAATACTATTTATTGGTGATAGTTCGGCGTGCCTTCTGTTTGTTT
TTTTTTTGGGGTTTTGTACATTGCTTGAGTTGTAAAATTTGAGGGAAAGAAACTATA
AGTTTTGATTTGATTTGATTTTTTACAAGCAATAATCCCCTCCTCTCCATGTTAGGAG
GAGGATACCATTGAAGTTCAATATATAGGATTTGTGTGCGGTTTTGATTTTTGTTTGG
ATGGTGTACTTTGTAAATGATTTTGTGCAAATATTACTGTGCTTGCTCATTATTTGGT
TTTCTGCGTGTAAATTGATCTAGCAAGATTTTATGTGCAAATGGTGTGGCTAGGCTTT
CTATAATCA
