# Supplementary material for: A genome‑wide approach to the systematic and comprehensive analysis of LIM gene family in sorghum (Sorghum bicolor L.)
Source: Genomics Inform. 2023 Sep 27;21(3):e36. doi: 10.5808/gi.23007 (PMC10584642; doi:10.5808/gi.23007)
Supplement: Supplementary Fig. 6. — Full-length coding sequences of LIM gene families of Sorghum bicolor (Doc). [file gi-23007-Supplementary-Fig-6.pdf]

Supplementary Fig. 6. Full-length coding sequences of LIM gene families of *Sorghum bicolor* (Doc).

>SbLIM1

ATGTTTAGCGGGACGCAGCAGAAGTGCAAGGTGTGCACCAAGACGGTGTACCCGAT
GGACCAGCTCTCCACCGACGGCGTCGCCTTCCACCGCTCCTGCTTCAAGTGCCAGCA
CTGCAAGTCCACCCTCTCCCTGAGCAACTATTCCTCGTTCGAAGGAGTGCCGTACTG
CAAGGCCCATTTCGAGCAGCTGTTCAAGGAGACCGGGAGTTACAACAAGAGCTTCC
AATCACAATCACCCGCAAAGATTACTCCGGAAAAGTTGGCCCCTGAGCTGACCAGA
TCACCAAGCAAAGCTGCAAGGATGTTTTCAGGAACACAAGACAAGTGTGCGACTTG
CGGTAAAACCGCATATCCTCTTGAGAAGGTAACAGTTGAAGAAAAGGCATACCATA
AGTCATGCTTCAAATGCTCCCACGGGGGCTGTGCGATTACACCTTCCAACTATGCAG
CCTTGGAGGGCATCCTCTACTGCAAACACCATTTCTCTCAACTTTTCAAGGAGAAGG
GAAGCTACAACCACTTGATCAAGTGTGCTTCGGTCAAGCGCGCTGCTGAAGCACAG
CCAGAACAACCAGCCTCCGATTCCTCCTGA

>SbLIM2

ATGTCTTTCACCGGCACGCAGGACAAGTGCAAAACCTGCGACAAGACGGTCCACTT
CATCGACCTCCTCACCGCCGACGGCGTCTCGTACCACAAGACATGCTTCAAGTGCAG
CCACTGCAAGGGCACACTCTCGATTAGCAGCTACTCTTCCATGGACGGTGTCCTGTA
CTGCAAGACGCACTTTGAACAGCTCTTCAAGGAGACAGGGACCTTCTCCAAGAAATT
TCAAGGTGGAGCATCTTCAACCAAGACCGACCAGGCAAAGGCTCCGAGCAAGCTAT
CATCTGCATTCTCTGGAACTCAAGATAAATGCGCAGCCTGCCAGAAAACCGTGTATC
CATTGGAGAAGATGACGTTGGAAGGCGAGTCTTACCACAAGAGCTGCTTCAAGTGC
TCGCACGGGGGCTGCATCCTGACAACCTCCTCCTACGCCGCGCTCAATGGGATCCTC
TACTGCAAGATCCACTTCTCGCAGCTGTTCAAGGAGAAGGGCAGCTACAACCACCTC
ATCCAGACGGCGCAGACCAAGAAGAACGAGGCTGCGGAGGCCGCACCGGAGGCAC
CGGCGGATGCAGGCGCGGCTGAGCCAGAAGCAGCGTAG

>SbLIM3

ATGTCTTTCACCGGCACGCAGGACAAGTGCACGGCGTGCGACAAGACCGTCCATTTC
ATCGACCTCCTCACGGCCGACGGCGTCATCTACCACAAGACATGCTTCAAGTGCAGC
CACTGCAAGGGGATCCTCTCGATGTGCAGCTACTCTTCCATGGACGGTGTGCTGTAC
TGCAAGACCCACTTCGAGCAGCTCTTCAAGGAGACCGGGAGCTTCTCCAAGAAGTT
CACGCCAGGTTGCAAGTCAGACAAGGGTGAACTGGCAAGGGCCCCAAGCAAGCTAT
CGTCTGCATTTTCTGGTACTCAGGATAAGTGTGCAGCATGCCAGAAAACAGTGTACC
CGCTGGAGAAGTTAACTTTGGAAGGCGAGGCCTACCACAAGAGCTGCTTCAAGTGC
TCACACGGGGGCTGCATCCTGACCACCTCCTCCTACGCCGCGCTCAACGGCGTCTTG
TACTGCAAGATCCACTTCGGGCAGCTGTTCATGGAGAAGGGGAGCTACAACCACAT
GAAGAAGAAGAGCACGTCCCAGGAGGTGCTGCCGGACTTGGCTGCCGAGGAGCAA
CCTCCGCAACCAGCGGCACCGGAAGATGAGAAAGGAGAGGACAACTAG

>SbLIM4

ATGGCGACCTCCTTCCAGGGGACGACCACCAAGTGCACCGCCTGCGACAAGACGGT
GTACCTCGTCGACAAGCTCACCGCCGACAACCGCATCTACCACAAGGCCTGCTTCCG
CTGCCACCACTGCAAGGGCACCCTCAAGCTTGCCAACTACAACTCCTTCGAGGGAGT
GCTCTACTGCAGGCCTCACTTCGACCAGCTGTTCAAGAGGACCGGGAGTTTGGACAA
GAGCTTCGAAGGAACTCCAAAGGTTGTCAAGCCAGAAAGAAACGTTGGGAATGAGA
ATGCTGTTAAAGTCTCAAGCGCCTTTGCTGGCACCAGAGAGAAATGTGTTGGATGCA
GCAAGACAGTCTATCCAATTGAGAGGGTTACTGTCAACAACACTATGTATCACAAG
AGCTGCTTCAAGTGCTGCCATGGAGGATGCACCATCAGCCCTTCTAACTACATTGCG
CACGAGGGGAAGCTCTACTGCAAGCACCACCACATTCAGCTGATCAAAGAGAAGGG
AAACTTCAGCCAGCTTGAGAATGATCACGAGAAGACGTCACAGGCTGGGTCACTGG
AGGATGAAGAAGAGTATTAA

>SbLIM5

ATGTCGGGGGCGTGGGGCGGCACCACGCAGAAGTGCGCGTCGTGCGGCCGGACGGT
GTACCCCGTCGAGGAGCTTGCCGCCGACGGCCGCGTCTACCACCGCCCCTGCTTCCG
GTGCCACCACTGCAAGAGCACACTCCAGTTTAGTAATTATTCTTCCGTGGAAGGTGT
CCTATACTGCAAGCCTCACTATGACCAGATATTAAAATCAACAGGCAGTTTGGAGAA
AAGTTTTGAAGGTGTGGCCCGATCAGCTAAGTCAGAAAAATCAAATGGACATAAGG
GCCAACAAAGCAGTAGATTCTCTAATATGTTTGTTGGCACACAAGAGAAATGTGTAG
TTTGCAACAAGACTGTGTACCCGCTTGAGAAGGTTGCTCTTAATGGAAATTCTTATC
ATAAATCATGCTTCCGCTGCACCCATGGTGGTTGTACGCTCAGCCCATCCAATCATA
TCACCCATGAAGGCAAACTTTATTGCAAGACCCACCATTCTCAACTGTTTATGGTTA
AGGGGAATTTCAGTCAGTTCGAGGACAATTCTGGGAATGCAAAAGTTGCTAGTGAG
AAACAACCAGAAACTGAAGAAGCCACCAAAAATCCAAATCAAGGTGATGAAGTCA
CACAGAAACCAGTAGAAAATGAACCTATAGATGAGAAAACTTCAAAGAATGATGTT
GCAGCTGAGAAACAATTGCAAAGTAGTGTTGATGTCACAAAACCATCTGAAAGCAC
CATGGCAGAAAATGAACGAGGTACTGAGAGTGAGTCAAAGAGTAATGTTGTCAACA
ACAAGCCATCAGAAAGTAGTGTAGAAAAGCCACTGCAGAACAGTGTGGTTGATGTA
AAGCCATCAGGAAACAGTGCAGCCATGAGAAAACCCTGGCAACGAAGTCTGCAAAC
AGATAAACCATTTCTGAGTAGCACAAGCACTGTAAAGCCATCACCGAGCAGTGATG
CCACTGAGAAGCCATCATCAAGTAATGGGGTTGATATGAGACAGCCTGAAAGCAGC
ACATTAGTAAAAAAACCAGGGCAGCAAAATGTGCCAACTGAGAATCCACCACAGAT
CGTTTTACCATCAGATAAGCCATCAGCGACCAGTGTAGATGATGCAAAGCCATCAG
AAAGCAGCAAAGTGGTCAAAAAACCATGGCAACGCAATATGGCTGCTGAGAAGCA
ATTACAGAACAGTGCACCAACTGAGAAGTCACATAAAAGTGTAGCTACTGATAAAC
CATCACCAACAACCGACATGAAGTCATTAGATAACACCACAGAAGTTAAAAGTCCA
TGGGGACGCAGGATGTTCAATAATAAGTCACTAAAGAGCACTGTAGGTACTGAGAA
ATCGTCTGCAACCAGTGTGGTTGATGTGAGACCAGGGGAAACCAGTACAGTAGCCC
CTGTGCCACAGCAACAAACTGAAAACGTTGAGAAACCTTCAGACACCAGTGCAGAT
GATGCAAAGAGTGCAGATGATGTGAAGAGTGCAGATGATGTGAAGAGTGCAGATGA
TGTGAAGCTATTGGTCGCCAGTCCAGATGACACTAAGAGCGCAGATGATACGAAGA

CTACAGATGGTGTAAAGCCATCAGAGACTACTGCAGCTGTAGTTAGAAAGTCATGG
CAACGCAACATAGACACTGGGAAGCAACCGCTGACCACTGCAGTTGATCCAAAGAC
GACTGAAGCTAGTGGAACTGTCAAAAGGTTGTGGCAGCGCAGTGCTGCAACTGAGA
AGCTGTCACAAAGTGGTACGGCTGTTGTGAAACCATTGCAAAGCAGTGTGGCTGTCT
CCAAGCCGTTCCAAAGCAACGTAGCTGTGAAAAAGACATGGCAAAGAAGTGTAACT
CCAGAAAACCAGCGAGAGAGTAATATGTCTAGCAATAAGCCATTGGCAAGCAAGGT
GGTCGTTGAGAGTCTAGTGCAAAGCAACACGGTTGAGAAAATGTTTCAGAGCAATG
TACCTACTGAGGAGCCACAGAAAGTCATTGTGGCCACTGAAAACCAATCGCAGACC
ATCAAAGTTACAAAGAAGAGCAATGATACATCCATGAAGCTATCTGTAACAAGTGA
GACAACCAAAGTGCCACCACTTGCTGCAACCGCATTGCAAAGTGATGTTTCCACAG
AGAAACCATCACAAACTGACATGCCTACCATAACACCTAGTCAGATCCCTGAGCCC
ACTGAGAAACCATCAGAAAGTGCTTTTAATGCTGAGAAGTTATCAAATGTTGACACT
GCTACTGAGAAACCACTTCAAAGTATGATCACTGAGAAGGTAGAAAGTGTAGCAGC
CACATTGAAGCCATCTCAAAGTGATACAGCCCCTCAGGAGATCTTGGAGAGGAATA
TGGATACTGAGAAAATATTGCAAAGTGCCATGGCTGTTGAGAAGCCACCTCCAACC
AATTTAATCACTGAGAAGCCATCAATAAAAGATGCTTCAGAGGAGCCAGTTCAAAC
TAACGAACAATCTGAGCAGCCACTGAAAACTGAAGAGGTTGAGAAGCCACATCAAA
GTGAAAAGATTGCTGCGGAGACGAAAGGGAGTGAAGTATCTATTGAGAATATGCTA
GAGCTTGAAAGTAATGCCACTAAATTAAACAAGGATCACTCAGAACCTGAAGGGCT
TTCATCTGGTACGAATCCTCCAGACTTCCAAAGCAATCCAAATGCTGGGCAGCAATT
AGAGTCTAAAGGCATTGTGGCTGAGAAGGAAGCTGACAATATAATGGAAGCTAAAA
ATGATGCAGTTGCTGAGCACTCATCAGAATCTCAACACGTTGCACCTGCTGAGGTTC
CAAAGGAGCAACCATCAGAACATCAGAAGGATGCGGATATGCAGCTGCTATTGGAA
CCTCAAAATGAGGATCATTCTGGGAATCCACTAGAGCCTGTTAGTGATACAGCTGCC
AAAGATTCATCAGAGCCTAAAAGTGACATAGCTACAGAAAAAACTGCAGAATCACA
AAATAATGCAGATCAGTCAGTTGAGCAGTCACCAGAACCGCAAAGCGATAAATCAA
CTGAGAAGCCAGAAGTGCATCAAAGTAGCACACCTAGTGATGAGCTTTCTAGGCTTC
AAAGTGATGCAGGTGCTGATAAACTATCAGTACCATCATCAGATCCTGAGAGCAAT
GCATCTGTCAGTAAGCCATCAGAGTCTCAAACTGATGTGATTACCATGGAGGCACCA
GAACTCCAAATTGATGCTCTTCCTGATAAGGCAACTGATCAGCCAGTGAAACCTCAA
GATGATGCATCTGCTAAGAAGCCAATGGGAACTGAAAGTGATGCTGCTTGTGATAA
ACCGTCGGAAAGCAGCTCAGATACTGAAACACTTCCTGTATGCCATCAGAACAGCA
GCATAACCACTGATGAACCTGTACAGGGTGACATTTCTTATGAGACACCACATCAGA
GAAGTGCACCCATAGAAACAACACCAGGAAGTGACACAGTTGTTGAAGATTGCATA
CACCATGAAGATACCAGCAGCAAACCATCAGAGGAAAACAAAGCTATTGAGGAGC
CAGAAGAGGTGAGTGCCAAGCTGCCAGATGACCATGTGACTTCTGAGAAGTCATCA
GAGGAAGACAAGGAAAATGCAGAGCCATCAGTAGACAATGCTCCCCTGGGGAAAC
CATTGGAGGCCAATGAGGAGAGCTCAAAGTCTTCAGGGGATACTGTAACTCCTGAG
AAGCCACTGGAGGAGGACGAGACGAGTGCGGAGCCATCAGAAAGTGACGCATCCTT
TGGGAAACTGTTGGAGGCCGATGAAGTGAGTGCCAACCCATCAGAGGATATTGCAA
CTCCTGAGAAGCCACTGGAGGAAGGTGTGGCAAGTGTGGAACCATCAGAAGACAAT
TCTGTTCTCGATAAACCATTGAAGGAAGAAGAGGTCACTGCCAAGCCATCGAAGGA
TGTTGTAACTCCCGAGAAGCCACTGGAGGAAGGCTCAACAACTGCAGAGCGATTAG

AAGACAATGCTGCCATTGGGGAAGCAAAAGAAGAAGATGAGGTGATTCCCAAGCCA
GAGTCCAGTGTGGCACTTGAGAAGTCATTGGAGGGAAGTGAGGCAAGTGTAGAGCC
ATTGGAAGACAATGCTGCTCTTGAGAAACCATCGGAGGACGACGAGGCAAATGCCA
AGTCATCAGAGGACAGTGTAGCTGTGGAGAAGCCACAGCAGGAAGAGGACAATGT
AGCTACGGAGAAGCCACAGCAGGAAGAGGACAATGGTGTCAAGGCATTAGAGGAG
GACGTGTCCCCTGAGAAATCAGCCAATGGGAAACCATTAGAGGAAGAGGACCCAGT
CCATGAGAAGCTGGCAGACGCCGACACAGTCGTTGAGCCGTCATCTCAGGACGACA
CTGCCACTGAAAAGCCCTCAGCTACAACTGACACTGCAGAAACTGCATGA
