# Supplementary material for: A genome‑wide approach to the systematic and comprehensive analysis of LIM gene family in sorghum (Sorghum bicolor L.)
Source: Genomics Inform. 2023 Sep 27;21(3):e36. doi: 10.5808/gi.23007 (PMC10584642; doi:10.5808/gi.23007)
Supplement: Supplementary Fig. 7. — Full-length peptide sequences of LIM gene families of Sorghum bicolor (Doc). [file gi-23007-Supplementary-Fig-7.pdf]

Supplementary Fig. 7. Full-length peptide sequences of LIM gene families of *Sorghum bicolor* (Doc).

>SbLIM1

MFSGTQQKCKVCTKTVYPMDQLSTDGVAFHRSCFKCQHCKSTLSLSNYSSFEGVPYCK
AHFEQLFKETGSYNKSFQSQSPAKITPEKLAPELTRSPSKAARMFSGTQDKCATCGKTAY
PLEKVTVEEKAYHKSCFKCSHGGCAITPSNYAALEGILYCKHHFSQLFKEKGSYNHLIKC
ASVKRAAEAQPEQPASDSS

>SbLIM2

MSFTGTQDKCKTCDKTVHFIDLLTADGVSYHKTCFKCSHCKGTLSISSYSSMDGVLYCK
THFEQLFKETGTFSKKFQGGASSTKTDQAKAPSKLSSAFSGTQDKCAACQKTVYPLEKM
TLEGESYHKSCFKCSHGGCILTTSSYAALNGILYCKIHFSQLFKEKGSYNHLIQTAQTKK
NEAAEAAPEAPADAGAAEPEAA

>SbLIM3

MSFTGTQDKCTACDKTVHFIDLLTADGVIYHKTCFKCSHCKGILSMCSYSSMDGVLYCK
THFEQLFKETGSFSKKFTPGCKSDKGELARAPSKLSSAFSGTQDKCAACQKTVYPLEKLT
LEGEAYHKSCFKCSHGGCILTTSSYAALNGVLYCKIHFGQLFMEKGSYNHMKKKSTSQE
VLPDLAAEEQPPQPAAPEDEKGEDN

>SbLIM4

MATSFQGTTTKCTACDKTVYLVDKLTADNRIYHKACFRCHHCKGTLKLANYNSFEGVL
YCRPHFDQLFKRTGSLDKSFEGTPKVVKPERNVGNENAVKVSSAFAGTREKCVGCSKT
VYPIERVTVNNTMYHKSCFKCCHGGCTISPSNYIAHEGKLYCKHHHIQLIKEKGNFSQLE
NDHEKTSQAGSLEDEEEY

>SbLIM5

MSGAWGGTTQKCASCGRTVYPVEELAADGRVYHRPCFRCHHCKSTLQFSNYSSVEGV
LYCKPHYDQILKSTGSLEKSFEGVARSAKSEKSNGHKGQQSSRFSNMFVGTQEKCVVCN
KTVYPLEKVALNGNSYHKSCFRCTHGGCTLSPSNHITHEGKLYCKTHHSQLFMVKGNFS
QFEDNSGNAKVASEKQPETEEATKNPNQGDEVTQKPVENEPIDEKTSKNDVAAEKQLQ
SSVDVTKPSESTMAENERGTESESKSNVVNNKPSESSVEKPLQNSVVDVKPSGNSAAMR
KPWQRSLQTDKPFLSSTSTVKPSPSSDATEKPSSSNGVDMRQPESSTLVKKPGQQNVPTE
NPPQIVLPSDKPSATSVDDAKPSESSKVVKKPWQRNMAAEKQLQNSAPTEKSHKSVAT
DKPSPTTDMKSLDNTTEVKSPWGRRMFNNKSLKSTVGTEKSSATSVVDVRPGETSTVAP
VPQQQTENVEKPSDTSADDAKSADDVKSADDVKSADDVKLLVASPDDTKSADDTKTT
DGVKPSETTAAVVRKSWQRNIDTGKQPLTTAVDPKTTEASGTVKRLWQRSAATEKLSQ
SGTAVVKPLQSSVAVSKPFQSNVAVKKTWQRSVTPENQRESNMSSNKPLASKVVVESL
VQSNTVEKMFQSNVPTEEPQKVIVATENQSQTIKVTKKSNDTSMKLSVTSETTKVPPLA
ATALQSDVSTEKPSQTDMPTITPSQIPEPTEKPSESAFNAEKLSNVDTATEKPLQSMITEK
VESVAATLKPSQSDTAPQEILERNMDTEKILQSAMAVEKPPPTNLITEKPSIKDASEEPVQ

TNEQSEQPLKTEEVEKPHQSEKIAAETKGSEVSIENMLELESNATKLNKDHSEPEGLSSG
TNPPDFQSNPNAGQQLESKGIVAEKEADNIMEAKNDAVAEHSSESQHVAPAEVPKEQPS
EHQKDADMQLLLEPQNEDHSGNPLEPVSDTAAKDSSEPKSDIATEKTAESQNNADQSVE
QSPEPQSDKSTEKPEVHQSSTPSDELSRLQSDAGADKLSVPSSDPESNASVSKPSESQTDV
ITMEAPELQIDALPDKATDQPVKPQDDASAKKPMGTESDAACDKPSESSSDTETLPVCH
QNSSITTDEPVQGDISYETPHQRSAPIETTPGSDTVVEDCIHHEDTSSKPSEENKAIEEPEE
VSAKLPDDHVTSEKSSEEDKENAEPSVDNAPLGKPLEANEESSKSSGDTVTPEKPLEEDE
TSAEPSESDASFGKLLEADEVSANPSEDIATPEKPLEEGVASVEPSEDNSVLDKPLKEEEV
TAKPSKDVVTPEKPLEEGSTTAERLEDNAAIGEAKEEDEVIPKPESSVALEKSLEGSEASV
EPLEDNAALEKPSEDDEANAKSSEDSVAVEKPQQEEDNVATEKPQQEEDNGVKALEED
VSPEKSANGKPLEEEDPVHEKLADADTVVEPSSQDDTATEKPSATTDTAETA
