# Supplementary material for: A genome‑wide approach to the systematic and comprehensive analysis of LIM gene family in sorghum (Sorghum bicolor L.)
Source: Genomics Inform. 2023 Sep 27;21(3):e36. doi: 10.5808/gi.23007 (PMC10584642; doi:10.5808/gi.23007)
Supplement: Supplementary Fig. 8. — Full-length genomic sequences of LIM gene families of Sorghum bicolor with 2,000 bp upstream region (Doc). [file gi-23007-Supplementary-Fig-8.pdf]

Supplementary Fig. 8. Full-length genomic sequences of LIM gene families of *Sorghum bicolor* with 2,000 bp upstream region (Doc).

>SbLIM1

GATATAAGTCTATAGGAATTGATAACATATACACTAAAATATGTTTTTAAATACTAA
TGCAGCTTAAAAAATGGAGGGACTACCTTGCAAATTGGTGTTATATATATACATAAT
TTGTAATTGGCATTACTAAAATTAGAGAAACTTAATTTAATAAAGCTAAAATGACCT
ATAATTTGGGATGAGGAAGTTGTTACTAATAGCAAAGATTAGCAAAGGTGGATGAT
CTCGTCTACCAAATAAATTTACTAGATCAGTTATCCAACATCTGCAGGAGATCTCTT
AATGTGAATCATCCCATCCATACTGGTCCCTAACTAAACACCTCAAAACAAAATTAG
ACCGCCATCTATCATTCTACGTATCATCTAACCTTGTTAACGAACCAAACGCACCAA
TCAAGTGCAAGATTATAGCGAATGTTTGATCGGTGATGGCAATTGGCAACGGAACA
GGAAGCTGCAATTTGGCATTTATCTAGCTAGTTAGTTATACTGTATTATTACCAACCA
CAAGGTCGTGCATGATTTAGCAACTACGCTAACCATGATTGTAAAAGCAAAACGTTA
CGAAAAATGAGCTGTACAGTAGGTGCAACAGGTGGATTGTACTACGATCGTCTGCC
GAGACAGAGCTGTCCACAGTCCACTGCTACGCCCCTGGTGCGTGCCGCCGCCGCCGT
GACCGTGACGGACCCGGCCCAACAACAACGAGCACGACACGCTCCTCCCTGGGCCC
CACTGTTAACTACCCGGAGACAGTACTGCGCGTATAAAAAATGCATATTTCTCCATC
CACAAATAAATGCACATTTTATTTTCTAAAAAATCAAAAAATTTAAGTTTAACCAAA
TACATATAAAAATATACTAATATTTCTTATATGTAATTTGTACCAATTAGATTAAGCA
TGAAATATACTTTTATAATACACTTATTTATATACATAAATATTTATATTATTTAATA
TATTTTTAGTTAAATTGAAAAAAATTAAGAGAAGCGAGACGTGTAATGAGGGAGTA
TGGCCTTATTCTTCACCCCAAAATCCAAAAAAGTTTTAACATTCTGCATCATATCGA
ATCTTGCAGCATGAAGTATTAAATATAGATGAAAATAAAACTAACTATACAGTTTGC
CTATAAATCGCGAGATGAATATTTTAAGTCTAATTACTTTATGACTGAACAATATTT
ATCAAATAAAAATAAAAGTGCTATAATATTAAAATCTACTCACTCCATCCCAAATTG
TAAGTCATTCCAAGAATCTTGGAGAGTCAAACTTTTTTAAGTTTGACCAAATTTATAT
TATAAAATAATTATTATTATAGTACTAACTAAGTATCATTAGATTCTTTCTTAATTAT
ATTTTCATAGTATATTCACTTTACGTTACAAATCTTAGTATTTTTCTCTGTAATTTTGG
TCAAACGTAAAAATGCTTTGACTCTTTAAGATTTTTAGAATAACTTACAATTTAGGA
TGGAGGGAGTAATTTTTTTATCTCAGCAAAGCCTATGTACTACGCGGTACGCCTCGT
CCTTTCGGTCTCAATAAAAATTTGGATAAAAAAACTGTGGGAGAGAGGGCGAGTCG
CGAGAGACTTCATCAATGCCAGCCAGTGCCAGAACCAATCACGAAGAAACAAGAGG
ATAGTCCGACGAGGCAGCCGGAAAAAGGCTGGCCGACGTGGGCCGCACCGCGCCTG
CCCGCGCAATAATATCTCGTCTCCCCGCCAGCCGCCACGCCACAGATCCTGACGCCT
GGGTCCCGCTCGCACCCCCCCTCCACCCATGCGACCAGGGCTCGCTTCTCCCGTACT
TCTACCCGGTCCCCCCACCGCAGCTGCGGAACTCCTTACTCGCCGACACGTGGGTCC
CAACCGGCGGTGGCCCCGCCACCAGTGACCCGAGCCCTCAGAGCATCCTGTGCGCG
GAGCTTCCTCCTCGAATGCGCACTAATGGTGGTAGTAATAATTGCTCACTAGTTTAA
TGAGCAGCAGCACGGCACTGTGCTGTGCCTCCATCATCATCATCCGATTCCCACATC
CCTCGTCGTCGCTTGTTTGTTGCCGCTGCTGCTGCCTTTTCCTTTCCGCCGCGGCAAA
AGTGAGGCGCGGGCGGGCGAGCTGGGGGAGAGGGAGACGAACCCATCATAATTCC

ATCGGTGCTCCTGCGTATATAAGCGCGGCAATCGGGACGCCGGGCGAGGAGGTGTT
CATTCCTCATCCATCCCACGCCACGCGCGCGCAGGCCAAGGCCGGATCGGGCTCTCA
GTCAGATCTCGCCCGGCGGCCACGACCCGAGAGAGAGAGAGAGAGAAAGAGGGGG
GTTGGCCGGCGATCGGGAGAAGATGTTTAGCGGGACGCAGCAGAAGTGCAAGGTGT
GCACCAAGACGGTGTACCCGATGGACCAGCTCTCCACCGACGGCGTCGCCTTCCACC
GCTCCTGCTTCAAGTGCCAGCACTGCAAGTCCACCCTCTCCGTACGTTCCCTTGCGC
ATGTTCTCTTGTGATGGGTAGGTGGATGGATCTGTAGGGGCAATGCCATGTAGCATC
GGGTAGTAGATCTGCGAATGGTCGCCGCGAACGGATCTGGTGCGAGTTTCCTAGTAT
CAGGAAAATTCTGTATGCGCAGAAGAGATCATGATTTTGTCCGTTTCCCATTTGCTA
ACTTGGTAAAACAGAGAGGATTAGGCAGGAAATCGATGCAAATGAAAACTCCATTT
TGATCTTCTGTTTAGGACGACCTGTCGATGTATGTCTAGGCAAAGATAAGCAGTTGT
GTAAGGTCTGACAGCCTCCGTCTTCATCCTTTTCGCCTTCGATAGCTGAGCAACTATT
CCTCGTTCGAAGGAGTGCCGTACTGCAAGGCCCATTTCGAGCAGCTGTTCAAGGAGA
CCGGGAGTTACAACAAGAGCTTCCAATCACAATCACGTACGCTTTCATTCAGTACCG
AGCATTGACATGAAAATACAAGTTTTGTGTTCCTGTGATCTGATGCAGTGATGTGC
TCGTGGTTTTCTTCTTTCAGCCGCAAAGATTACTCCGGAAAAGTTGGCCCCTGAGCT
GGTGAGTGCTAGTCTGTACGCTTTAGTCTTTGTCTCATTGTATTCTTGAAATGGAGAA
GTAAACAGTTTCGTTCAAAATCTGCTATCCAGACCAGATCACCAAGCAAAGCTGCAA
GGATGTTTTCAGGAACACAAGACAAGTGTGCGACTTGCGGTAAAACCGCATATCCTC
TTGAGAAGGTAATTTTGATCACATTATGCATTTTTACTTTTCATTATTTCCTTTCAATT
TTTTGGGATGTAGCTTGTGAAACTCTGGTATCTAATTCAGTAGTTCCTCAAGGTCAAT
ATGTGCTATGGAAGATGTGGGTATCAATGGCTTTCACAACTGAACAAATATTTGTCC
CCTTCTAGTCATGTTAACCTGGTCGCGCTGTCAACCTAAAATTTGCTCCATTTAACAA
TAAAATTATATCTTTGTTCATCTTAATTTGTCATTATCCTTTCTTGATTGCCTTGAATC
ATCAAAACTCTTTCTGATTGTTTTCCTTGAGCCAACAAAACATGTGGACAGTGGTGG
TTCTAATGCATGGCTGTTAAAAACATTGCAGGTAACAGTTGAAGAAAAGGCATACC
ATAAGTCATGCTTCAAATGCTCCCACGGGGGCTGTGCGATTACACCTTCCAACTATG
CAGCCTTGGAGGGCATCCTCTACTGCAAACACCATTTCTCTCAACTTTTCAAGGAGA
AGGGAAGCTACAACCACTTGATCAAGTGTGCTTCGGTCAAGCGCGCTGCTGAAGCA
CAGCCAGAACAACCAGCCTCCGATTCCTCCTGATCGTGCCATTTCAACAGTGGGGGT
TTGAAGGCGAGAGTTGGGAAGCCTCCTGTTTTTACTTAAAAACGCATGCTGTATGTC
ATTCATCTATGCCCTGAGGATACCGGACTATCTATTTTGGTATCCTTTGCTGTGCTAG
CTGGATCACATCGGTGAACCATGGAACTGATGTCATTTCCAATGAAATTGTTGTGCA
TCAAGTAGTGAAATCTTCACTGGAATTGTTGTGCAAGTAATGAAATCGGGTATCTTG
CCCTTATTGATCGATAGTCCTATGTTGTTTTGTTGTCTT

>SbLIM2

CATACATGCCAGCAAAAAAATTAAACAAACAGCCCTTAGTTTTTTAAACAATAACA
AACATAGCATCTCCAAGAGTACCCTATTTCCCTTCCCAATCCTTGGTTTTGGCAAGA
TAAGAAAAATTCCCTCTCCAACAACTCATAATCCTTCCTCTTAAAATTTTACGCACTT
GGGAAATCTTCCCCCATCACGCATAACTTTACGTAGAGTCATGGTCGTGCGGACTCT
TCAGCTAATTATAAAGCTGAAAGGGCAGAAAAAGAATAAAGCGTATGAAATAGCTC
TATATGTAAATGTATAAGAGAGAAAGATTATATAAAATTGGTTAAAATAATTTAGA

ATTAATATAGGTATTCCTGATGTATAATTGTCTTCTATATTTTAGAAGTGAATTATTA
GAAACTATTGGATATGACCTTATTTTTTCATTCAATATATTTTTAGGATTCAAAAAAC
ATGAATTTTTTGAGAGGAAAATTTAGTATACTCTTGAAGATGCTCTTAGTTGCCATCC
ACATCAATGTCCACTGTTCGGCGCATCGGCACAGCCCGTGGTTCATTGGGCCGCCTG
ATATTTTTTGCACAGCACAAGCCCATGGACGTGTGCACAGAACAAGGGGCGCTGAT
CAACGACACCAGGCATGCATTTGCTTCCGCATGGACAGAACTCGCAAATTGATGAA
GCAGCCGTGGGAGTCCGTTTAATGGCCCATAAAAGATAGTGGCTGTATATGATAGG
AAAAAAGTCCATTTTTCTCTTTCAGCTCTTGTAAAATTTTGTTTTTCTTCTCTAAACTT
TTAAATCAGGCAAACTATGTTCCTCAATTTTTAAAACCATACGTTTTACCTCCGAAGC
CTGGTTATAAGCGATTTTAAAGGTGGTTTTGTCTTTTTCTTTTTTATTTATTTCGGCTA
AATCTTTGAAAAATCATAATAATTCAAAAAAATTATAAAATAGAAAAACTAATTTTG
TTGAACTCTACATGAGTAGACCTATATAGTGAACATATAATATGGTATGTTTTAGCA
TAAAGTTTTTGCTATAGATTTTGATCTATTCTTTTTTGTAATTAATTAGAATAATTCAT
AACTGTAATTTCTATGGGCCAATTGTGGTCAAAATTTTATGGTGGCCTAATTATTGTA
TGCTTGATGTGTATTAAAAATTTCAAACTCATTGGATAATGTCTAACTTAGTTATAGA
TTTATTTAGATATAACAAGCATAGCCCCCATCAAAGAAAGTTTTAGGATCTGTTTGT
TTGAGGTGCAAGCTGCACATTTTGGTCCTTTAGCTCAAAAGCTGCCTTTGTGGCTTTG
GGCTGTTTGTTTTTCTATTGACTGTAATAAGTTTTTAGTTTCTCAGGAAACCAAAATT
CACAAAAACATCTCCCAACGTGCTTCTTAGCTTTTAGTCTATTTCCTTATAAGTAAGA
CTTTTAGTTAACTTTTTAAAATCCAGTTTAATATCTGGACTATTTGTTTTAACTTCTTT
TTTTATCACAATTTGTTTTTACTTCTGACTTCTAGCTCAGAAGTCAGCAAAAAAAAAC
TAAACAAACAGGAAGCCCGTGGTTCGCTGGGCCGCCACATAGTGCTCCGCTGTTCG
GCGCAGCGCCACAGCCCGTGGTTCGCTGGGCTGCCCAGCACAAGCCCATCAACAAC
ATCAGGCACGCATTTCTTCCGCCCGTGAGAGGGCCATTAACAGAGCTCGCAAATTGA
AGCAGCAGCCCCTGAGTGTTCGTTTAATGGCCCATAAAAGGTACTGGGCTGTACTTA
GGTGATAAACAGGCCGCTTTGCTCGCTGCAGCGTCCTACTTTTTTGAAGGAAAAAAA
AAAACAACAACGGGAAGAACTGCCTAACACGACGGTGGCCGGCGGTGGCAGCCGA
AATGGGCAACATCATTCTCACCACGTCCTGCTGTTCTTCGTGGCTCGCACAACCTTTC
AGCTTCAGAGGAAAGAGCGTCTCGTCCATCCAGATTCCAGACACGCCCGGGGAAAT
AGGATAGGAGAAAGGCGCCGGGACCGGGACAGACAGACCGCGGAGACAAATCAAT
CTTTGCCTGAATTTGTGTGTGCGGCTTAGAAGCCCTCGCGCCTCCTATCTTTGCTCCC
GAGCTCACGCGCTGTGTCCTTTAAAGCGCATCCGTCTCGCTCCCGGAGACGTCAACC
TCTGCCTTGTTCCTTACCGGCCCCCGAATCGCGAGCGAGGCGAGAGTTCCTCTCCTC
CGTCCCGCGCCCGTTTGCTCGTCGATCGATCGGTTGTTGCCTGCCGGCCGCGAGCAC
GAGGAGCGGCTAGCCAGACGGACGTGTCGTCTTGGCAGGTGCAGCAAGCGCGGAGC
TCGATCGCCGCCATGTCTTTCACCGGCACGCAGGACAAGTGCAAAACCTGCGACAA
GACGGTCCACTTCATCGACCTCCTCACCGCCGACGGCGTCTCGTACCACAAGACATG
CTTCAAGTGCAGCCACTGCAAGGGCACACTCTCGGTACGCAACGCATATATAATGC
GCTGCTCATTTCCGTCGAAAGAAATGAAGCTTTGTGTTATTTGATTGATTGATCTCGA
CAGATGAAAGTGTAGAAATGTGGTGGGCTCATCATTTTCATTTCTGCATGCGTTCTC
CTCTAGATTAGCAGCTACTCTTCCATGGACGGTGTCCTGTACTGCAAGACGCACTTT
GAACAGCTCTTCAAGGAGACAGGGACCTTCTCCAAGAAATTTCAAGGTAATCTACA
GCGCATCAACTTGTCTATGCTGTAACGTTCAACTCTGGTCCTCTGATTCAATATACCG

GGACGTTTTGTACCTGAACCTGTAAAAATGTTTACTGCACATTTGTCATCTTCTGAAT
GTTACACACATCAAACATTCTGATCCTTCCGTTGTGCTACACAGGTGGAGCATCTTC
AACCAAGACCGACCAGGTACGGTTCCTCAAATATTCTAAACAGATGATACAATCTG
ACATCAGTAAGAGTTGACTAGTCATGCACGATGCCAATTCGAAAATCTCTGCAGGCA
AAGGCTCCGAGCAAGCTATCATCTGCATTCTCTGGAACTCAAGATAAATGCGCAGCC
TGCCAGAAAACCGTGTATCCATTGGAGAAGGTACAGAAATGCTGTTTATTTCTGTAA
CTCTGGGCGGCAAACAGTAGCACACAATTATCTGATCAAATCAATCACCACGACTA
ATGCTAATGTTGGCACCTACGCTTGATCTTCTTGTTTAGATGACGTTGGAAGGCGAG
TCTTACCACAAGAGCTGCTTCAAGTGCTCGCACGGGGGCTGCATCCTGACAACCTCC
TCCTACGCCGCGCTCAATGGGATCCTCTACTGCAAGATCCACTTCTCGCAGCTGTTC
AAGGAGAAGGGCAGCTACAACCACCTCATCCAGACGGCGCAGACCAAGAAGAACG
AGGCTGCGGAGGCCGCACCGGAGGCACCGGCGGATGCAGGCGCGGCTGAGCCAGA
AGCAGCGTAGATAGCAGCAAACGCAAAGAAAAATAGAGCCGTGTGTGTGTGTGTGT
GTGTGTGTCTATCTCCTATATATACGAACTCTGCATTCAGTAAGTAGTTGTCACAAAT
ATATGTTGTTGTTGTTATGTTTACCTTGCTCTGGCTTATTCTGTATTATGAGGCTCTGG
ATTCATGACGATGAAATGAGAAT

>SbLIM3

CCCGTTGGTTTGCAAGTCCCTAACACAAGCTTGTATTTACATTCATATATTTGTGCTT
GTGTAGTTGCTCTTGTAATTAGTTAGCTTGTGTAGCTTGCTAGTTACCTTCTTGCTTGT
GTAGCTAGAAGTAGTTCCCTTGCGTGACTAATTTGGTTTGTGTAACTTTGTTAGTCAC
ATTTCTTAGTTTGTGTAGCTAAGTAAGTTGCGCTCTCTAATTTGGCATTAGTTGCCTT
GTTATTGAGCTTGCTAGTGAGCTTAGCCTTTGTGCGCTTTGCCTCACTAGTTTGTGTA
GGAGCTCCCCCGTTTGTAAAGTACTAGTTGCATAGGTTTGTGTAACCTTGTTTCTAGA
TTTGGTTAGGTGAGCTCTTGCTAAGGTAGCACCTTGCTTGTTTGTTTAGGATCTTTTC
AAGGTGCTAGAGAACTTAGATAGAGGGGTGTAGTCTTGGCTAGACCGATAGTTTTA
ATTCCGCAACTGTTTTGGTTAGCCGGCGTGTTAAAGTTTTAGAAAGGACTATTCACC
CCCCTCTCTAGTCCGCTATCTCGACCCTTCATCCATCTGAGGTCGTTTACAAAATTTG
AATTTCAAATTTGAAAAATTTAAACGTAGTTTTTGCATGACAACATTATTTCAAATC
AAAAAGTTATCAACTACAAAGTTTCATAACTTTTCGAGACCTACAAAGTTTCATAAC
TTTTCGAGACCTACAAAGTTTATTTAGTCAGTTTTTTCATCCGAGGTAATTCCAGAAA
ATCAAAATTAAAAATTTTCAAATAAAGACGCAGTTTTAAATGATAAGATAAACTCA
AATAAAAAAAATTGTCAACTATAAAATTTCATAACTTGTCAAAATCTATAAAGTTTAT
TTTGGTTGTTTGATCATTTGCTCATCTGACACGGTAATTCTAATATTTTTCACAAATC
TTATATATCTCTCTTGTAGTTAAACTATAAGAGAGAGATAAAATTTGTGAACAAATT
TATTTTCACTTTACCAAATGAAGAAATGGCCAAAATAAAGATTATAGATCTTGAGAA
GTTATACAACTTTGTAGTTAAAAACTTTTTGATTTGAATTTGTTTAGGGCTTCAAATT
TTGATTTGAAATTTTCTTTGCCGAGTGTTTCTTTTTTTTCACTCGGCAAAAAGGGTTCT
TTGCCGAGTGTCAAAAAAAAAAAACATTCGGCAAAGAGGGTTCTTTGCCGAGTGTCA
AAAAAAACACTCGGCAAAGAGGGTCTTTGCCGAATGTTTTTTTTTAACACTCGGCAA
AACACTCGGCAAAGAACCCTCTTTGCCGAATGTTTTTTTTTAACACTCGGCAAAGAA
CCCTCTTTGCCGAGTGTTTTTTTTTGCCGGGCGTTTTTCTTTAGCACTCGGCAAAGA
AGCTCTTTGCCGAGTGCCCGAAAAAAACACTCGGCAAACCACTAGATACTCGGCAA

AGAGCCGGATTCCGGTAGTGAATTATACATTGCAGATCGGACTTTTTTTTTGGCAAA
AGAATCTGTTGCCCCTGATCTTTCATAATATTTATATCTTTACCTCACCTCCCCCCCA
ACCCCAAAAAAAAAGACACTCTCTTAAAAATGCAATAAGCTAATTAAGAAACCCAA
AACGATTACTGGGAAGAACTCAGAACAACTACCACCAAATCTTTTTGTCCAAATTGC
GTGCACATACGTAGCTGACGTGTGTAACGCATGCAAGCAGTTCATTCGACTCATCCG
AATTCCGAAATGAAGGGAGGAGAGGGCCACGCTAGAGAACATAAGGAAATGAAGG
ACGGTAGCGCATTATTCTTGTCCCGGCAATGTCCTGAGCTGAGATCACGCACCATCC
CTCTCATTCTAAATCAAGCACTCGCTTGACCAAGTCTATGCGGCGCAGAGCCCGCCC
GCTCCCTATGCTTGCTCCGGAGCTCTCGTTCGCTGCCGCGGCTTCTCCGTAGCCATCA
CTTATTATCCCCAATCCTAGCTTCGTCTATTTACTCGCTTACCCTAGCGGAGGAGAGA
AACGATTGCGGATTGCATTGGGGGCGACGAGTGCGTGCGTGCAGCCACAGGCGTCG
GCACCATGTCTTTCACCGGCACGCAGGACAAGTGCACGGCGTGCGACAAGACCGTC
CATTTCATCGACCTCCTCACGGCCGACGGCGTCATCTACCACAAGACATGCTTCAAG
TGCAGCCACTGCAAGGGGATCCTCTCGGTACGTGTGCATCATGCAAATATAATGTCC
TGTCGTCCATGGCATAGAAAAACGATCGAGTTTATTTGCATCCCATCTCTGCGTGAA
TGCAAGCCACAAAAGCAAGAGTTTTTCTGACGCTGCAAACCGAGTGATGTATAATG
CGTTGGTCGGCTGCAGATGTGCAGCTACTCTTCCATGGACGGTGTGCTGTACTGCAA
GACCCACTTCGAGCAGCTCTTCAAGGAGACCGGGAGCTTCTCCAAGAAGTTCACGC
CAGGTAATGCAGCACTACGACGTGATCTTCGGTTGTCACAACAATTCTGTTTAATTT
GTCGTGCCGGAGAGAACATTATGCTGAAAACTTTGTTGTGTAATTCAGGTTGCAAGT
CAGACAAGGGTGAACTGGTACAGCTACCCTCACAAGACTAGTATCATCATGATTGAT
TTTGGAATGTCTCTGTTTATTGTAGTAACTGGATAAATGTTTGCAATGCAAATTGGA
ATCCCTTTGCACTTTGCAGGCAAGGGCCCCAAGCAAGCTATCGTCTGCATTTTCTGG
TACTCAGGATAAGTGTGCAGCATGCCAGAAAACAGTGTACCCGCTGGAGAAGGTAC
TGAAATGATGAATATTTTTTTCTTAGTACTTACTTCCCAATATCTAATAATAACAAAT
TGCCATACTTTTGGGAAACAGTAACAACAGTCAATAATTTCTCTTGCCCTACTAAAT
TCTAATGAATGTGTAACGCTAACAAAATTGTTCTATTGTTCAGTTAACTTTGGAAGG
CGAGGCCTACCACAAGAGCTGCTTCAAGTGCTCACACGGGGGCTGCATCCTGACCA
CCTCCTCCTACGCCGCGCTCAACGGCGTCTTGTACTGCAAGATCCACTTCGGGCAGC
TGTTCATGGAGAAGGGGAGCTACAACCACATGAAGAAGAAGAGCACGTCCCAGGA
GGTGCTGCCGGACTTGGCTGCCGAGGAGCAACCTCCGCAACCAGCGGCACCGGAAG
ATGAGAAAGGAGAGGACAACTAGGAGCAAGCGATGATCCATATATATCACGCAGAC
AGAAGTTTGCGTCTGCCATTTTTCTTTACGACGCTTCTTTATTCCCTCCTCTTTTCTTT
TCTTAATCTTTCTGTGAACTCGGTTTGTCATCAAGCTGTAAGTGTACCAGATGCCCTC
GTTACGTCTGAGGCTCATGACAATGGCAATTTTGGCATGGAGCTGTGTTAGCCTCGC
GGAAAAATTGTAACTTTGTAAGCAGAACTCTACTACTTTGAATATACGATCCTAACA
ATGTTG

>SbLIM4

CCTATGAGAGACAGCCTAACAAGAGCGTCTTATGTGCACTACTGGAGCTCGTCTAGA
GCGTTGCGATTAAGGGTTTAGGGTTAACAACTGACGACATGCTTAGACCATACTAAC
ACTAGGGCATGAACACACAACAAGTGTGGTGATGCATGATGTGGCATATGCATGAC
TGTAGTTTGTGTGGCAGCCACCAAGTCACGTCTTCGGAGAAGCAGTGCACTTTACTC

CAGTTGCAAACATGAGAGACAAACACAGCCCATCTATATTCAGGGCTTGGGACCAA
AATAGATTTTCGTTTTCGCCAAACCGTCTCACTAAACCATCATTACTTGAATTATATG
TCCATCAATCAATAAGAATAATAACTACAAAACAGAATCATCATCACCACCTCATGG
ATATGCATGATGCGCGTGGCACTGGCACGATCGACCTGAAGGCAGAGCACAACATA
TACGAGATGAAATTAGCAAGCTGGGACCCACACACACGGAGATCTACATGCCACCA
GACATGCAACATCCATCTTTATTAATCGGTGGGATTAGTACTTGTCTTTGCAAGAGC
AAATCATGAGCAATGAGTAAGGCTGAAGGTTGTAACAGAACGATCTGACCTGACAG
GGGCCTGATGCAGTCTCACTTTTTTCAAGTCTGCTCGGGTGGCCTTGTCGTTGAGATC
GGCTCGCTCCTCTTCTTTTAATAGAAAATATCAACTTAATTGCCAATTAGGGTTGTTG
GCTTGAGTTTATCTACCGAATCTGTAAGTTATTCAGTATTTTAGCCATCACTTTTGAG
CCAAGCGAGTAGGGCATCGCAGATAGCTATGCTTGCAACATATTATAGACCATAAG
CAAAAAATATTGTTCTATTACTGAAAGTAGTGCTGATAAGTTGAAGCAAACAGGAT
GAAAGATAAGTAACAAAAAAAAAATGCTATGTTTGACCTAACTAGGACATCTGTTCTC
AATATTAGCTGTGAGATTTGTCCTGTGCTAAGCTTGCCGACACCCTAAGGAAACAAC
CGACAAATTCATGAAGCCAGAATATCAGGCTGAGTTCCGAAGCTTTAGAAAAGATG
AGATGTCACTAAATCCTGCACCCACAGTATTTTAGAGGGTCATGACAGAAATAAAAT
CTTTTTACATGCGATGAATATGCACAAATTGCTGACACTTTCTTTTTCATGCACTCAT
CTTGATATCAGTAGCTTATGTTCCTCAATCAAGAAGAGCAAAGGAGTGGAATATGTT
AGCTCTATACATATAAAATTCATGGATTTTATATAATTCATGGAATATGAGTAAAAA
GTTAAGCCATGCTTAGTTAATTGAAATTCATGGATTTTGTTTGTTTCTTTTTTTTTTAG
GAAACAGGAGAAGCCCAGAGACCTCTACCATTTGTTTGTTTCTTTGCGCATGAATTT
TTAAGTAACTACGAAAAACAGATACTGCCTGCACAACTGATCATCAAAGAATCTCTA
TTCCTTATTTTTCTCCTAGTACTTTGGTGAGAAGCAACCCCTCTTGAATGACAGAAAA
AGAGCAAAAAAGAAATACCAGTTTTGGGTTTCACTGCACTAGAAGCAACCAGGCTG
TGAAGAATCACATAAATCTCTGGTTCACTGCACATGCTGTGGCATCATGGTATGGCC
GTATGGCAACCATGTTCCAATCAATCAAGCGGAGCAGAGATCCCCAGAACCGGTTC
CGCTCGAGGAGGGCTCGTCAAGATTTACCTACTCCACCTCACCTCTAGAGTAGAATC
CGAAGATGCAATGCAACAAACCAAGCTGCCCTCTGGTTTTCACTTTCACATTTCACA
CACCTGCACTGCATGCTCTGAAAACATGAGACACATCACACATCTCTCATTCTCTCT
CTCTCCTCTCTAATAATTGTCGCAAACTGTTTTCTGCCTCCCTTTTTTGTCACCGTCAG
TCCTCACTTGCACCTGATTTTGCTTCGCCACTCCGCAAATTTCCACAACCACCACACC
ACAACATTTCCTTTCCTCCTCCCCATTCCCATTCATGCAGGACCGACCCCCCACCTCG
TATGCTCTTTCTATCTCCTCCCTCCCTCCCTTCCTGACACCCCCAACATTCCCTGCCCC
CCCTCCTCCCTCCCTCCCTAGCTGTGTCCTGCCTAGCGGTATCAATACCTGGCTGCAG
CTCACCACCCACCAACCACACCACACCAGAGTTCCCAGCTCCCATATAACCACTGAT
CAGCAGCAACTCAAGGCTGGCTGGGGAAGTTCAGAGTGCGGCTACACCATAGTATA
CCTCTGTAGGCAGATCAGTGTGTCGTTGCAGAGGAGGGAAGAGGACCAGCCATGGC
GACCTCCTTCCAGGGGACGACCACCAAGTGCACCGCCTGCGACAAGACGGTGTACC
TCGTCGACAAGCTCACCGCCGACAACCGCATCTACCACAAGGCCTGCTTCCGCTGCC
ACCACTGCAAGGGCACCCTCAAGGTAGCCACAAACCTGCATGCTTCAGTTGATCGAT
CGATCTGTATGTAAAAAGCTGAGTCGAGTGAGATGATAGTAGTGATTAGGCGGTGA
AAGTTTACCGGCATTGATTTGTTTTGTCTGAGTGAGGATGTTCTCTGCCGCAGCTTGC
CAACTACAACTCCTTCGAGGGAGTGCTCTACTGCAGGCCTCACTTCGACCAGCTGTT

CAAGAGGACCGGGAGTTTGGACAAGAGCTTCGAAGGTATCATCAGTTTTTTTCCCC
CTCCCCTTTCGCAATACAAAATAGATCCAGTGGTTCTTGCATGTCCTCACCTACAAAT
GCTTCAGGAACTCCAAAGGTTGTCAAGCCAGAAAGAAACGTTGGGAATGAGGTATG
CAACAACTTAGGATTCATGTTATGCAGAAGAGAATTTTTCTTTTTTTTTCTCTCAGTA
CCTACAAAGCTGCATTTTCACTTCAGTGCCGAGTAGCACAGGAAAAGAAATGCATTC
AATCATTGATGCACAAACAACTCCTAAAGCATGATGTGAGATGCATGGATTTTGAAT
ATCCCCATCTGTTAGAGAGGAAGAGACTGAGATAAATAGCCACTCAAAACTTAAGT
GTGATGCTTGGATCAGAACAGAGTAAATCTAGTCCAATAAAAAGATCAGATATTTAT
CAGATCACAAGAAAATTATTGATAAAACCTTTGATCTTCATGAGGCAGATGACTAGA
TCAGTGTTGTATTGTTTTAATGCTTCATATCATCCAGTTGGCACTATTCATCTTTTATT
CTTTATTTATCAACAACTGGCACTAGATGATGCTCATCATGATAGCATAATTGGCAG
AATGCTGTTAAAGTCTCAAGCGCCTTTGCTGGCACCAGAGAGAAATGTGTTGGATGC
AGCAAGACAGTCTATCCAATTGAGAGGGTAAGATTCTATGTTCAACTATCTCAATCA
TTCGTCAGTAGTTTGAGTCTTCTTCACTTGACAACCCGTCACTGAATTCTCATTCAAG
AATCAGTGAATCACATCATATAAGATTCCCAGATAAAAAACGTTATATCCATGAAG
ACAGCGATTGCTGCATTCTCAAGTTGAAAGTTCAATTATTGATATTTTTGCCTAGAG
ATAAGAAAAGGTGACAGGTATATTGTACCTTTTGAGAGTTAATTTGCGTGATTATCA
GTATGCTGATTGCAACTCACCGATTAAGCATCCAGGTTACTGTCAACAACACTATGT
ATCACAAGAGCTGCTTCAAGTGCTGCCATGGAGGATGCACCATCAGCCCTTCTAACT
ACATTGCGCACGAGGGGAAGCTCTACTGCAAGCACCACCACATTCAGCTGATCAAA
GAGAAGGGAAACTTCAGCCAGCTTGAGAATGATCACGAGAAGACGTCACAGGCTGG
GTCACTGGAGGATGAAGAAGAGTATTAATCACTGAGCACTATCACAGATGAATAAT
TAATCTTCTTGCATTTGCTTAGAGCACTATATATTTCTGTGGCATGGTTCGATTTCAA
TTTTACCAATGGAGCTCATGTGCGTTCAGAGAACAATGAAATTATCGTCTTGTATCT
GCATGTAAACCTTTGTTATCTTTGAGTCAAATACTCTACCTGTTTCACCATTTTCATT
GATCAGAAGATGCCTCTGCAGTCTGCACCAAGAAGACACAGAACTGCAAGCTCTTG
GT

>SbLIM5

CACCAAATGATATGATCCACTTCATATCATCACGTGACCGTATTGGTTCATCGATCTT
GACCTCACTTGCTCTTCACCGTTGCCTCGGTCCATCGGCGCCAAGTCTTGCTCAAGCT
TCACCGTCACACGCGGTCCCTCGCTTCAAAGCCTCCGACTTGCCCTTCACTCTTGCAA
CCGGTCCATCAAGCCAAGCCTCATCTTGATCTTCTCCACCTTGGTCACATGACTCCAT
GTCATGTATCATATGTAATGAGCTCCTCCATTATCACATCATCACCTGTGGACTAATC
TCCTGTGTATCTCACATAAACACTATTAGTCCACCTAAGTTGTCACTCAATTACCAAA
ACTAAACAAGGACCTTTCAGCCATGGACTCAATGTCGTTCACTTAGAGTTCATTAGA
AAAAGAAAAAAAATTCTACCAATGGGCTAGGAAAAAAAACTACCGGGTACACGTGT
TTGGAGAATAAAAAAAGCCAATGGCCTGACCCTGTGTACTGTCTCTTCTCTTCGGAT
GTGCTAGGAACCTTATCAAATCTCAACCAAGGGCAACCCAGAAGCAGATCGTGGCA
AAGCAGCCAGACGCAGACACAGAGCAATCAACGCATATGCAGAGCAGCCAGGCGG
AGAGATCCACGCGGAGGCAAAGAAGGCTAGCATCGAGGCAGACCTGGCTGGCGCC
AATGCGGTGAAGGGCCACGCAGATGTGGAGTAGCCAATTCGGAGAAGGCCACACGC
TTGTGGAGGTCTGCACTCTCTTGTCGTCGGCGTCGACGAGCACAGTGGAGGAGGTAA

GGAAAGGTATTTTATACACAAAGTTAGCAGGATGGTTCAATTTCTTTCGTAAGGAGT
TTGCAGTGATGTGGCTGAGGAATCAATGTGAAGATAAATGCTTGTGTCTGTTCGTTT
GTGGTGATGTGATGGTTAATATTGAGGGGTGGAGGCGGAGGGTCGGGGAAGGGCGG
CGAGGTGGCGCACTTGACCACCAAGAGGCTGCGCTGGAAGATGGGTTGCAGCTTGC
AGGTAGAGGTGGGATCCGTTTTTGTTTAACTGAAGATGCGAGAGTCAGGAAGGCGA
CGCTAGAGCACGGGGTGAAGGTGGAGGTGGGAAGTGGATGGCGTGGCTTAAGGAG
ATGGTAGAGAATTAGGATAGTGGGGTTTATCTTTATAGGAGATATTTGTGGTAATGT
GATGGTTAATATTGAGGGGTGGAGGCGGAGGGTCGGGGAAGGGCGGCGAGGTGGC
GCACTTGACCACCAAGAGGCTGCGCTGGAAGATGGGTTGCAGCCTGCAATTGGAGG
TGGGATCCGTTTTTGTTTAACTGAAGATGCGAGAGTCAGGAAGGCGACGCTAGAGA
ACGGGGTGAAGGTGGAGGTGGGAAGTGGATGACGTGGCTTAAGGAGATGCTAGAG
AATTAGGGTAATGGGGTTTATCTTTATAGGAGATATAGATTAAAGAAGTTGTTAGAG
GGTATTTCTTCACCAAAATATATATTCCTATCAATAAAGATTTAAAGAGTCTCTCAA
GTTGCTCTAAGGAGTACATTTCCATGGGATTTGTTTCTAATTTATGAGGCATATCTGG
AGCACATTGATTTTTAGATTAGAAAAATAAGGTATTCACCTCTAAATCCCAATAAAT
TTCCTATACCAAAGAGGCTCTAAATAGATTTTTAGATTAAAAAAAATAGGGTAGTCA
CCTCTAAATTCCTATAAAATTCCTATACCAAAGAGGCTCTAAATAACCAACCTCTAA
GTTCATGTTATTACAGACCTAGCTAAGTTTGTACCTGATAAATATTTTAATGTCAATA
TCATTAGTGATGACACTTTCATGATCAGACTTTGAAAGAAAGTTTTTTTTTAAAACTT
TTCCTTTCAAAAATATACGACTGACACATCCCTAAATTCCCTGCAGGCGGCCTGCGG
TGGTGTCCGTAGCAATTCCGACCTCTTTTGACAAAAGCAGGCGCGGGAGCGCACAC
CGGCAGCAGTCCGCACCAACTCGCCGTCCGCGCCTCCGTTATTCTCCCACGCCTCTC
CCTCTCCCTCCACGACACACAGGCCGCCTGAGCTGTGCTGCCCTCTCCTCTCCTTCCG
TTTTCACCTCCGCCAATCATCCACCCCACCCGTCGCCGTCGCCTCCTTCTCTTTCCCT
CTCTCCTTTTTCTCCCTCCTCGATCCTTCGCTACGACGGAGCAGCAGGAGGGCGGCG
ACGGCGAGGGGATGTCGGGGGCGTGGGGCGGCACCACGCAGAAGTGCGCGTCGTG
CGGCCGGACGGTGTACCCCGTCGAGGAGCTTGCCGCCGACGGCCGCGTCTACCACC
GCCCCTGCTTCCGGTGCCACCACTGCAAGAGCACACTCCAGGTCCCTCTCTCTTCAG
CCCCCTTCGTTTCCTCCCTCCCCTGCTCGGCTCGCGCATGGACTCTTGGGGTGTTGTC
GAGCACAGTCTTGTTATTACGAAGCGAAAGTTCACGTGTAGGATCAGTCGCACTGTT
ACTGGTGCTTTTGTGATTCTGGCTATTATTGCGAGCGTCGCTCAAGTCAGAGTAGCT
GGGCTGTCTTGTCTTGCTTTCTTGTCCTTGCTTCGAATTGCTTGGCTGCTTCCTTCAAT
TGCTGTACTTCCTTGGTCTGGGTTTGTCAGTTATAGGTTGCATGATCATGTCGACTGC
GATTGTGCAGGATTTGCGCACGGAGTTACTTGTTACGCGTTGAATTTGTACTGGTTCT
TGCTTGTTAGCGAACTGGGTACAAAATCACGGATAGGGAGTACACAGTACAGGGTA
CAGAATTTGTTGTTTCATTTAAGCACGATTGTGCCATCAGTGAACTTTTCTCTTGCAA
CCACATGGCAAGTGCGCATTTAAATCTTCAGCTGTTCCCCGATGAATACCTAATGAC
AATTGATCTTACCTTTTAATCATGATAATGTAGAGTGTGAAATAATGTCATTATCTAA
AGATTTCTGTCCTTCTATGAATATAATATGCATTTAACTTTTTGAGGAATCCATCTCA
ATAGGCTACTGATGTAATGAGATGTCATATCTATCACTTTAAGTATTAAAACAGTGC
CTTGCTATATCATGATCACTTTGTACTTGCTTTCCCTTGCACATGGCATAGCTGTCAT
AATTCTTGCAGTCAGTTGACATATAATCATGTAATAATGCAGTGCAAGACAAATGCC
TTTCCAATATGGATGTAGTATTGGTACCTGATTAGACTTGCTTGCATTATTCCATGAG

AACGTGTTGTTTGGCACAGCTGCACCTTAATTCAGAGACTTCTAATTTTGTTGAGAG
CAATAATGATCATAGTGAATCTCTTACGCTACAATCATACTAGTGGGCATCAGTGCT
ATTTTTTCTTATTTGCAGCCAAAGCTACACTTGTGAAACCTAACCAGAATAATACTA
GTTCCTGCATGATAGTGAGCTGGGTAGAAATCACAAAGCGCAAGACTTGAGACATG
CTTGAAGATTTTTTTCTCTTCTAGAAAAACAGTCTAGATTCTTGAAGCACTTTTGTTC
TTGGTAAAGTGGGGAGTTCAGTGTACTGGAATTTGTTTGTATATAATTGTTTTCCCTC
AAACATGCAGGATAGCTGTGTATCAATATATTAAGAAGAAAAAAAAGAGGGGGAGT
AGAGCCCCATACAGATCACCCGCACACATAAACAGCAGAACAAAACTCAGAAGAA
GCCTATGCTACCACTATTACTTTAGAGCACATTGTTTGACACACTGTAGCTTTATTCA
GAAGCTTTACATTTTTACAACAGCAATAATGATAATACTTATGCTACGTCAAACTAG
TTGACAATGGTATTATCTTCATATGTTTACAGCCAAAATGTGCATCTGTGAAACCTA
ATCAAACTAATACTGGAGCTAACCCCATTCTTCTGGTTCTTCTTTCTGCAGTTTAGTA
ATTATTCTTCCGTGGAAGGTGTCCTATACTGCAAGCCTCACTATGACCAGATATTAA
AATCAACAGGCAGTTTGGAGAAAAGTTTTGAAGGTATTAGAGACAATCAATTGGAC
TAAGATGCTGTGATATATATATATTTTAAATACCTTATTGTCCTCACAATTTGCATGA
GTAGATGCTACAGTGCATATGTTTCTGATCGAAATTATACAACTCCAAAAAATAAAT
CAACCCTAGGAACAGCCTCCCCAAAGCATTATGATTAAGAAGAAGCCTCAACCAGA
CAAGCCAAGAAAAAACCCCAAACCCTGGATTCACCCTATAGGGCCATAACATAATC
TGGGCCGACCACGACTTTATGGGGTTGTGACAACTGCAACTACCCTGATAGGAGGG
CCCAAACCAACCACAAGCACCACAGGCGCCCTGGCACACTGTCTTTTTGGGTTGCAC
ACAAAGGGATTTTTTTTGGTTGCCACCAGGATTTGGACCATGGCCGGTTTCTCGTTCA
ATTGGGGAGCTTACTGCTACACTACAAGAGTGTTGGCTACAGTTCTTATAGCTGCAA
TTGAATATGAAATTATATTATCACATCTTATTATGCACACGATCACAATGGTTTTCTC
TTTTCATCCTGTAAACCAGGTGCTTTATTTTAGCATTGTTTTTATAATATAACACATC
TTTGTGAGCTAACACTTTTTCAGTGGTGTTTGTGTGGGATACGACTCTCTTCTTCTAC
TAATGCAATGATACATATCTCTCCTGCGTGTTTGAGAAAAAAGCGAACTAATGTTGT
TCTCTGAAATATTACTGTGTTATATGTCAGGTGTGGCCCGATCAGCTAAGTCAGAAA
AATCAAATGGACATAAGGTATCTGAGCTTCTCTAGGGCATGCCTGGTGCCTAATACA
TTTCACTGATACTGCAATATACAGCAAATTTATGCCAATTTCCTCTATTTTTTTTATTG
TCAATGTGCAGGGCCAACAAAGCAGTAGATTCTCTAATATGTTTGTTGGCACACAAG
AGAAATGTGTAGTTTGCAACAAGACTGTGTACCCGCTTGAGAAGGTCAGTATTAGTC
AGTCTTCCTCTTTTGTACTAAACTAGAGTAAAAAATGCTTTGGCAGTTAAGCACAGT
TTGCTCCTAAGTGTACTCGCTGACAACTATTCATGTCCTAAGTGTGTGTGTGTGTGTG
TATGTGTGTGGTGGTGGTAGGTGTGTGTGGGGGGGTGGGGTGGGGGGAGTCCTCTGT
TTAAATTCATAATCTTGCTCAGAGGTATTTTATCAGAAGCATAGTTCAGCTGTGTACC
GTCCACGGATGGTAGAGGCTGGAACTTTGTTCCATTATCTGAAAAATCAGAAAGCAT
AGTTTCTTTACAACTGCTAATGTAATTAAACTTTTTATGACTATTAGTATTGGAAAAA
TTCTATAGTAATTGCAATTTCAGGATAGTGCATCTAATATAATTCTCCATTGCATGTA
ACATGTAGAAAAAAAATCTTAGTATTAAAAATTCGATCTCTTCACCACAAGCCAAAG
TGATGGTGTATGTCCTTCAGAATTCAGTGACCTCTATCATAGTACACTAAGTGCTTTT
TTTTGGGAATGGAATCATAGTGCACTAAGTGCATAGCTGTCAAAGGGTCATAGAATA
ATTTTAACCAGCCTCTTTTGCAGAGTATAATTTAAGGGCACATTTACATCACGCTGTT
ACATTTTTGCAGGTTGCTCTTAATGGAAATTCTTATCATAAATCATGCTTCCGCTGCA

CCCATGGTGGTTGTACGCTCAGCCCATCCAATCATATCACCCATGAAGGCAAACTTT
ATTGCAAGACCCACCATTCTCAACTGTTTATGGTTAAGGGGAATTTCAGTCAGTTCG
AGGACAATTCTGGGAATGCAAAAGTTGCTAGTGAGAAACAACCAGAAACTGAAGAA
GCCACCAAAAATCCAAATCAAGGTGATGAAGTCACACAGAAACCAGTAGAAAATGA
ACCTATAGATGAGAAAACTTCAAAGAATGATGTTGCAGCTGAGAAACAATTGCAAA
GTAGTGTTGATGTCACAAAACCATCTGAAAGCACCATGGCAGAAAATGAACGAGGT
ACTGAGAGTGAGTCAAAGAGTAATGTTGTCAACAACAAGCCATCAGAAAGTAGTGT
AGAAAAGCCACTGCAGAACAGTGTGGTTGATGTAAAGCCATCAGGAAACAGTGCAG
CCATGAGAAAACCCTGGCAACGAAGTCTGCAAACAGATAAACCATTTCTGAGTAGC
ACAAGCACTGTAAAGCCATCACCGAGCAGTGATGCCACTGAGAAGCCATCATCAAG
TAATGGGGTTGATATGAGACAGCCTGAAAGCAGCACATTAGTAAAAAAACCAGGGC
AGCAAAATGTGCCAACTGAGAATCCACCACAGATCGTTTTACCATCAGATAAGCCAT
CAGCGACCAGTGTAGATGATGCAAAGCCATCAGAAAGCAGCAAAGTGGTCAAAAA
ACCATGGCAACGCAATATGGCTGCTGAGAAGCAATTACAGAACAGTGCACCAACTG
AGAAGTCACATAAAAGTGTAGCTACTGATAAACCATCACCAACAACCGACATGAAG
TCATTAGATAACACCACAGAAGTTAAAAGTCCATGGGGACGCAGGATGTTCAATAA
TAAGTCACTAAAGAGCACTGTAGGTACTGAGAAATCGTCTGCAACCAGTGTGGTTG
ATGTGAGACCAGGGGAAACCAGTACAGTAGCCCCTGTGCCACAGCAACAAACTGAA
AACGTTGAGAAACCTTCAGACACCAGTGCAGATGATGCAAAGAGTGCAGATGATGT
GAAGAGTGCAGATGATGTGAAGAGTGCAGATGATGTGAAGCTATTGGTCGCCAGTC
CAGATGACACTAAGAGCGCAGATGATACGAAGACTACAGATGGTGTAAAGCCATCA
GAGACTACTGCAGCTGTAGTTAGAAAGTCATGGCAACGCAACATAGACACTGGGAA
GCAACCGCTGACCACTGCAGTTGATCCAAAGACGACTGAAGCTAGTGGAACTGTCA
AAAGGTTGTGGCAGCGCAGTGCTGCAACTGAGAAGCTGTCACAAAGTGGTACGGCT
GTTGTGAAACCATTGCAAAGCAGTGTGGCTGTCTCCAAGCCGTTCCAAAGCAACGTA
GCTGTGAAAAAGACATGGCAAAGAAGTGTAACTCCAGAAAACCAGCGAGAGAGTA
ATATGTCTAGCAATAAGCCATTGGCAAGCAAGGTGGTCGTTGAGAGTCTAGTGCAA
AGCAACACGGTTGAGAAAATGTTTCAGAGCAATGTACCTACTGAGGAGCCACAGAA
AGTCATTGTGGCCACTGAAAACCAATCGCAGACCATCAAAGTTACAAAGAAGAGCA
ATGATACATCCATGAAGCTATCTGTAACAAGTGAGACAACCAAAGTGCCACCACTT
GCTGCAACCGCATTGCAAAGTGATGTTTCCACAGAGAAACCATCACAAACTGACAT
GCCTACCATAACACCTAGTCAGATCCCTGAGCCCACTGAGAAACCATCAGAAAGTG
CTTTTAATGCTGAGAAGTTATCAAATGTTGACACTGCTACTGAGAAACCACTTCAAA
GTATGATCACTGAGAAGGTAGAAAGTGTAGCAGCCACATTGAAGCCATCTCAAAGT
GATACAGCCCCTCAGGAGATCTTGGAGAGGAATATGGATACTGAGAAAATATTGCA
AAGTGCCATGGCTGTTGAGAAGCCACCTCCAACCAATTTAATCACTGAGAAGCCATC
AATAAAAGATGCTTCAGAGGAGCCAGTTCAAACTAACGAACAATCTGAGCAGCCAC
TGAAAACTGAAGAGGTTGAGAAGCCACATCAAAGTGAAAAGATTGCTGCGGAGACG
AAAGGGAGTGAAGTATCTATTGAGAATATGCTAGAGCTTGAAAGTAATGCCACTAA
ATTAAACAAGGATCACTCAGAACCTGAAGGGCTTTCATCTGGTACGAATCCTCCAGA
CTTCCAAAGCAATCCAAATGCTGGGCAGCAATTAGAGTCTAAAGGCATTGTGGCTG
AGAAGGAAGCTGACAATATAATGGAAGCTAAAAATGATGCAGTTGCTGAGCACTCA
TCAGAATCTCAACACGTTGCACCTGCTGAGGTTCCAAAGGAGCAACCATCAGAACA

TCAGAAGGATGCGGATATGCAGCTGCTATTGGAACCTCAAAATGAGGATCATTCTG
GGAATCCACTAGAGCCTGTTAGTGATACAGCTGCCAAAGATTCATCAGAGCCTAAA
AGTGACATAGCTACAGAAAAAACTGCAGAATCACAAAATAATGCAGATCAGTCAGT
TGAGCAGTCACCAGAACCGCAAAGCGATAAATCAACTGAGAAGCCAGAAGTGCATC
AAAGTAGCACACCTAGTGATGAGCTTTCTAGGCTTCAAAGTGATGCAGGTGCTGATA
AACTATCAGTACCATCATCAGATCCTGAGAGCAATGCATCTGTCAGTAAGCCATCAG
AGTCTCAAACTGATGTGATTACCATGGAGGCACCAGAACTCCAAATTGATGCTCTTC
CTGATAAGGCAACTGATCAGCCAGTGAAACCTCAAGATGATGCATCTGCTAAGAAG
CCAATGGGAACTGAAAGTGATGCTGCTTGTGATAAACCGTCGGAAAGCAGCTCAGA
TACTGAAACACTTCCTGTATGCCATCAGAACAGCAGCATAACCACTGATGAACCTGT
ACAGGGTGACATTTCTTATGAGACACCACATCAGAGAAGTGCACCCATAGAAACAA
CACCAGGAAGTGACACAGTTGTTGAAGATTGCATACACCATGAAGATACCAGCAGC
AAACCATCAGAGGAAAACAAAGCTATTGAGGAGCCAGAAGAGGTGAGTGCCAAGC
TGCCAGATGACCATGTGACTTCTGAGAAGTCATCAGAGGAAGACAAGGAAAATGCA
GAGCCATCAGTAGACAATGCTCCCCTGGGGAAACCATTGGAGGCCAATGAGGAGAG
CTCAAAGTCTTCAGGGGATACTGTAACTCCTGAGAAGCCACTGGAGGAGGACGAGA
CGAGTGCGGAGCCATCAGAAAGTGACGCATCCTTTGGGAAACTGTTGGAGGCCGAT
GAAGTGAGTGCCAACCCATCAGAGGATATTGCAACTCCTGAGAAGCCACTGGAGGA
AGGTGTGGCAAGTGTGGAACCATCAGAAGACAATTCTGTTCTCGATAAACCATTGA
AGGAAGAAGAGGTCACTGCCAAGCCATCGAAGGATGTTGTAACTCCCGAGAAGCCA
CTGGAGGAAGGCTCAACAACTGCAGAGCGATTAGAAGACAATGCTGCCATTGGGGA
AGCAAAAGAAGAAGATGAGGTGATTCCCAAGCCAGAGTCCAGTGTGGCACTTGAGA
AGTCATTGGAGGGAAGTGAGGCAAGTGTAGAGCCATTGGAAGACAATGCTGCTCTT
GAGAAACCATCGGAGGACGACGAGGCAAATGCCAAGTCATCAGAGGACAGTGTAG
CTGTGGAGAAGCCACAGCAGGAAGAGGACAATGTAGCTACGGAGAAGCCACAGCA
GGAAGAGGACAATGGTGTCAAGGCATTAGAGGAGGACGTGTCCCCTGAGAAATCAG
CCAATGGGAAACCATTAGAGGAAGAGGACCCAGTCCATGAGAAGCTGGCAGACGC
CGACACAGTCGTTGAGCCGTCATCTCAGGACGACACTGCCACTGAAAAGCCCTCAG
CTACAACTGACACTGCAGAAACTGCATGAGACAGCAGTTAGCTGGTTACAGTTACTA
GTTAGCAACAGTTTTTAACAGAGTGTGGATCGAATACTATTTATTGGTGATAGTTCG
GCGTGCCTTCTGTTTGTTTTTTTTTTGGGGTTTTGTACATTGCTTGAGTTGTAAAATTT
GAGGGAAAGAAACTATAAGTTTTGATTTGATTTGATTTTTTACAAGCAATAATCCCC
TCCTCTCCATGTTAGGAGGAGGATACCATTGAAGTTCAATATATAGGATTTGTGTGC
GGTTTTGATTTTTGTTTGGATGGTGTACTTTGTAAATGATTTTGTGCAAATATTACTG
TGCTTGCTCATTATTTGGTTTTCTGCGTGTAAATTGATCTAGCAAGATTTTATGTGCA
AATGGTGTGGCTAGGCTTTCTATAATCA
